# Supplementary figures and images for: APP β-CTF triggers cell-autonomous synaptic toxicity independent of Aβ
Source: eLife. 2025 Apr 23;13:RP100968. doi: 10.7554/eLife.100968 (PMC12017768; doi:10.7554/eLife.100968)

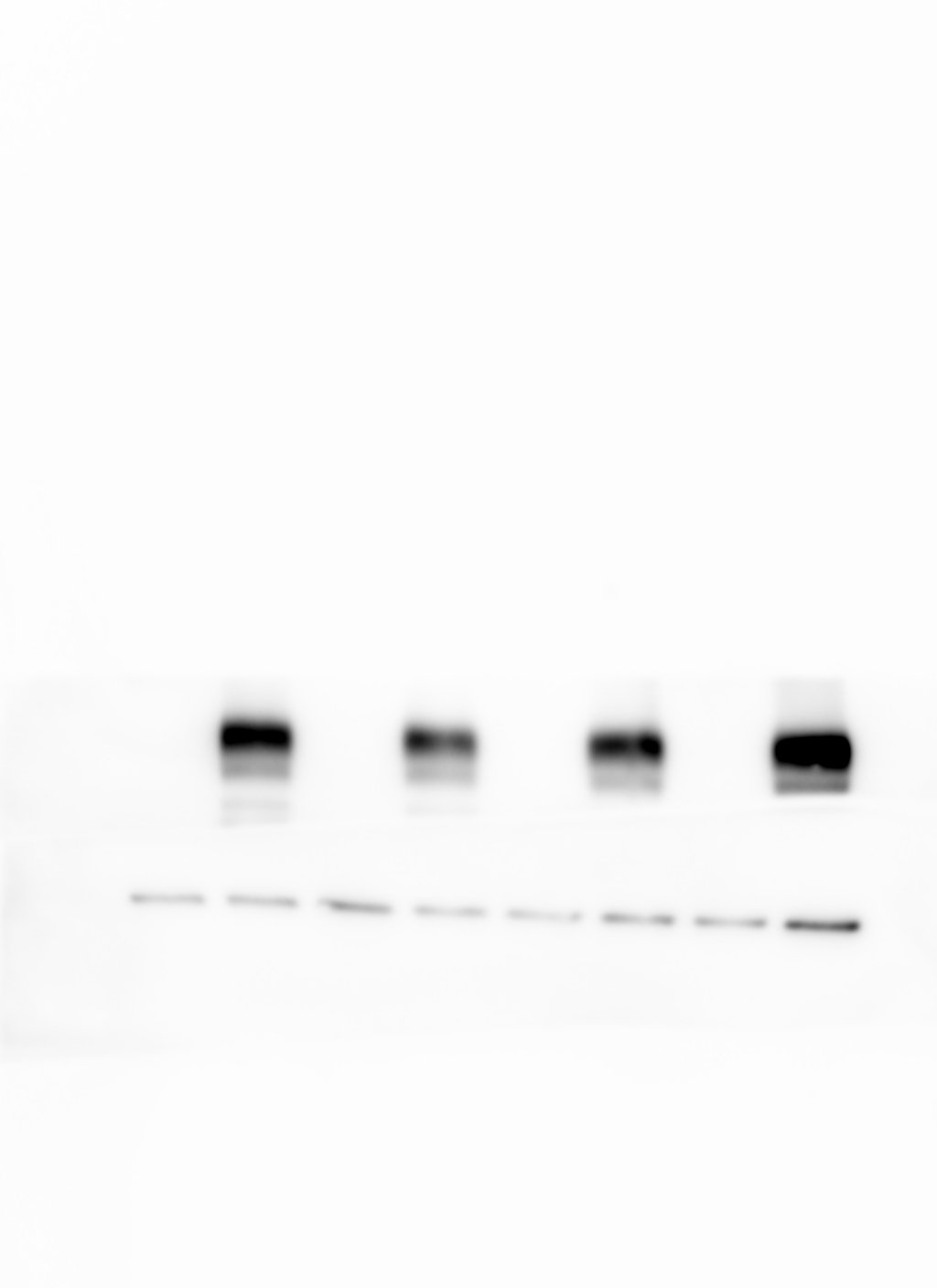

Supplement: Figure 1—source data 1. [file elife-100968-fig1-data1.zip › Figure 1G/BACE1-unedited gels.jpg]

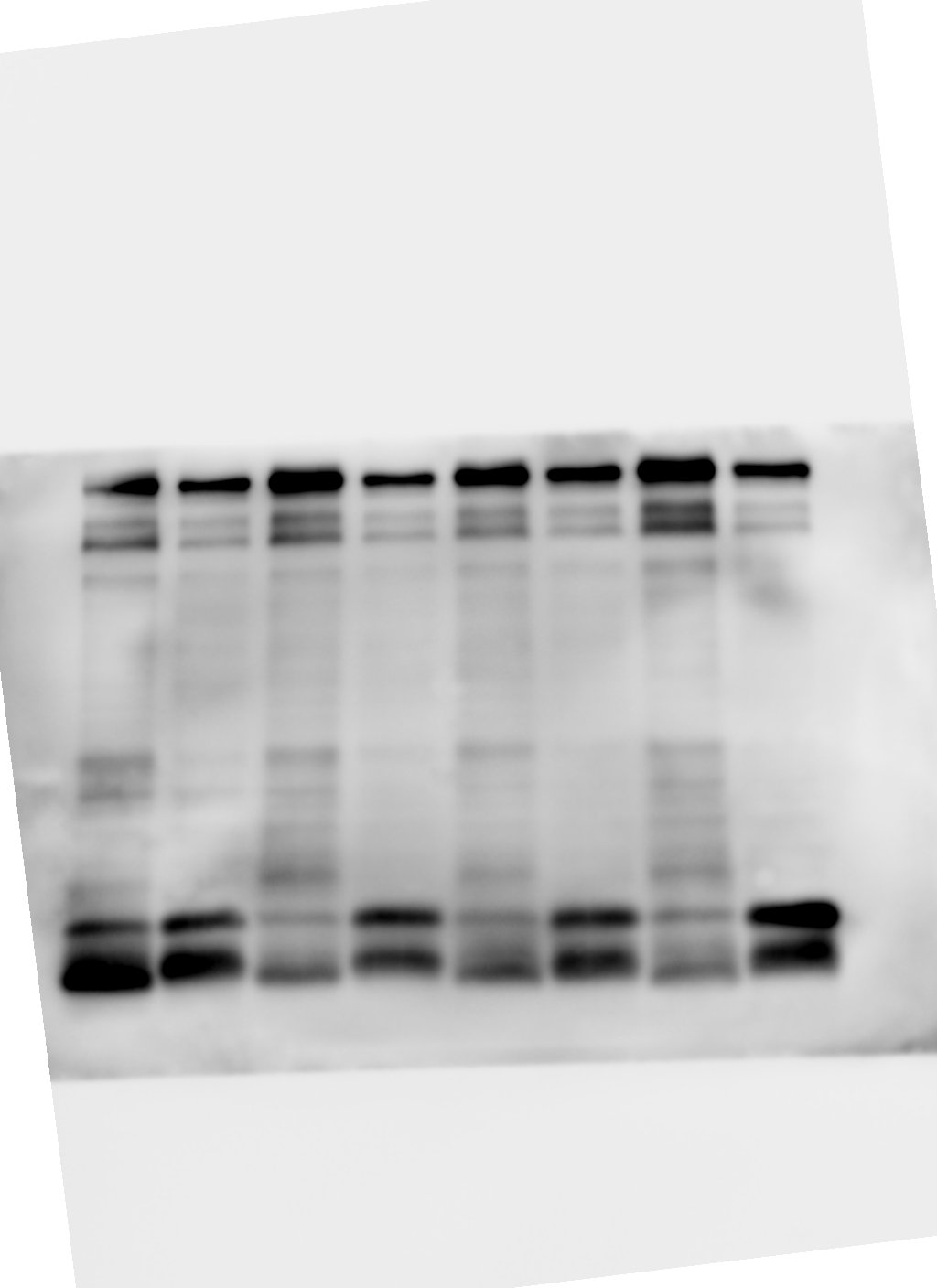

Supplement: Figure 1—source data 1. [file elife-100968-fig1-data1.zip › Figure 1G/CTFs-unedited gels.jpg]

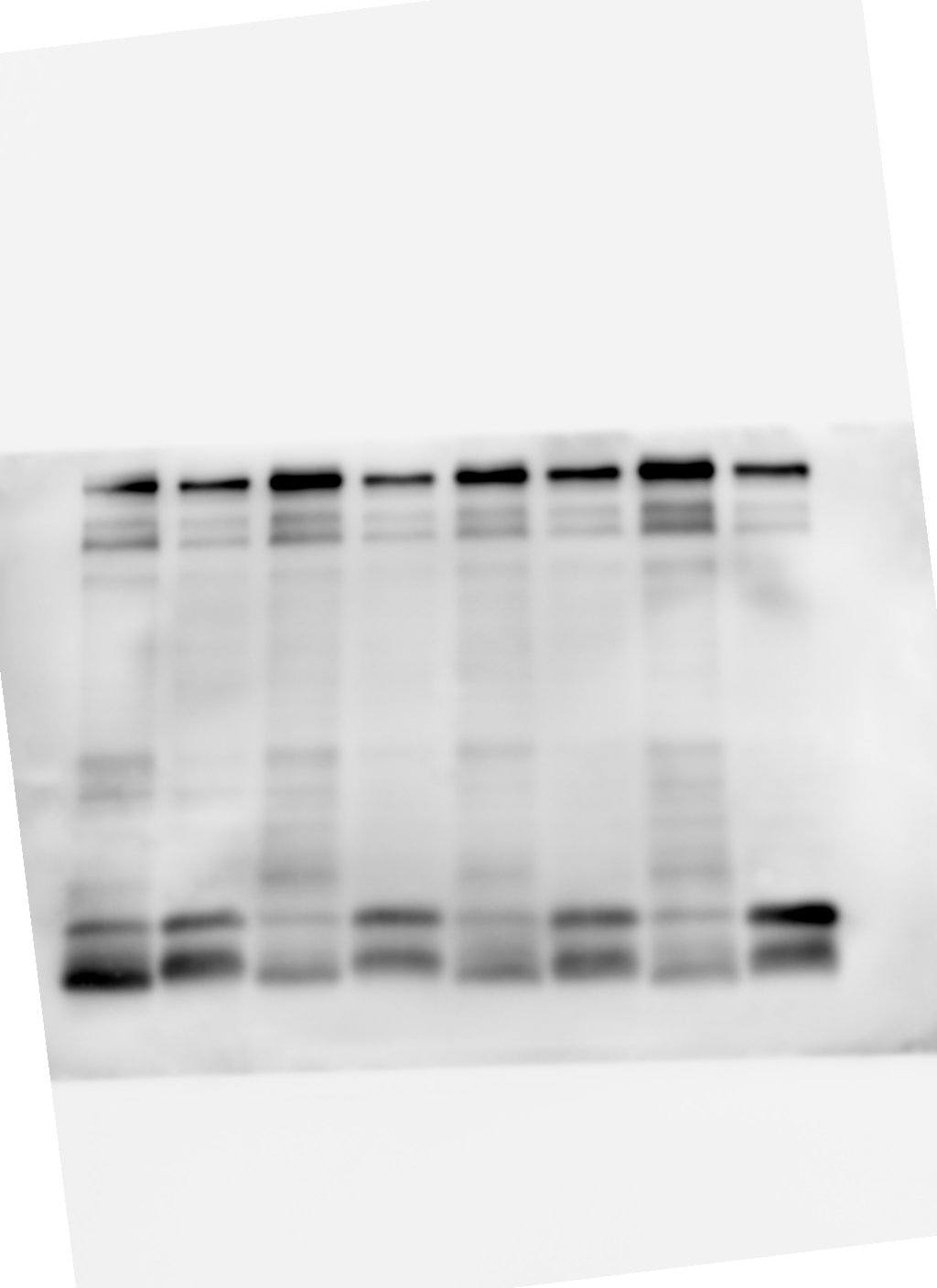

Supplement: Figure 1—source data 1. [file elife-100968-fig1-data1.zip › Figure 1G/FL-APP- unedited gels.jpg]

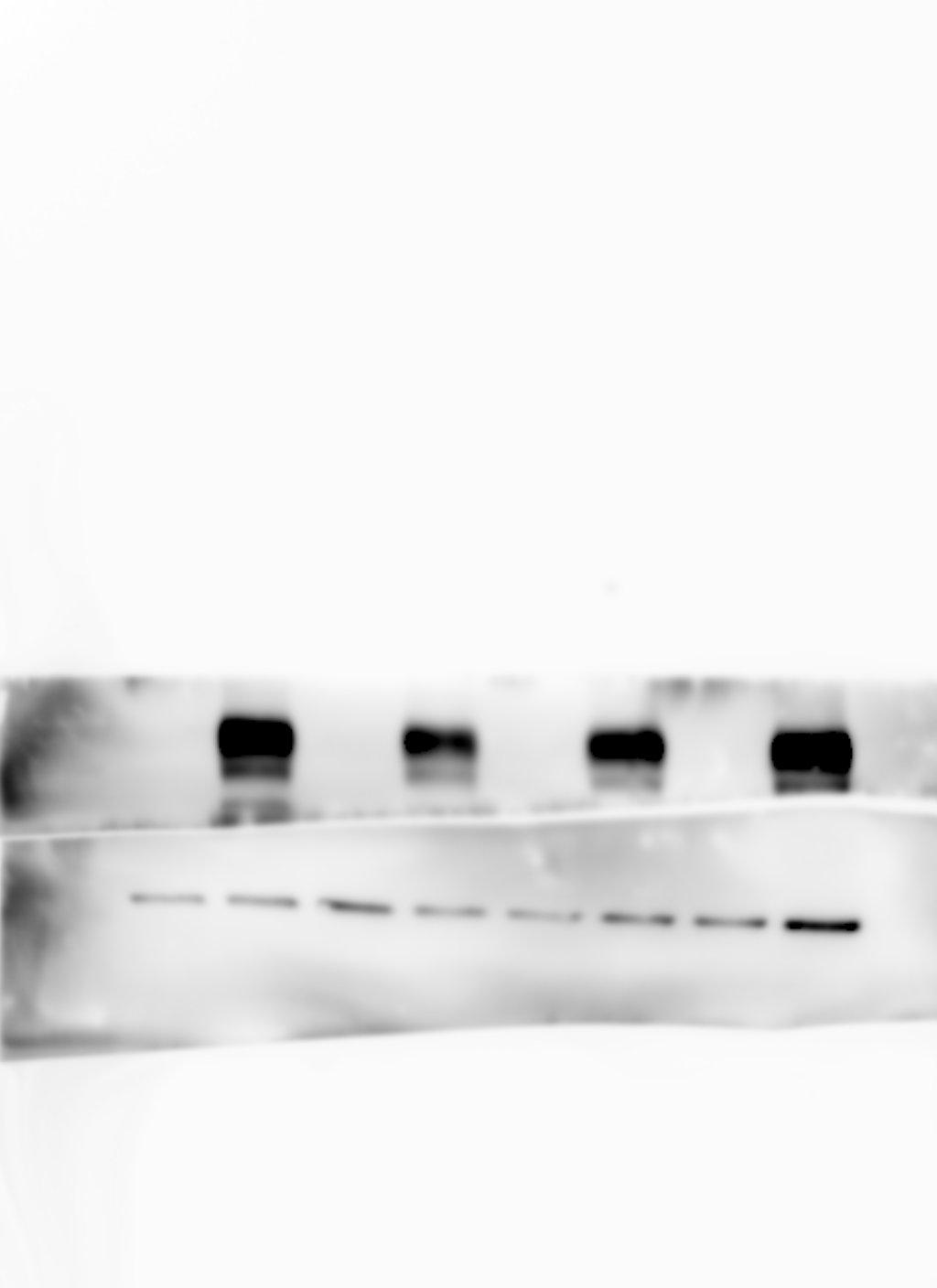

Supplement: Figure 1—source data 1. [file elife-100968-fig1-data1.zip › Figure 1G/GAPDH-unedited gels.jpg]

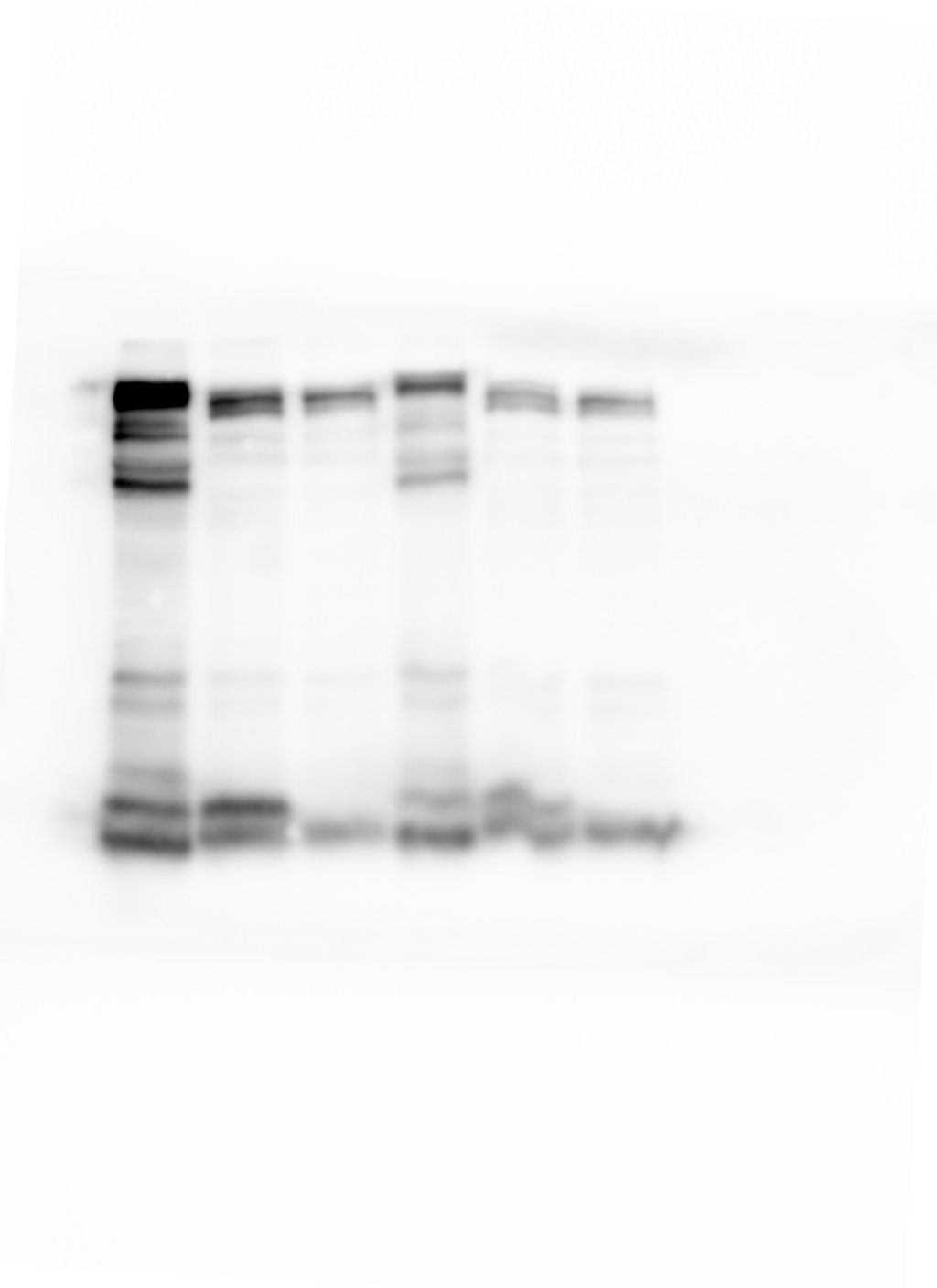

Supplement: Figure 1—source data 1. [file elife-100968-fig1-data1.zip › Figure 1I/CTFs-unedited gels.jpg]

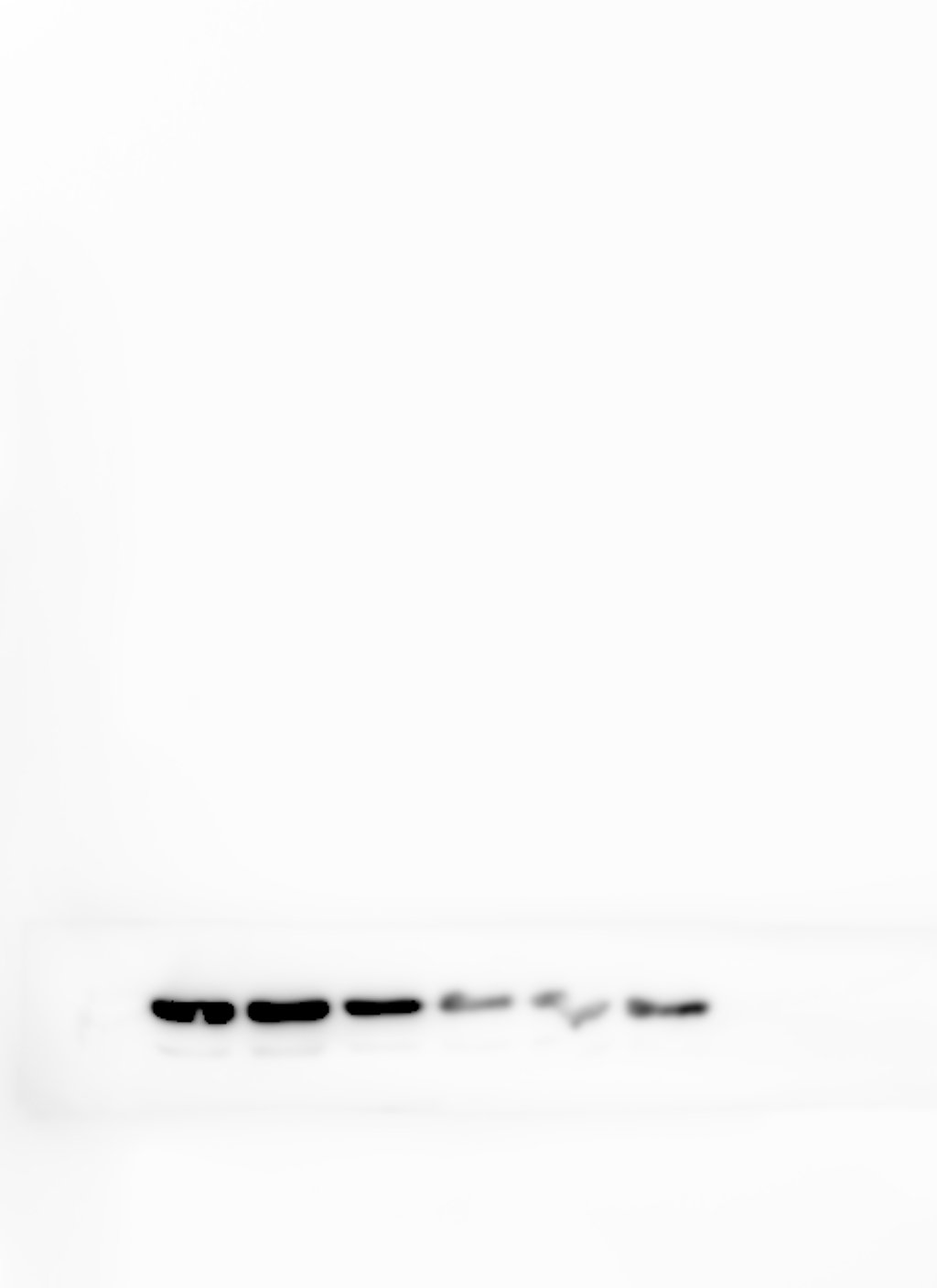

Supplement: Figure 1—source data 1. [file elife-100968-fig1-data1.zip › Figure 1I/GAPDH-unedited gels.jpg]

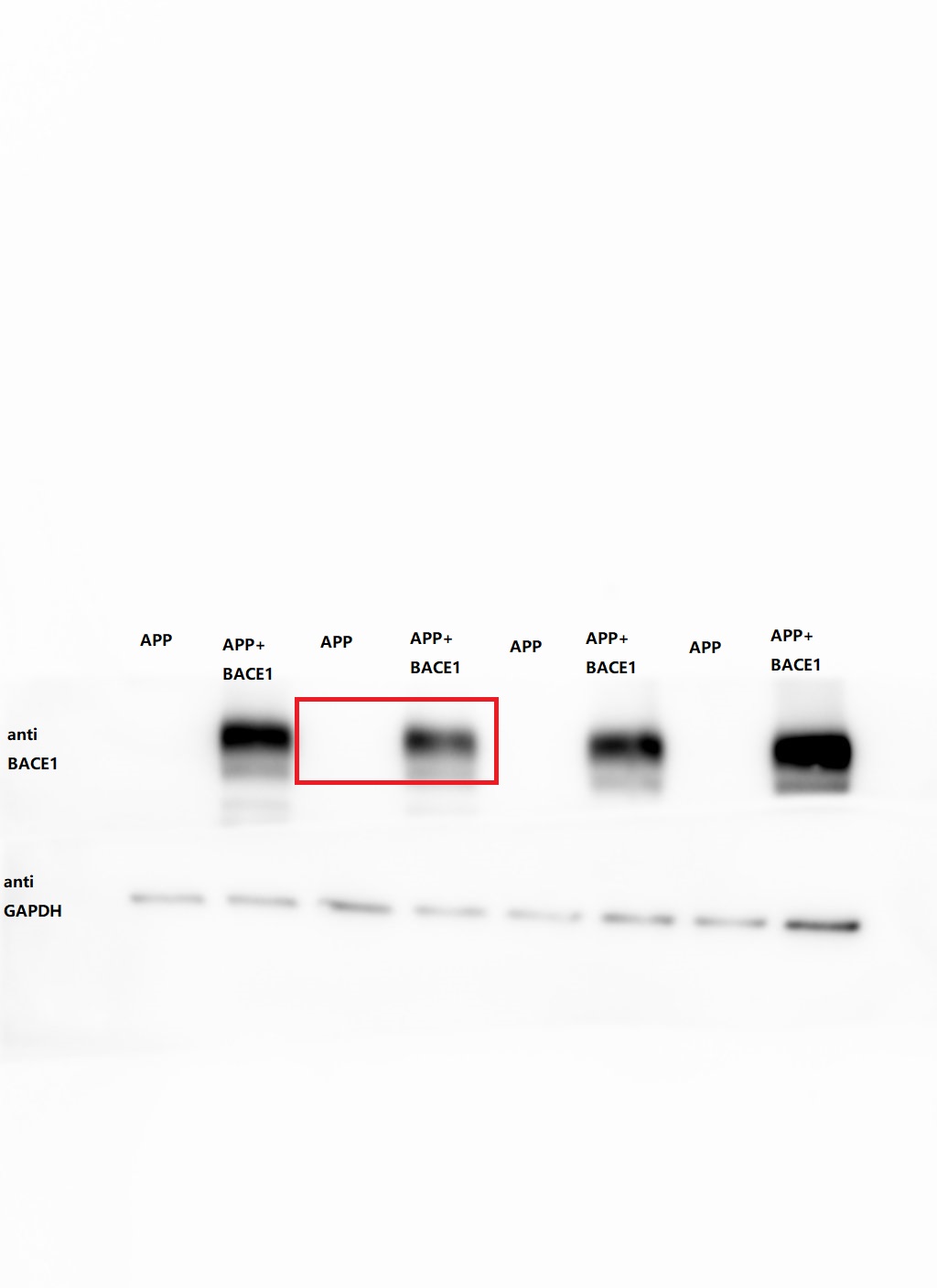

Supplement: Figure 1—source data 2. [file elife-100968-fig1-data2.zip › Figure 1G/BACE1-labelled.jpg]

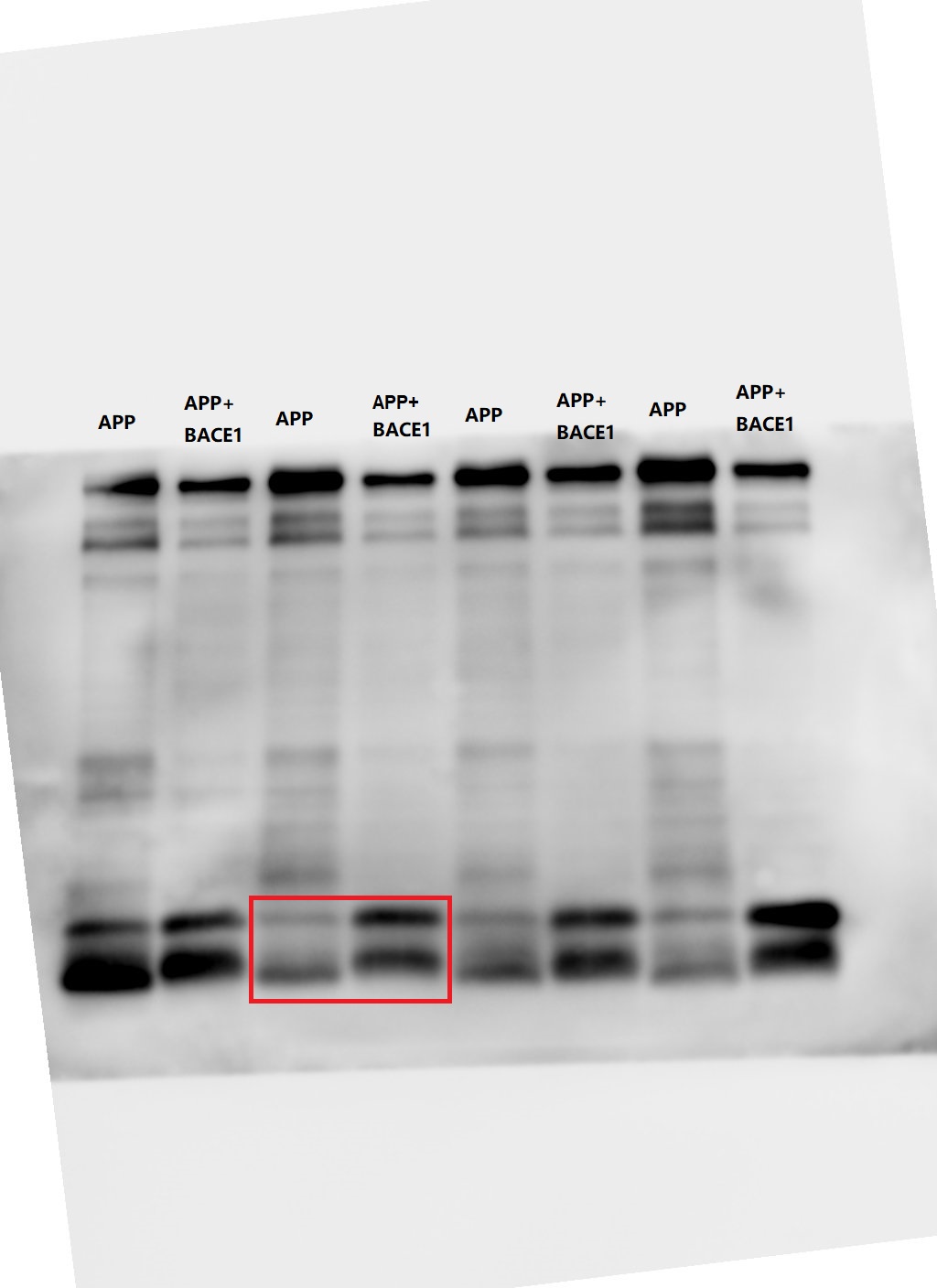

Supplement: Figure 1—source data 2. [file elife-100968-fig1-data2.zip › Figure 1G/CTFs - labelled.jpg]

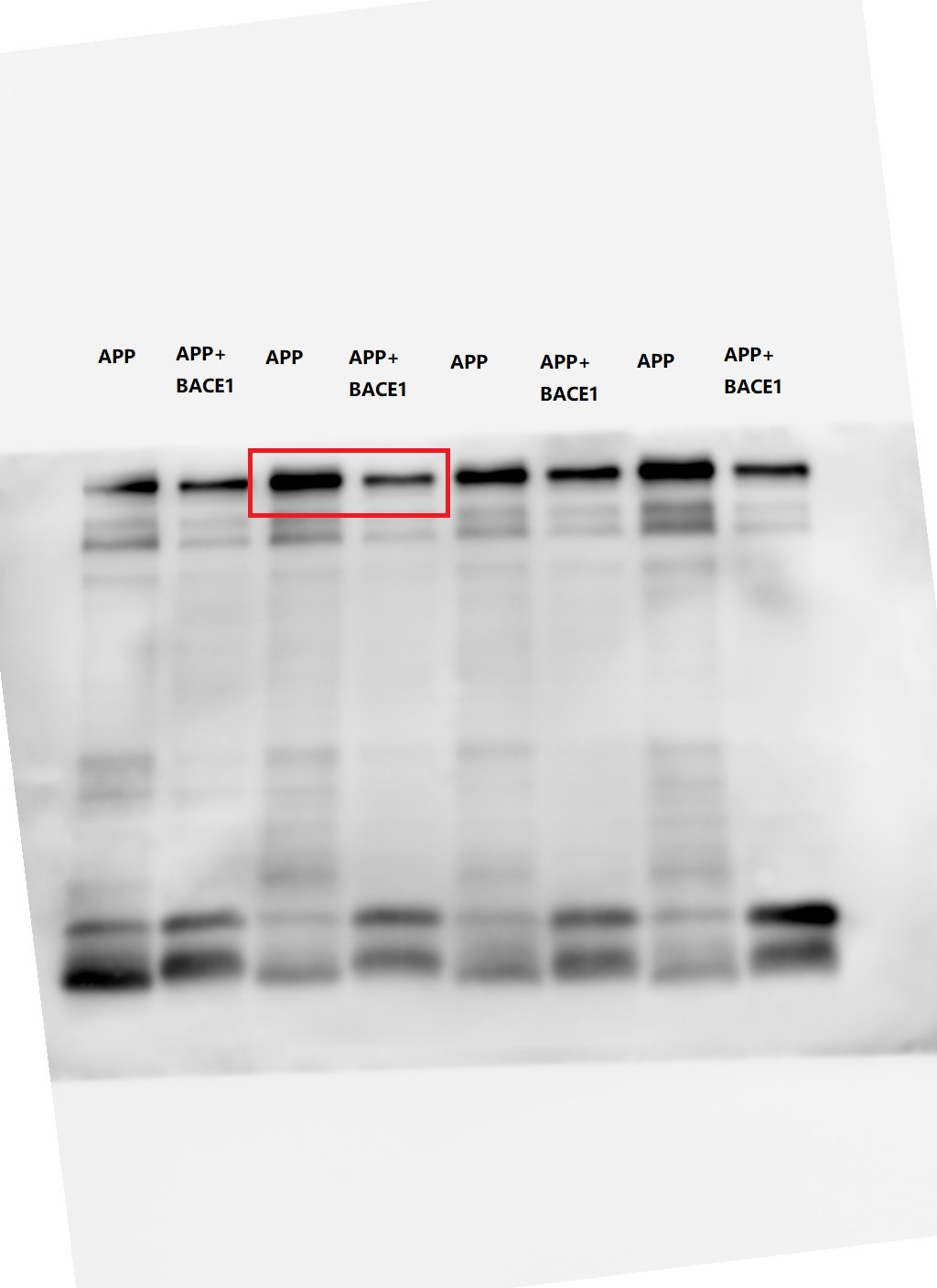

Supplement: Figure 1—source data 2. [file elife-100968-fig1-data2.zip › Figure 1G/FL-APP- labelled.jpg]

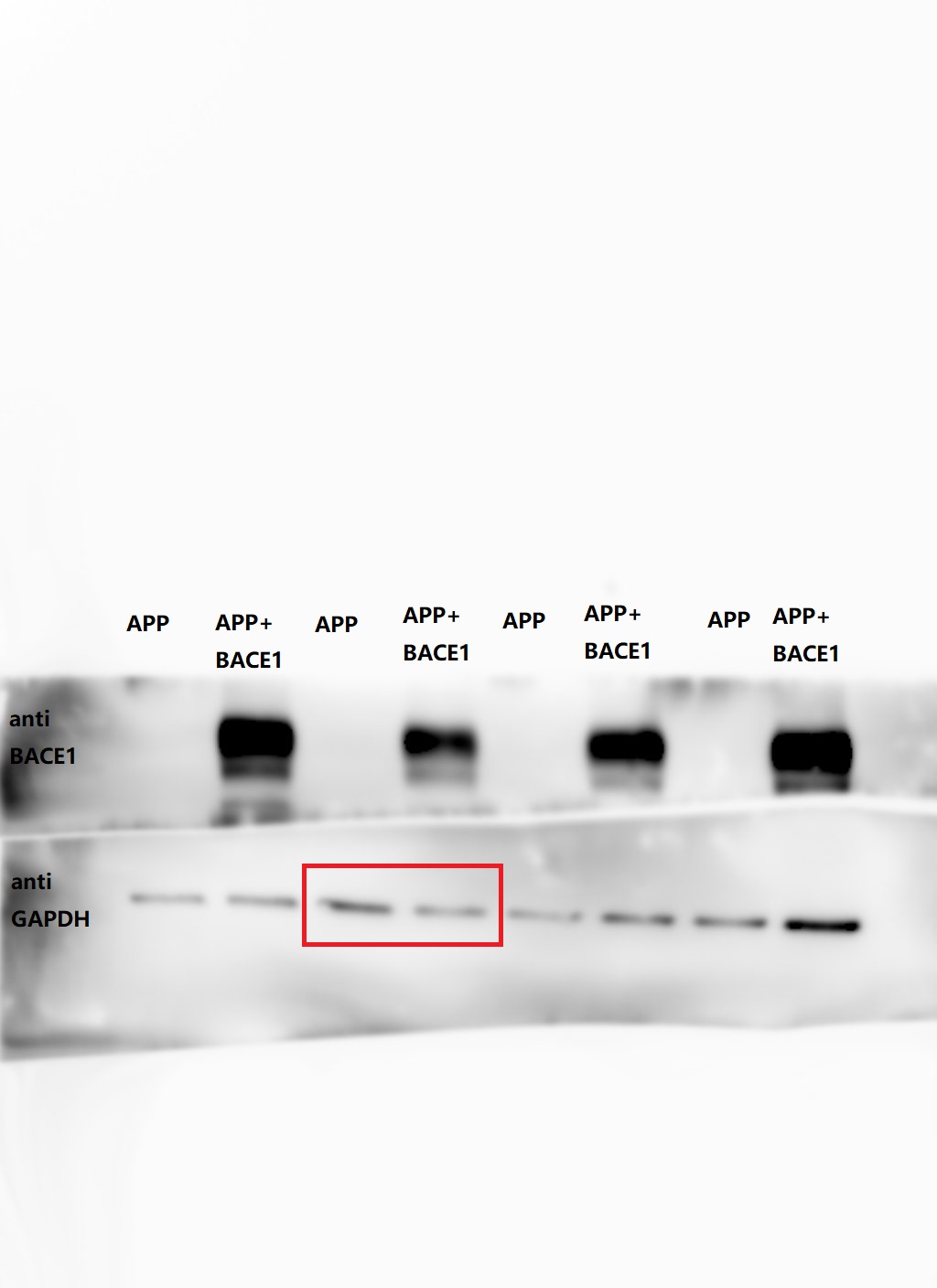

Supplement: Figure 1—source data 2. [file elife-100968-fig1-data2.zip › Figure 1G/GAPDH-labelled.jpg]

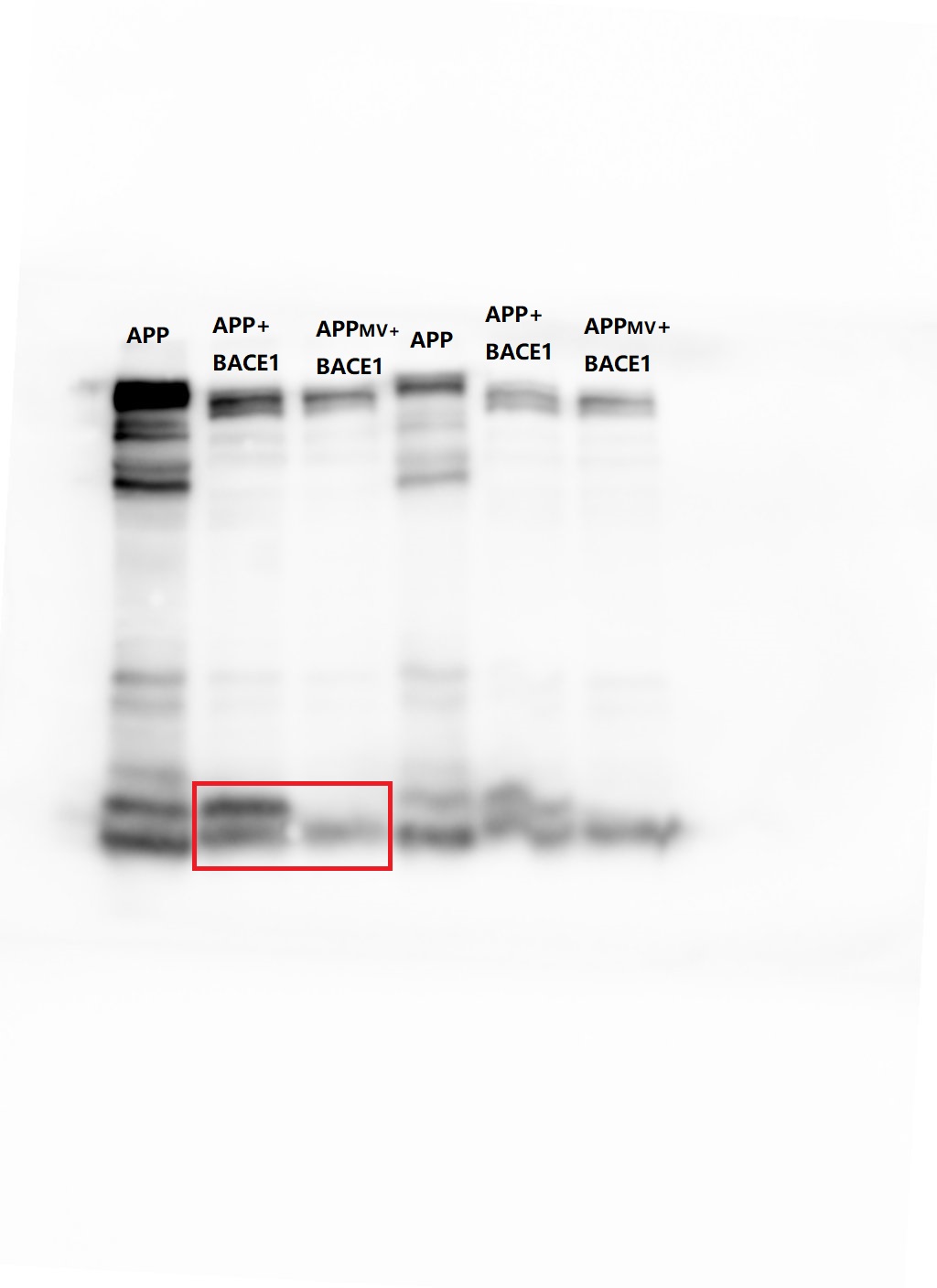

Supplement: Figure 1—source data 2. [file elife-100968-fig1-data2.zip › Figure 1I/CTFs-labelled.jpg]

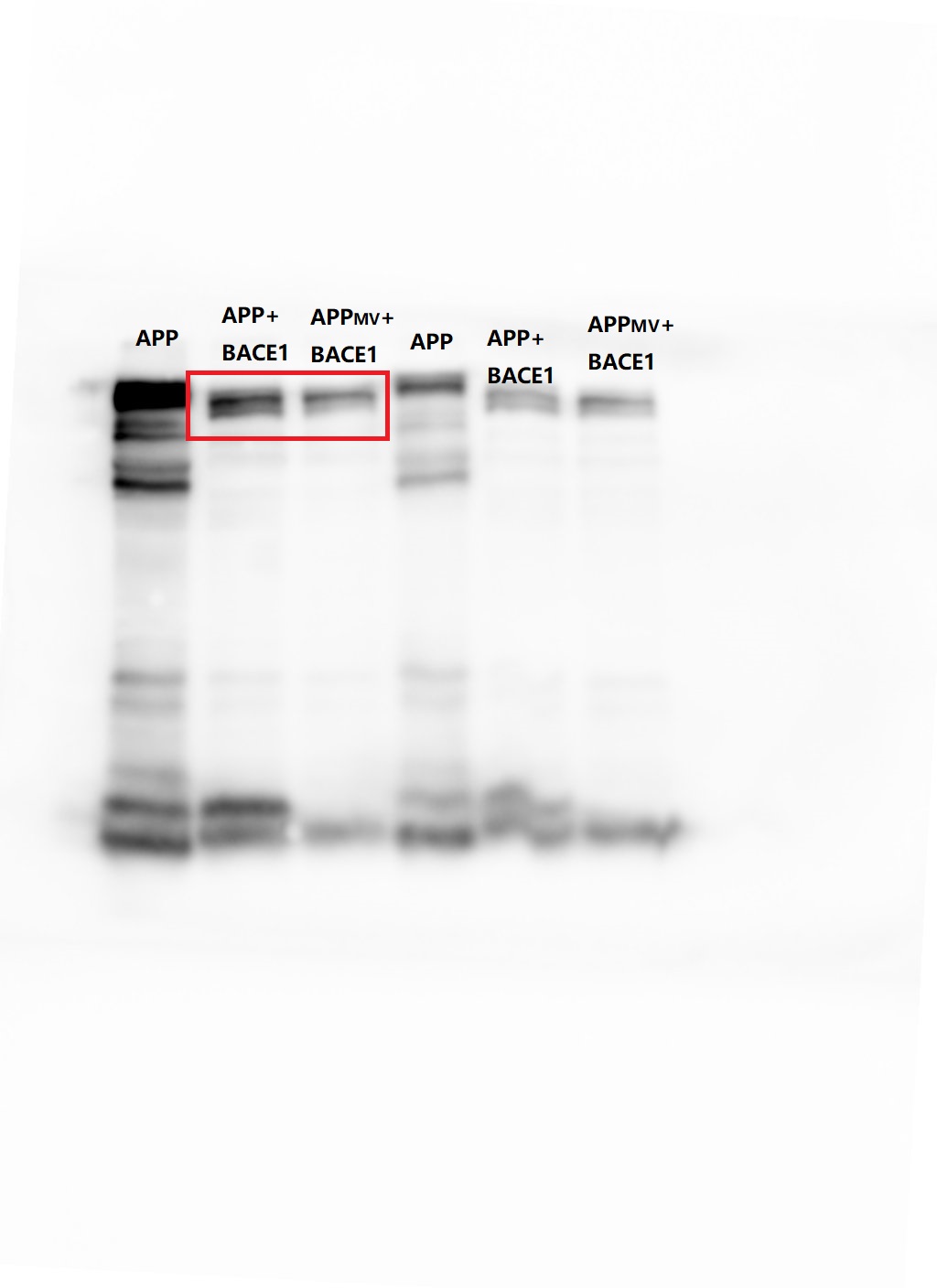

Supplement: Figure 1—source data 2. [file elife-100968-fig1-data2.zip › Figure 1I/FL-APP-labelled.jpg]

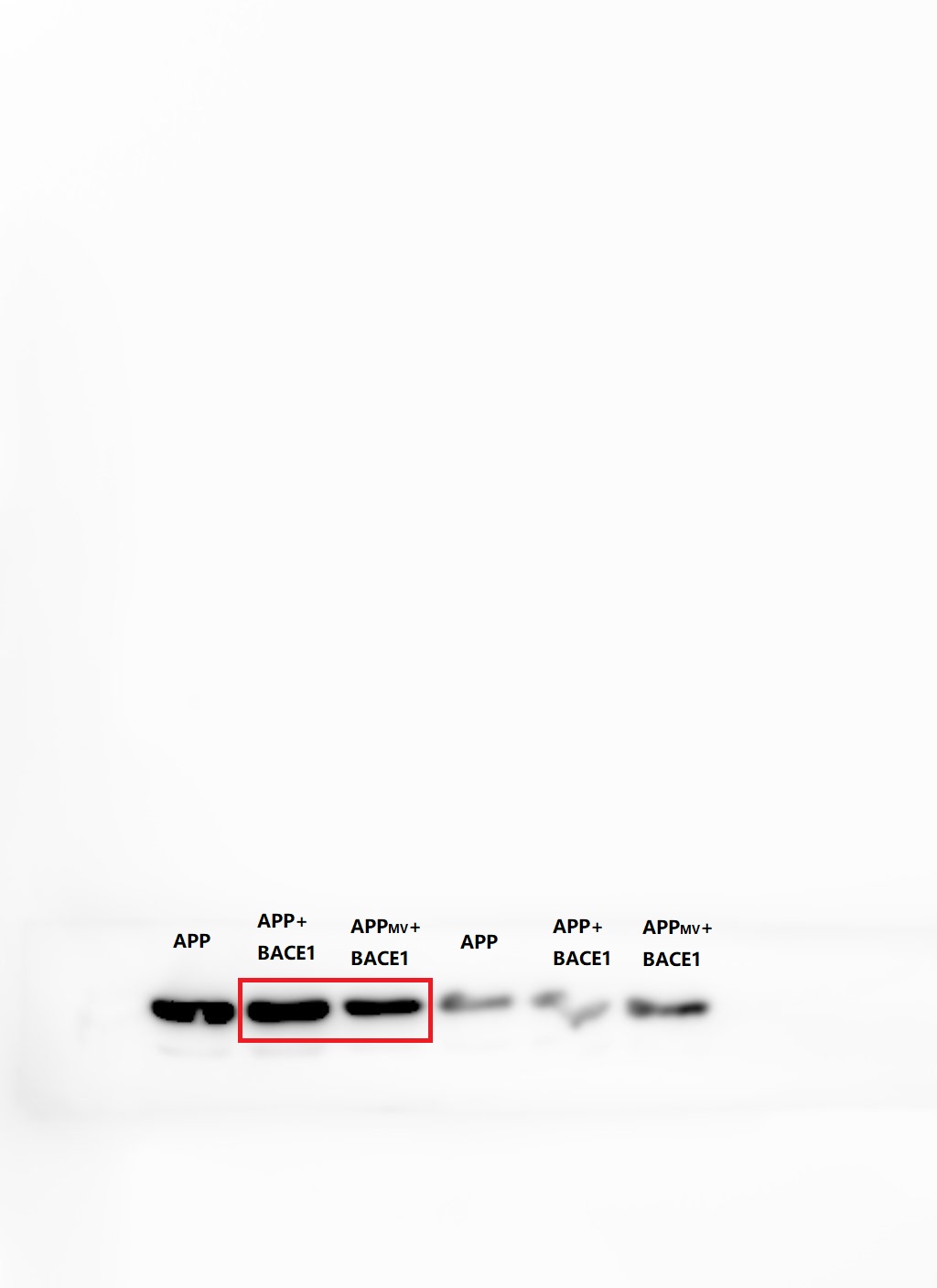

Supplement: Figure 1—source data 2. [file elife-100968-fig1-data2.zip › Figure 1I/GAPDH-labelled.jpg]

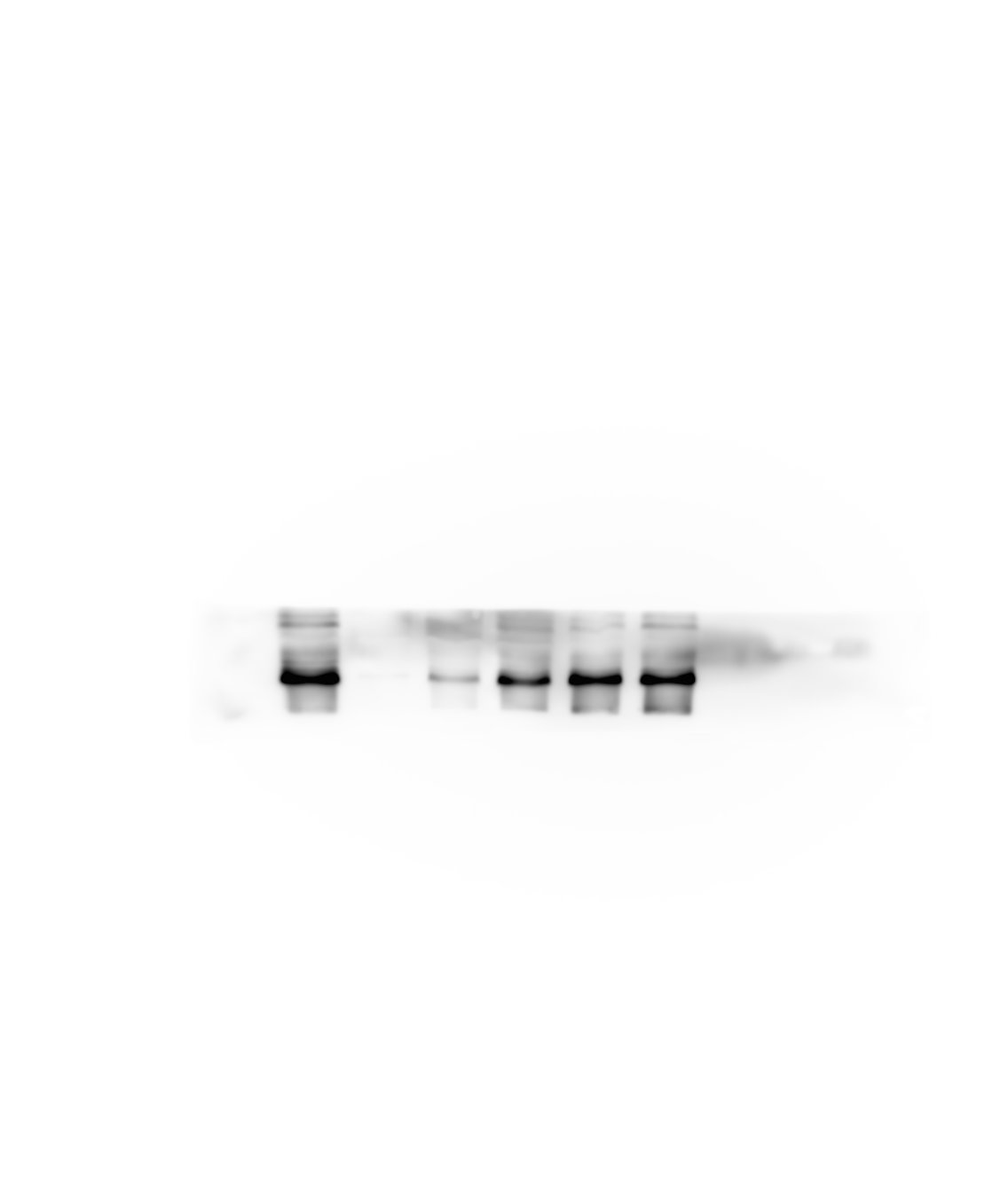

Supplement: Figure 1—figure supplement 1—source data 1. [file elife-100968-fig1-figsupp1-data1.zip › Figure 1-figure supplement 1C/BACE1-unedited gels.jpg]

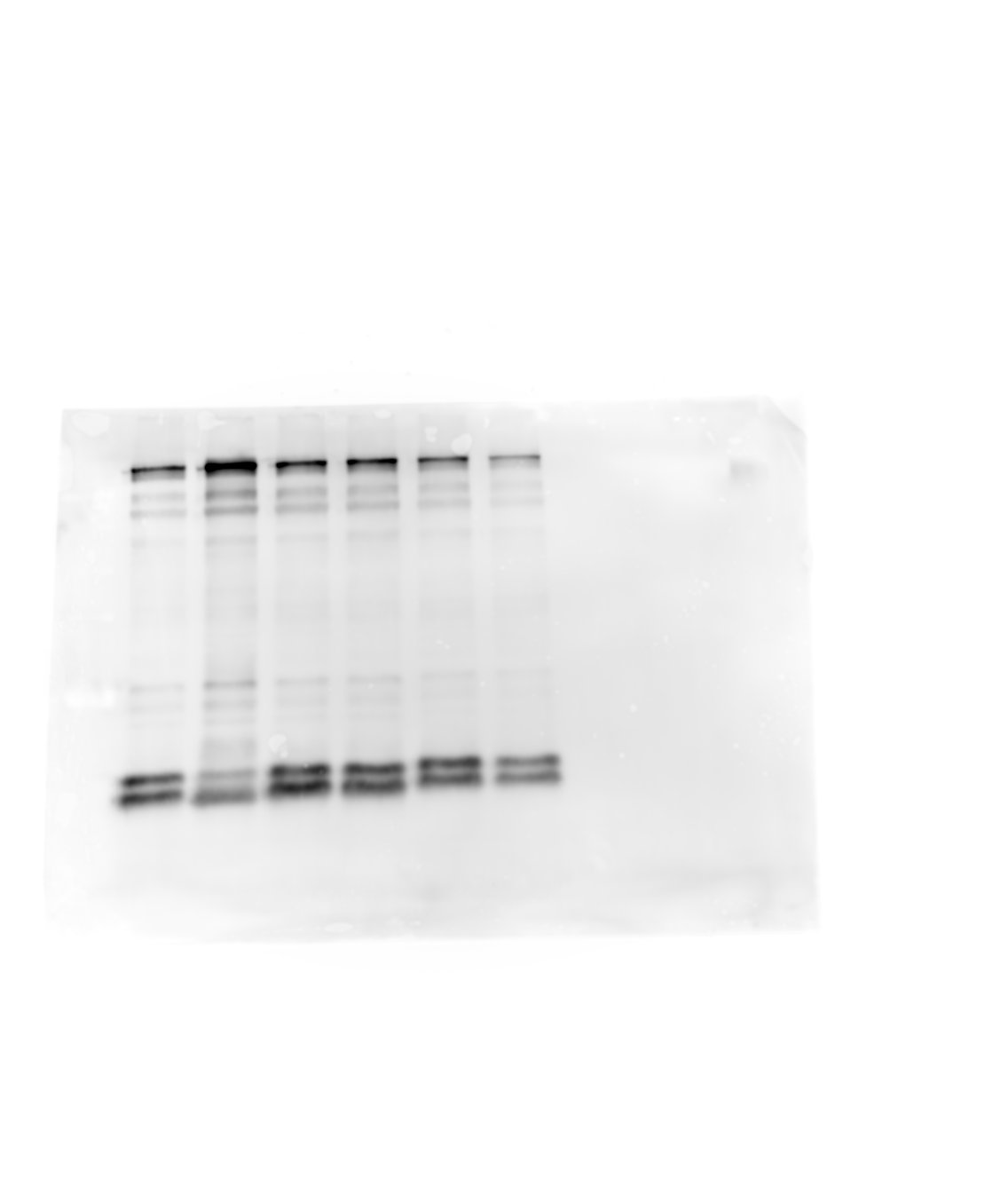

Supplement: Figure 1—figure supplement 1—source data 1. [file elife-100968-fig1-figsupp1-data1.zip › Figure 1-figure supplement 1C/CTFs-unedited gels.jpg]

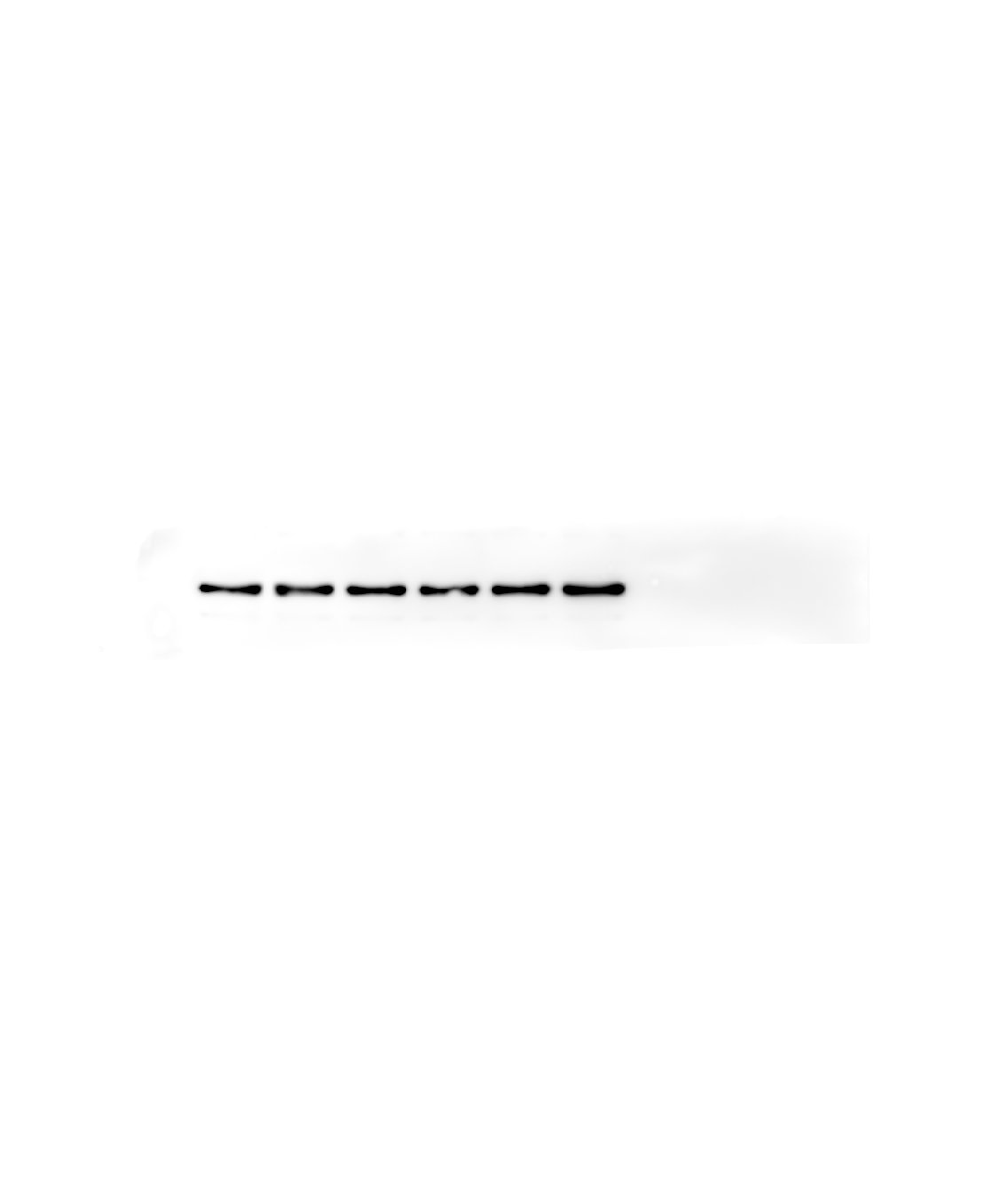

Supplement: Figure 1—figure supplement 1—source data 1. [file elife-100968-fig1-figsupp1-data1.zip › Figure 1-figure supplement 1C/GAPDH-unedited gels.jpg]

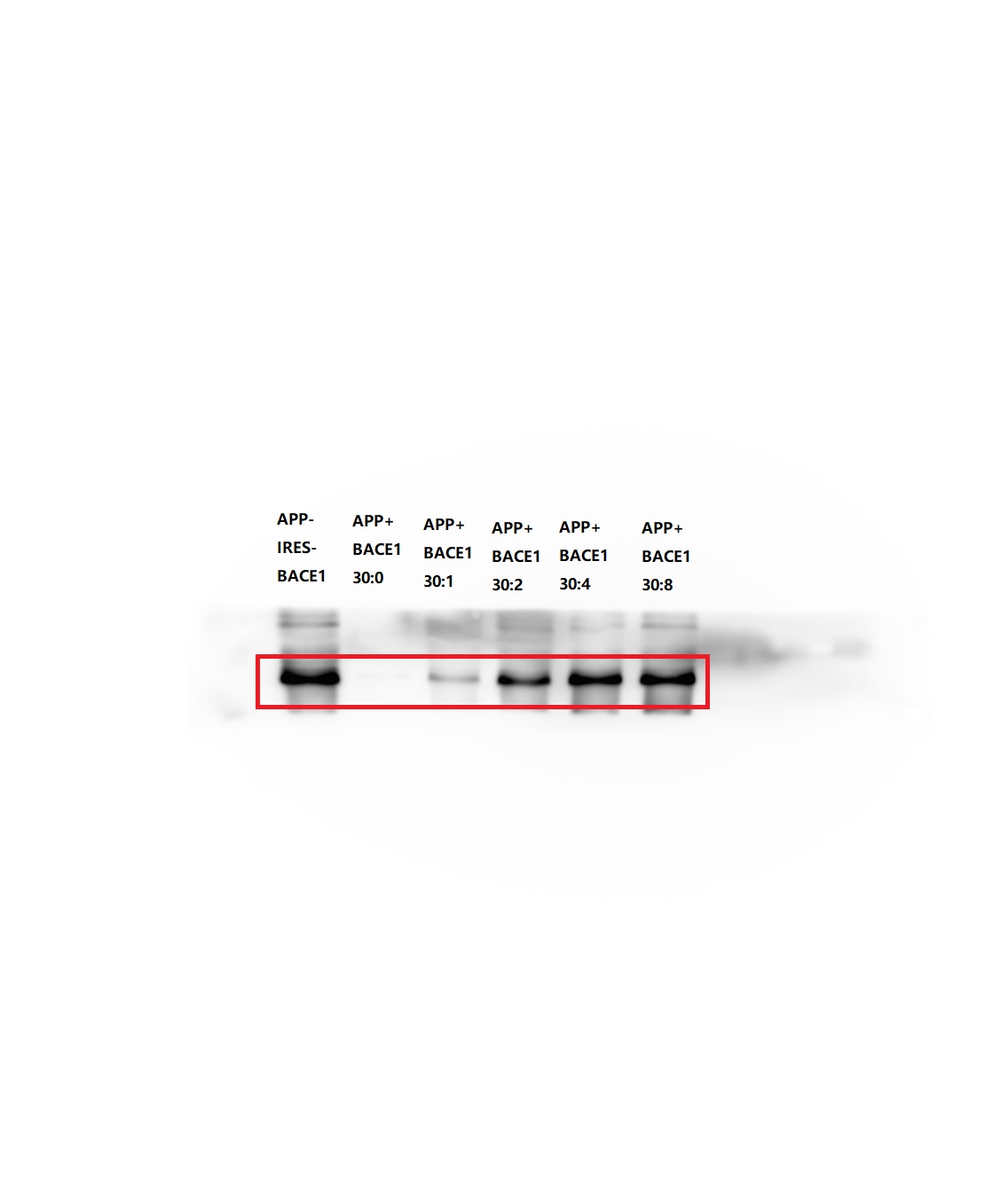

Supplement: Figure 1—figure supplement 1—source data 2. [file elife-100968-fig1-figsupp1-data2.zip › Figure 1-figure supplement 1C/BACE1-labelled.jpg]

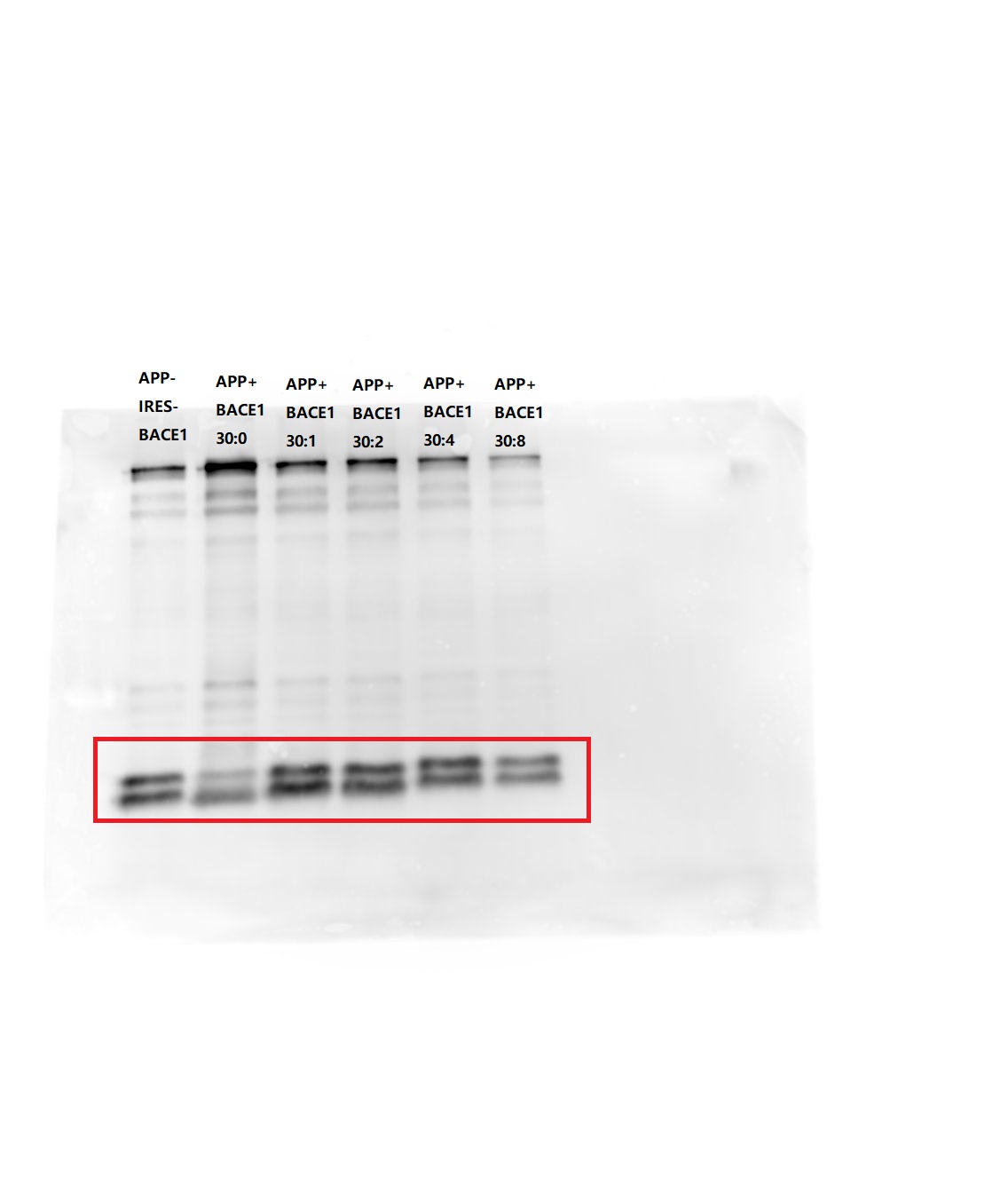

Supplement: Figure 1—figure supplement 1—source data 2. [file elife-100968-fig1-figsupp1-data2.zip › Figure 1-figure supplement 1C/CTFs-labelled.jpg]

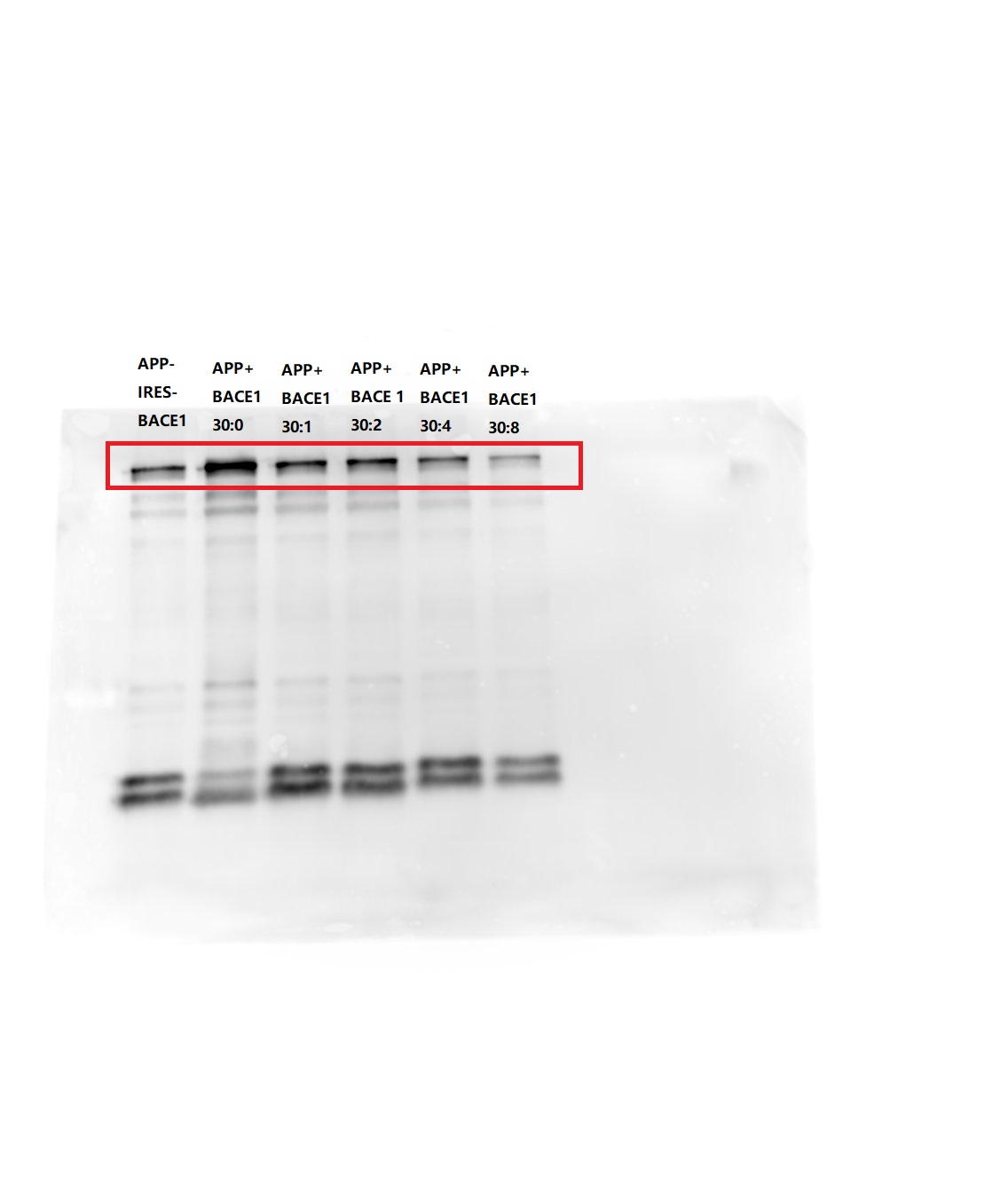

Supplement: Figure 1—figure supplement 1—source data 2. [file elife-100968-fig1-figsupp1-data2.zip › Figure 1-figure supplement 1C/FL-APP-labelled.jpg]

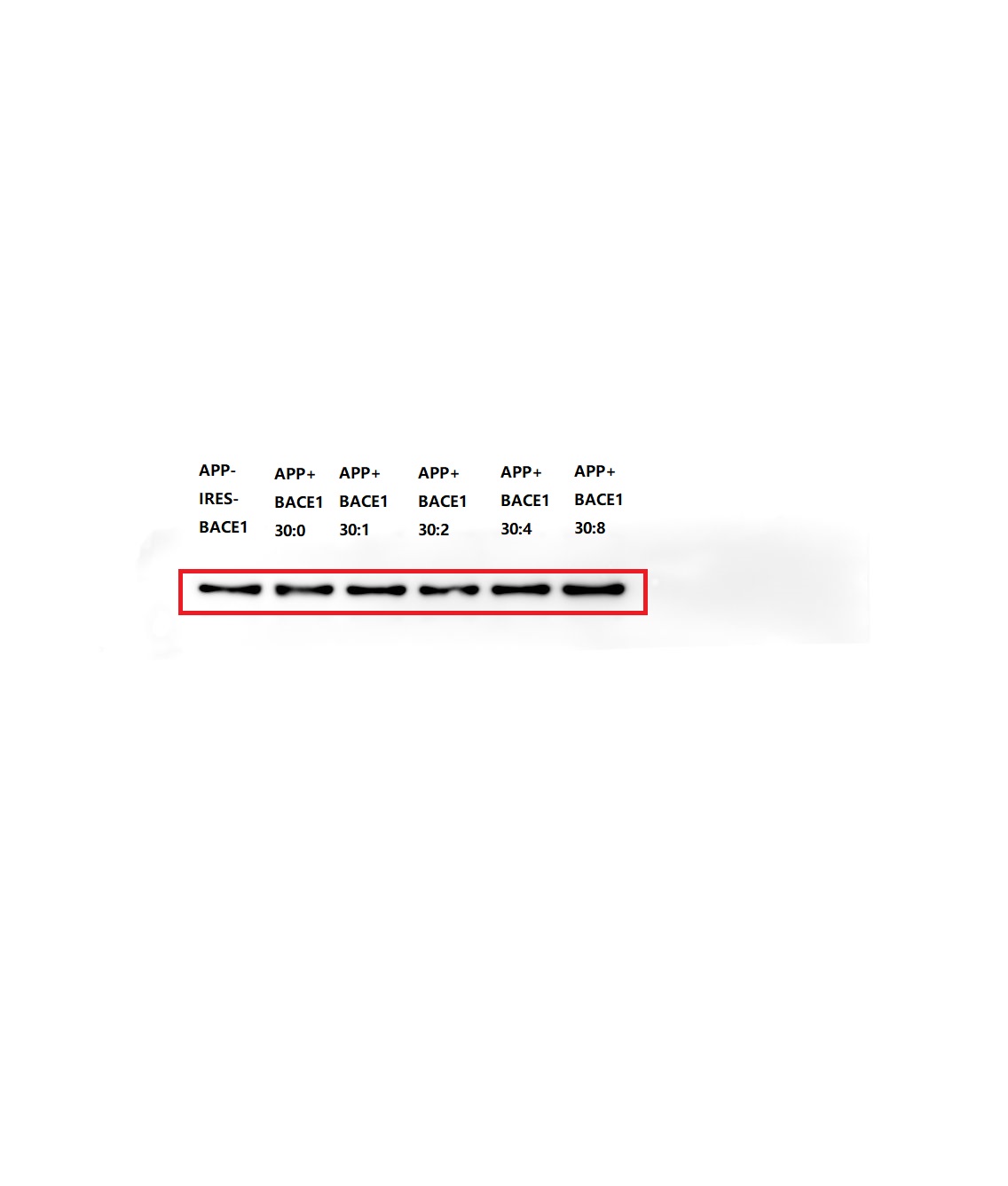

Supplement: Figure 1—figure supplement 1—source data 2. [file elife-100968-fig1-figsupp1-data2.zip › Figure 1-figure supplement 1C/GAPDH-labelled.jpg]

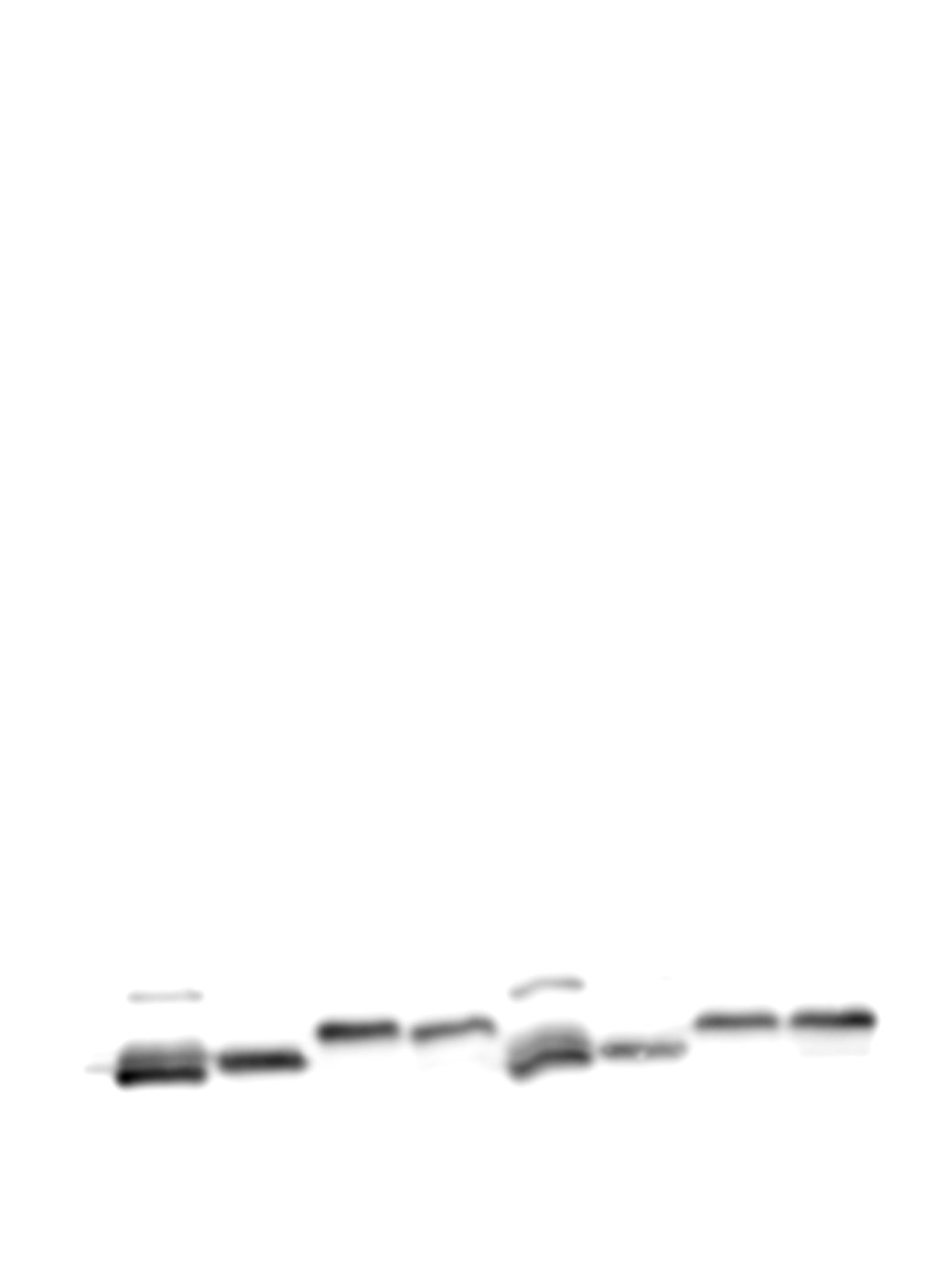

Supplement: Figure 2—source data 1. [file elife-100968-fig2-data1.zip › Figure 2C/CTFs-unedited gels.jpg]

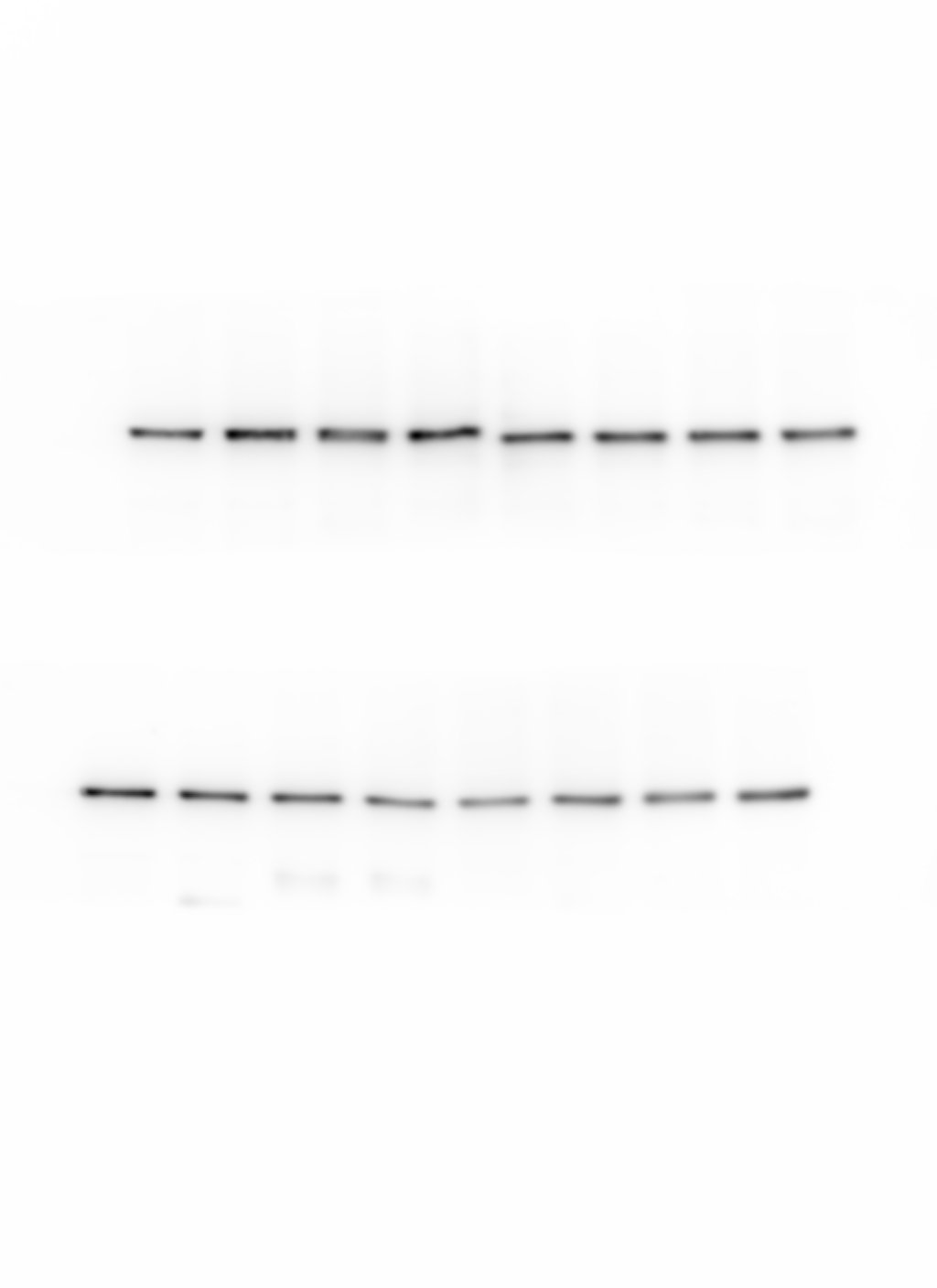

Supplement: Figure 2—source data 1. [file elife-100968-fig2-data1.zip › Figure 2C/GAPDH-unedited gels.jpg]

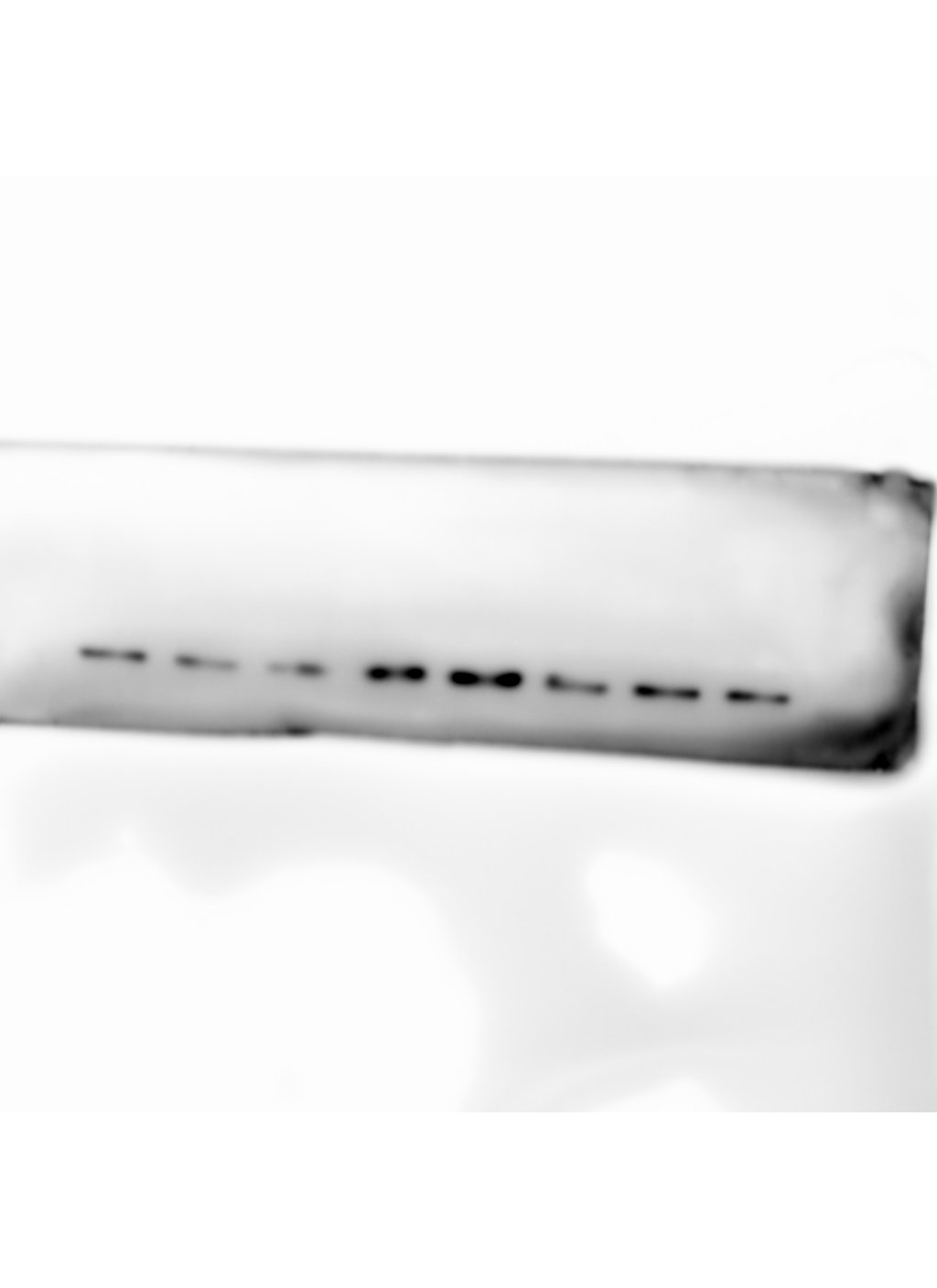

Supplement: Figure 2—source data 1. [file elife-100968-fig2-data1.zip › Figure 2D/GAPDH-unedited gels.jpg]

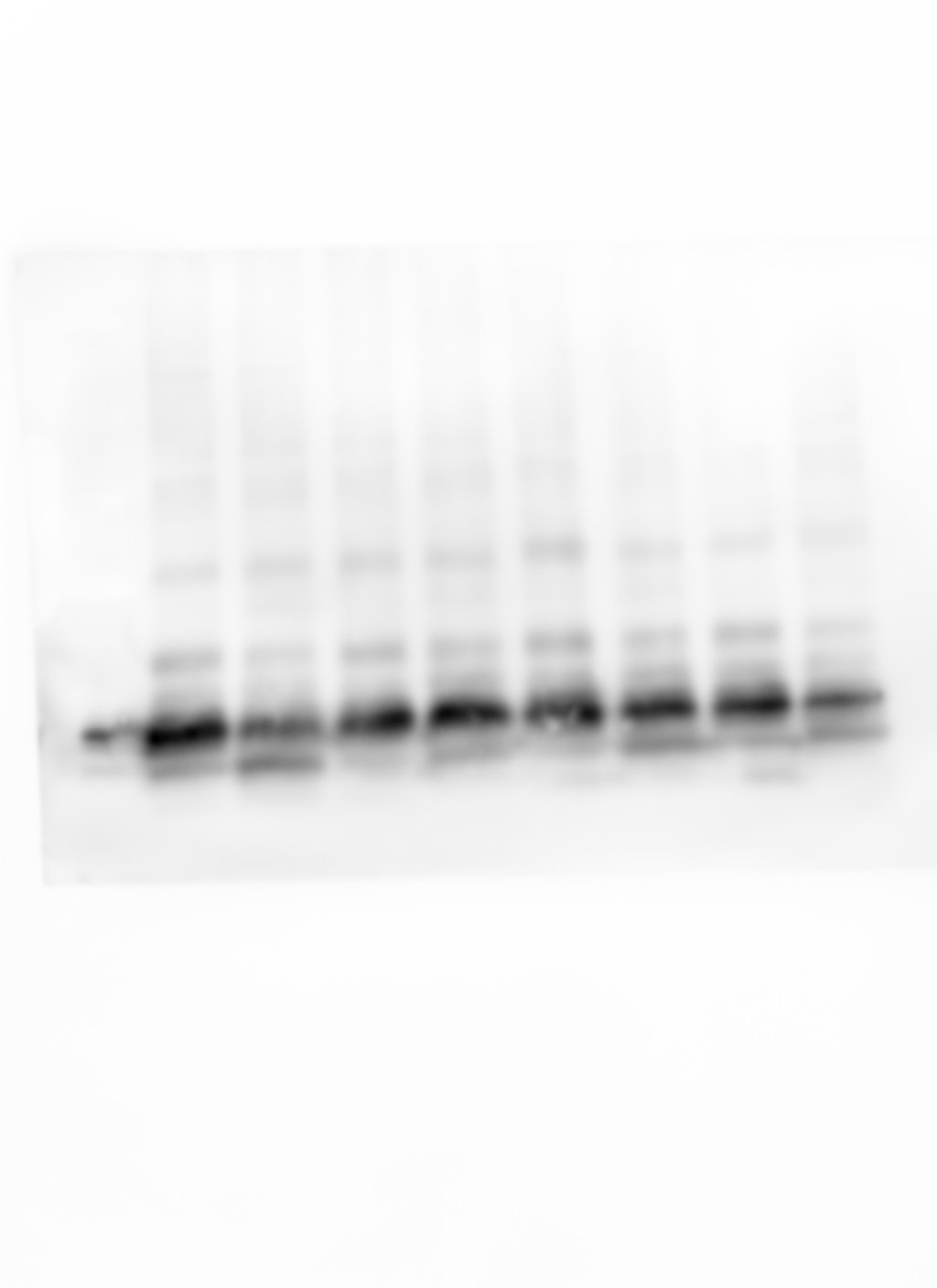

Supplement: Figure 2—source data 1. [file elife-100968-fig2-data1.zip › Figure 2D/a┬-CTF-unedited gels.jpg]

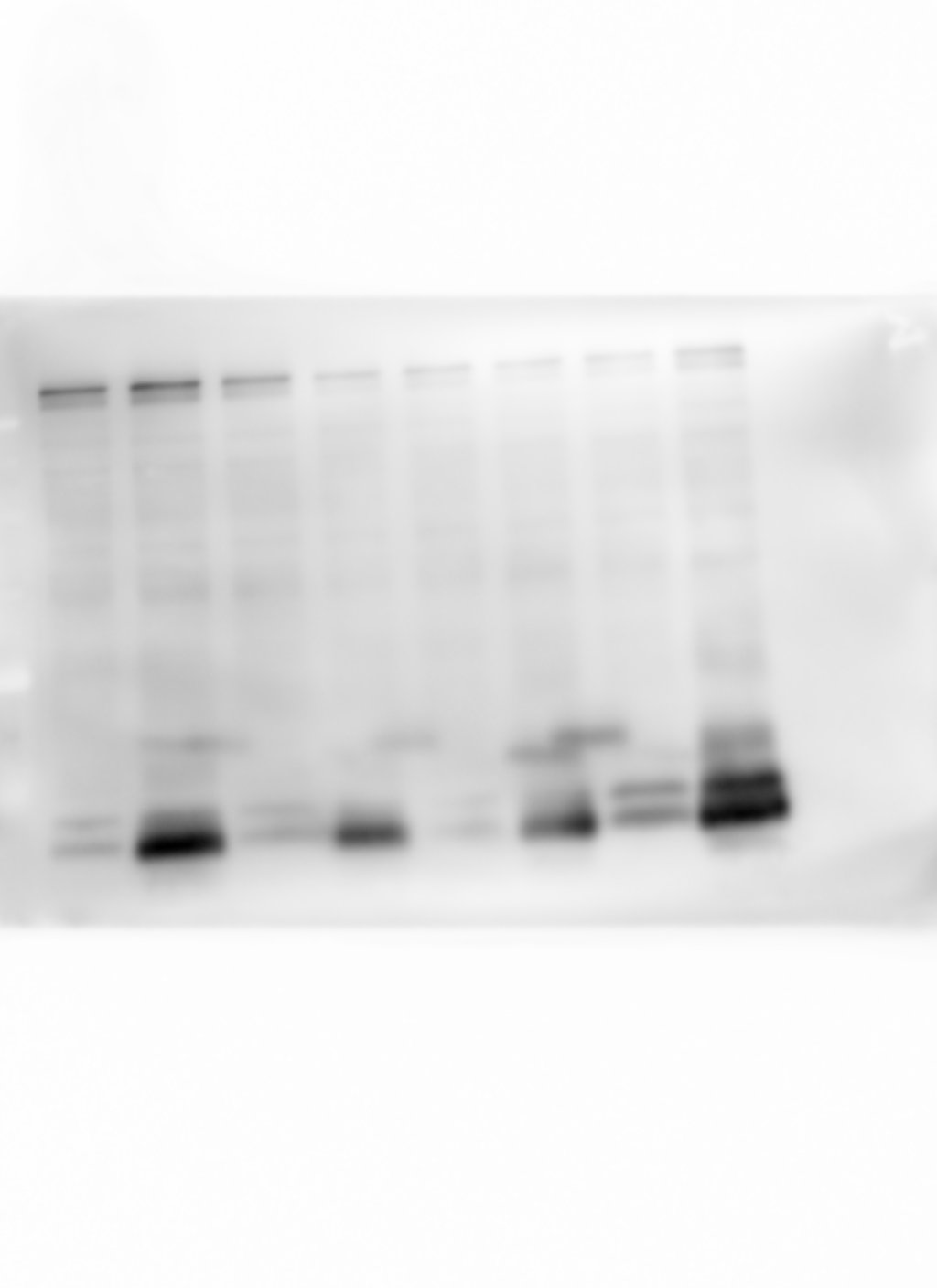

Supplement: Figure 2—source data 1. [file elife-100968-fig2-data1.zip › Figure 2J/CTFs-unedited gels.jpg]

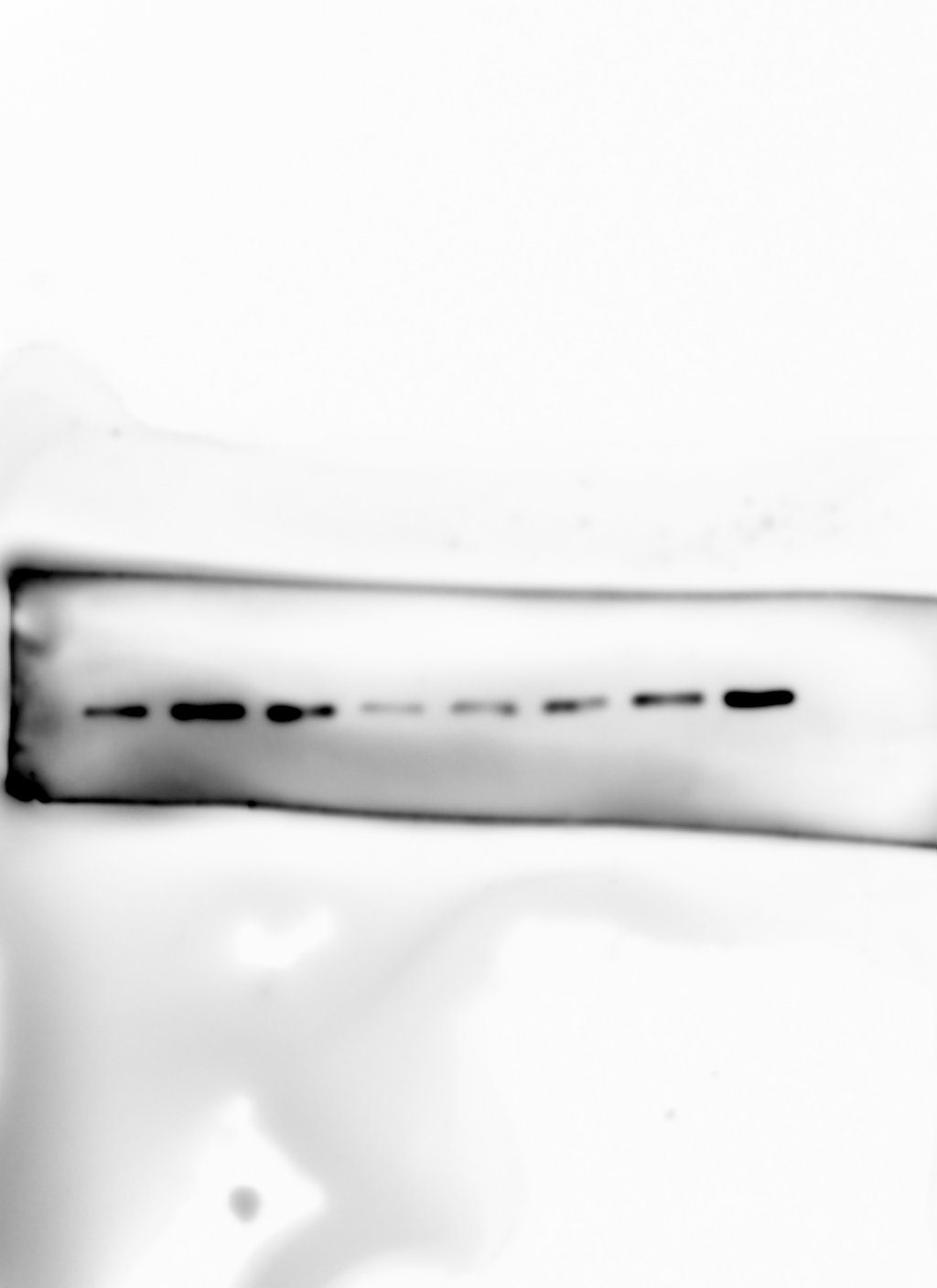

Supplement: Figure 2—source data 1. [file elife-100968-fig2-data1.zip › Figure 2J/GAPDH-unedited gels.jpg]

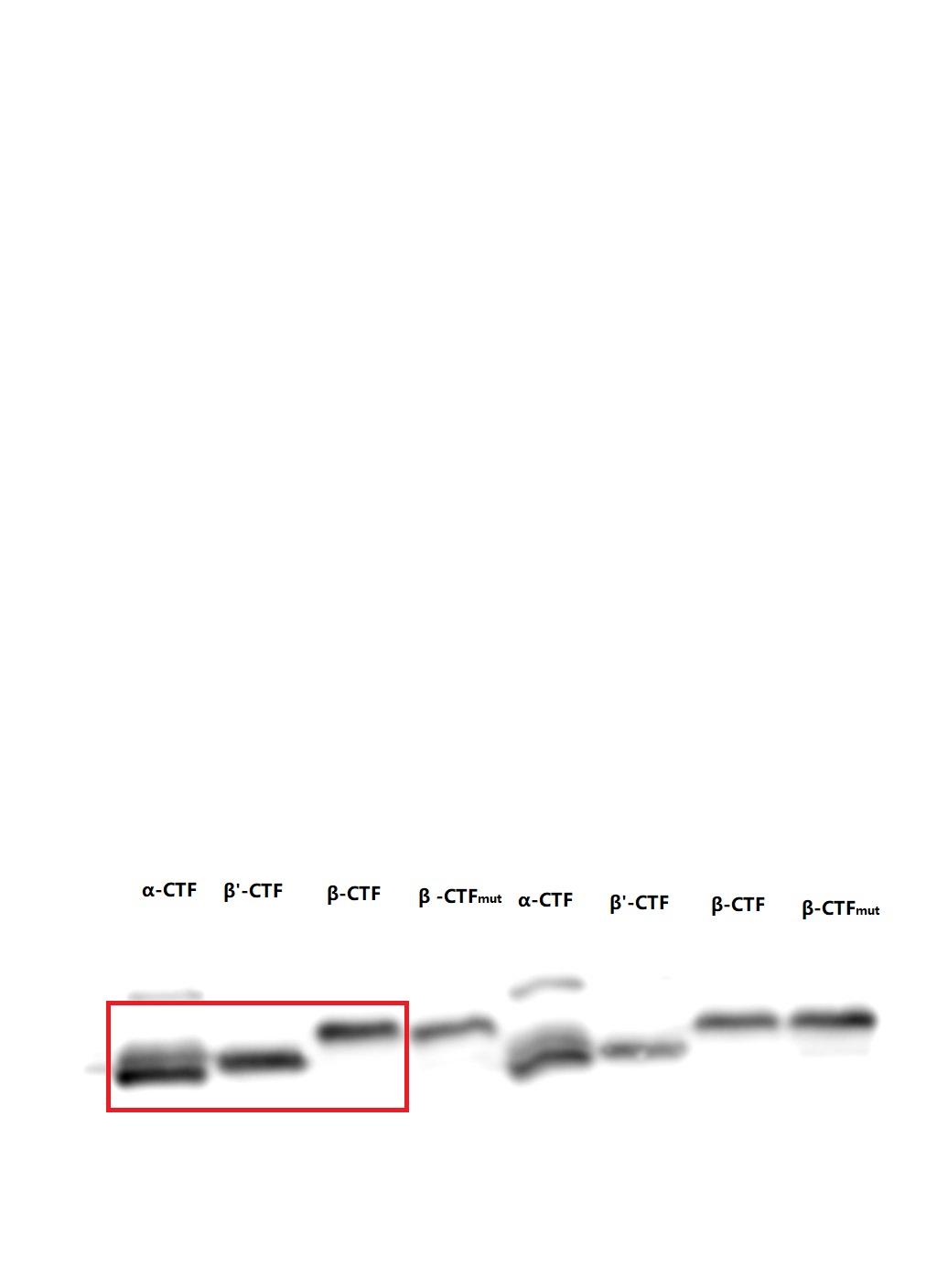

Supplement: Figure 2—source data 2. [file elife-100968-fig2-data2.zip › Figure 2C/CTFs-labelled.jpg]

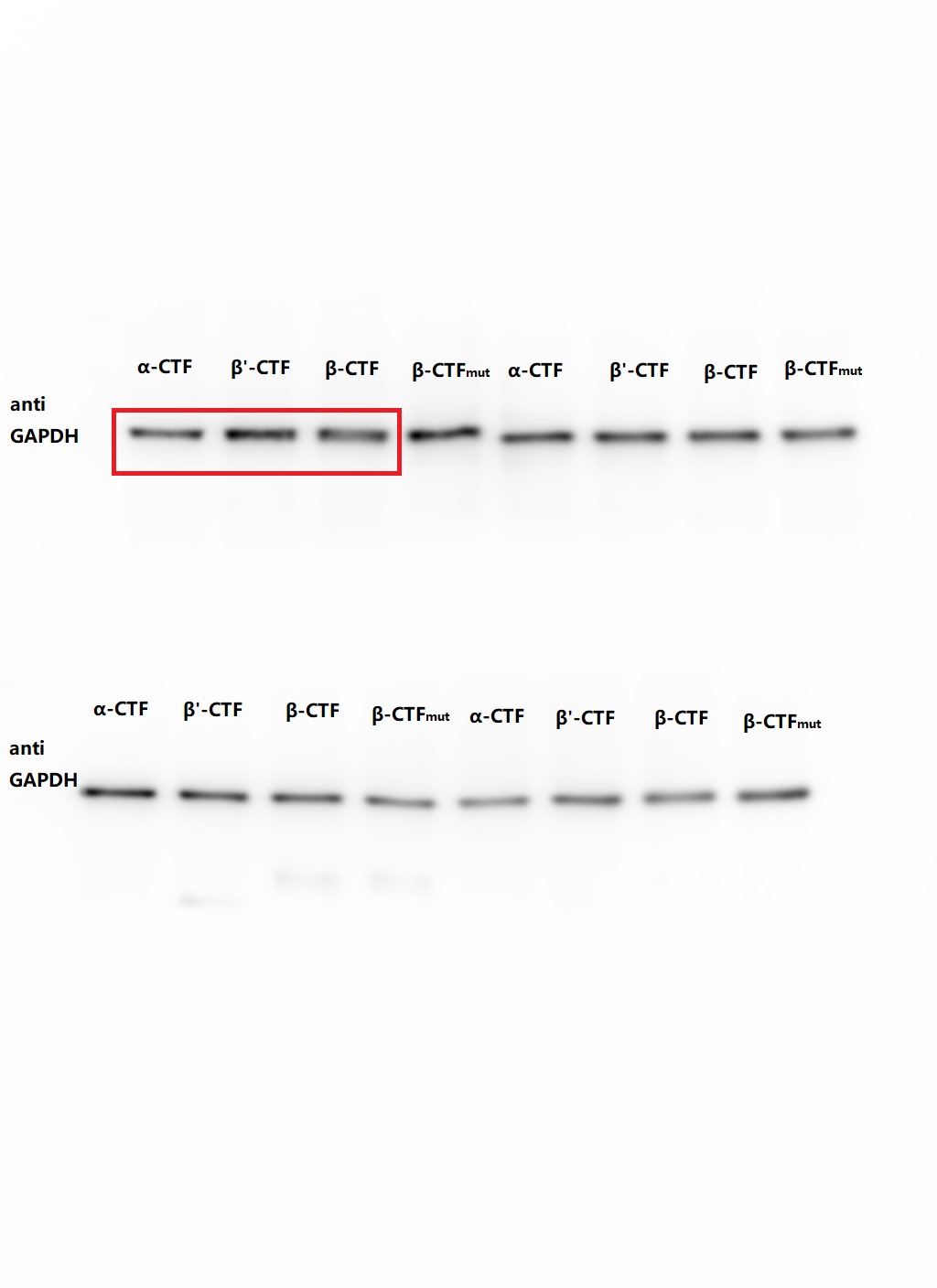

Supplement: Figure 2—source data 2. [file elife-100968-fig2-data2.zip › Figure 2C/GAPDH-labelled.jpg]

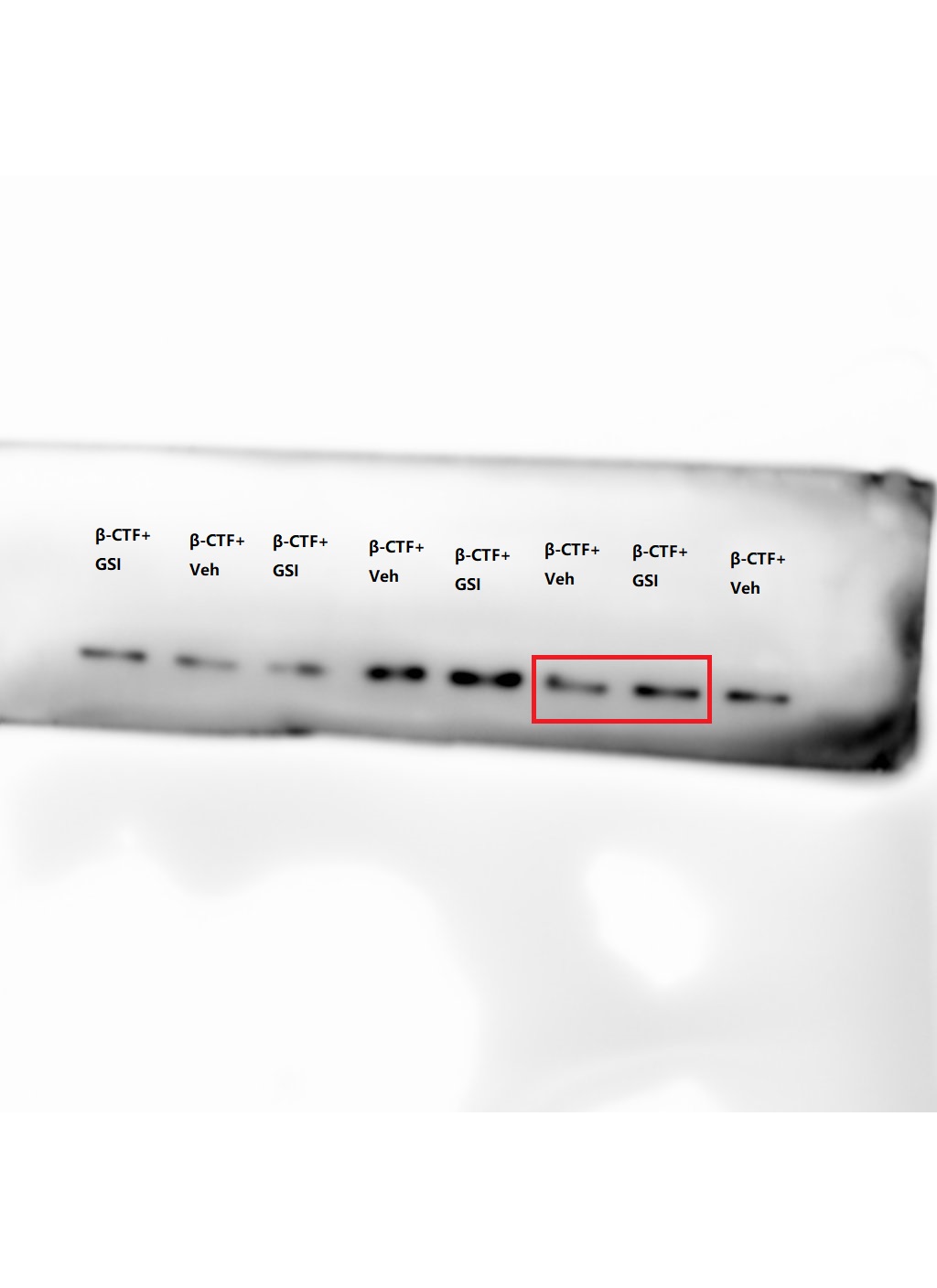

Supplement: Figure 2—source data 2. [file elife-100968-fig2-data2.zip › Figure 2D/GAPDH-labelled.jpg]

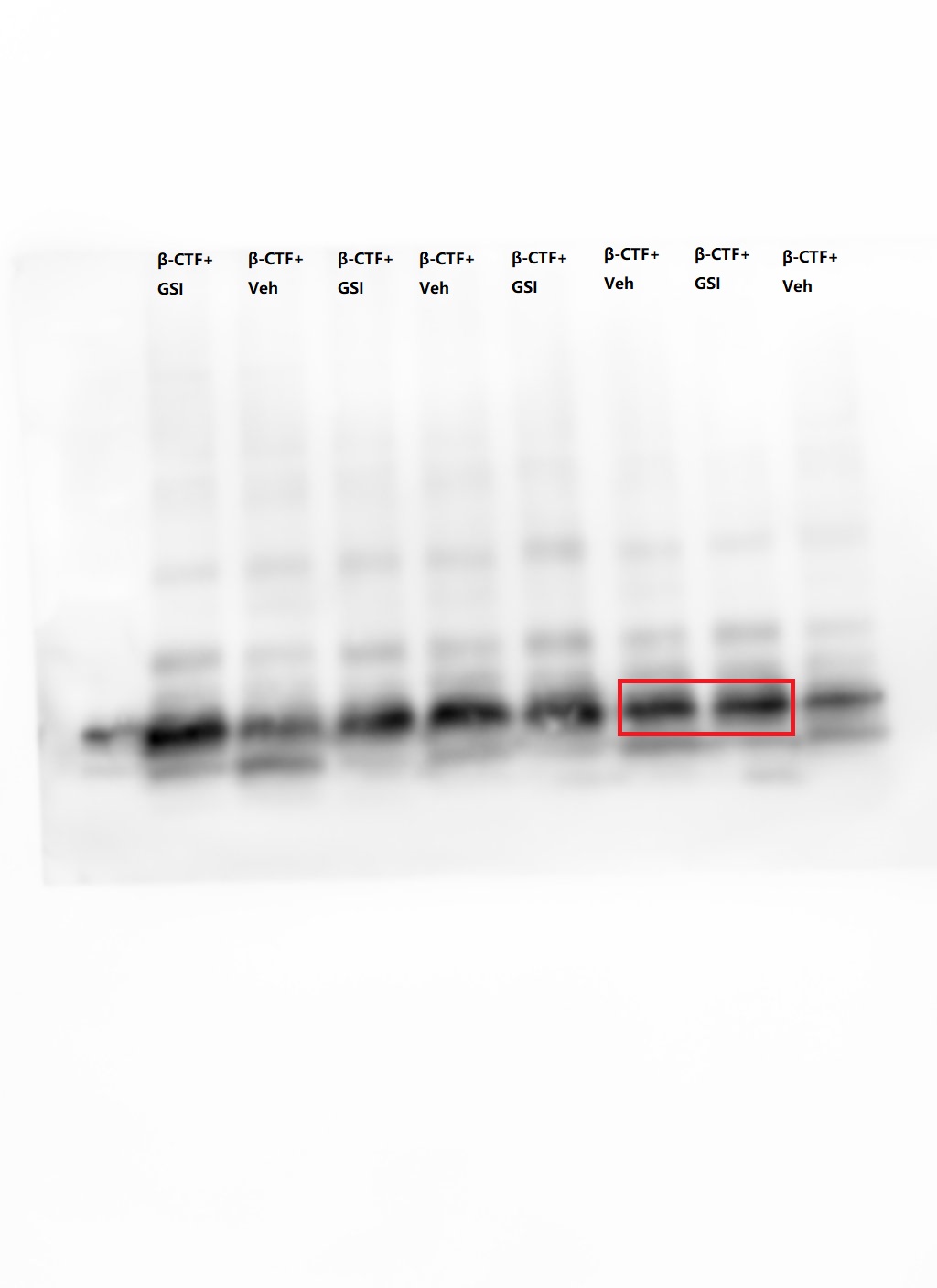

Supplement: Figure 2—source data 2. [file elife-100968-fig2-data2.zip › Figure 2D/a┬-CTF-labelled.jpg]

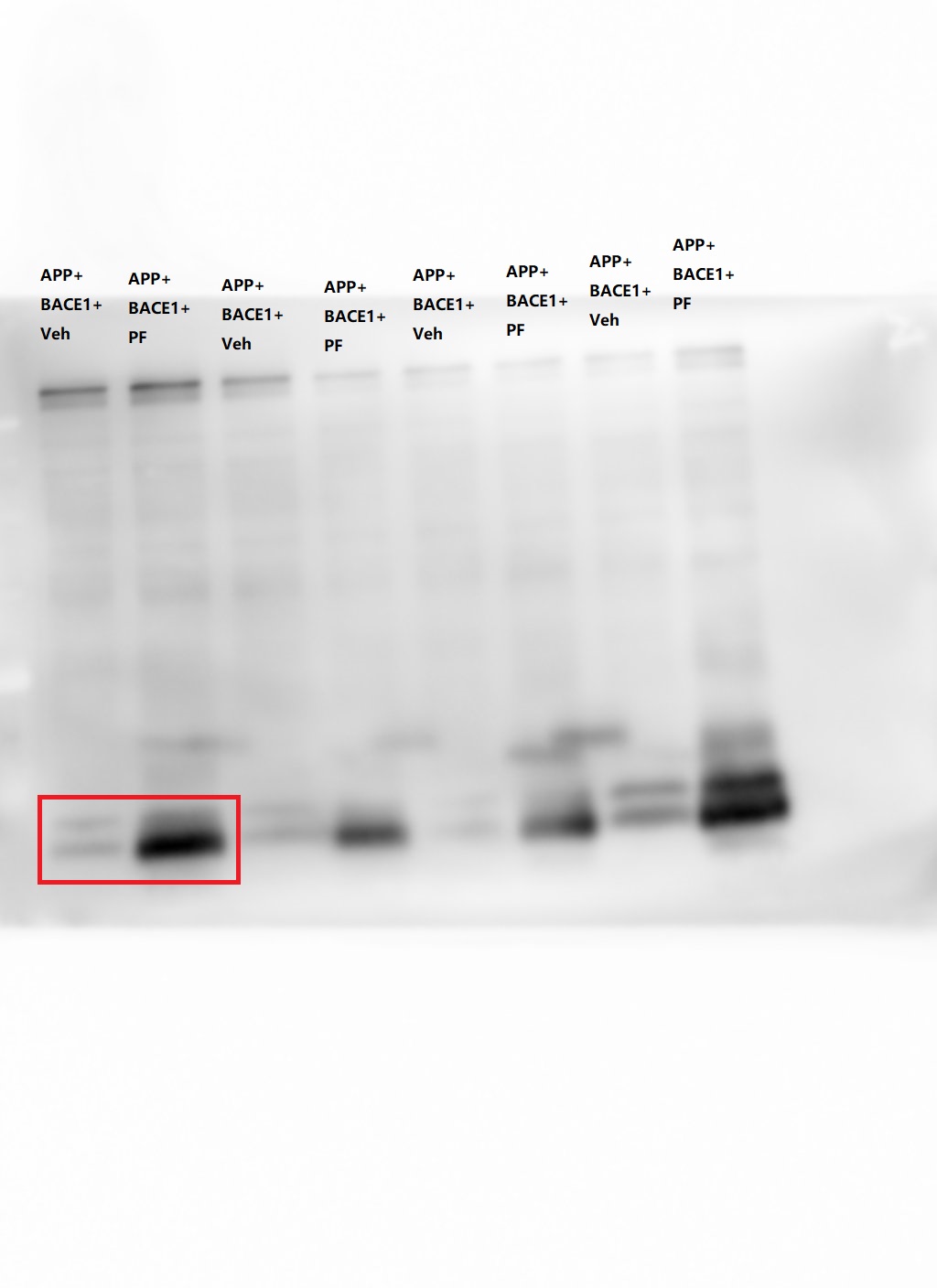

Supplement: Figure 2—source data 2. [file elife-100968-fig2-data2.zip › Figure 2J/CTFs-labelled.jpg]

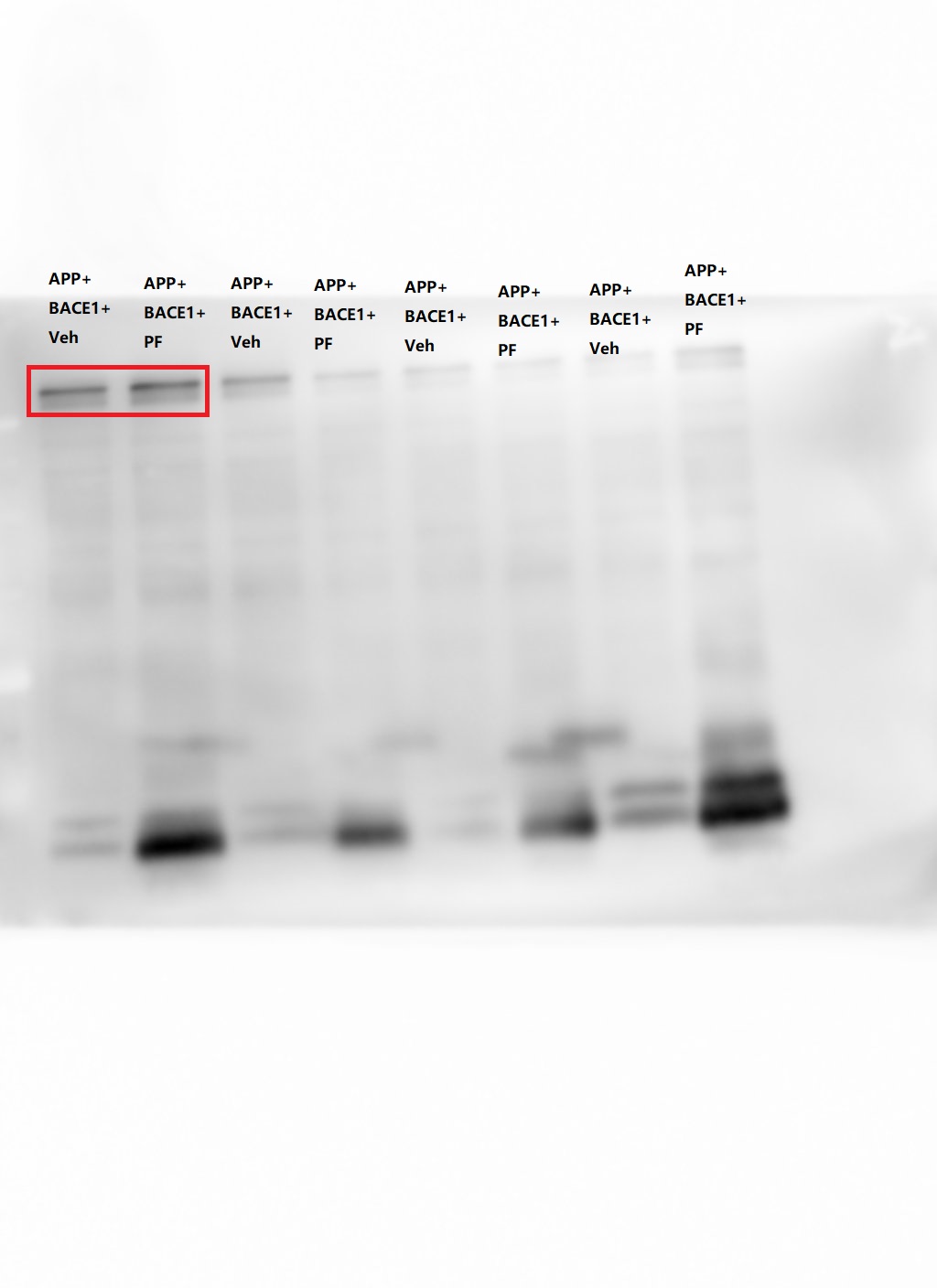

Supplement: Figure 2—source data 2. [file elife-100968-fig2-data2.zip › Figure 2J/FL-APP-labelled.jpg]

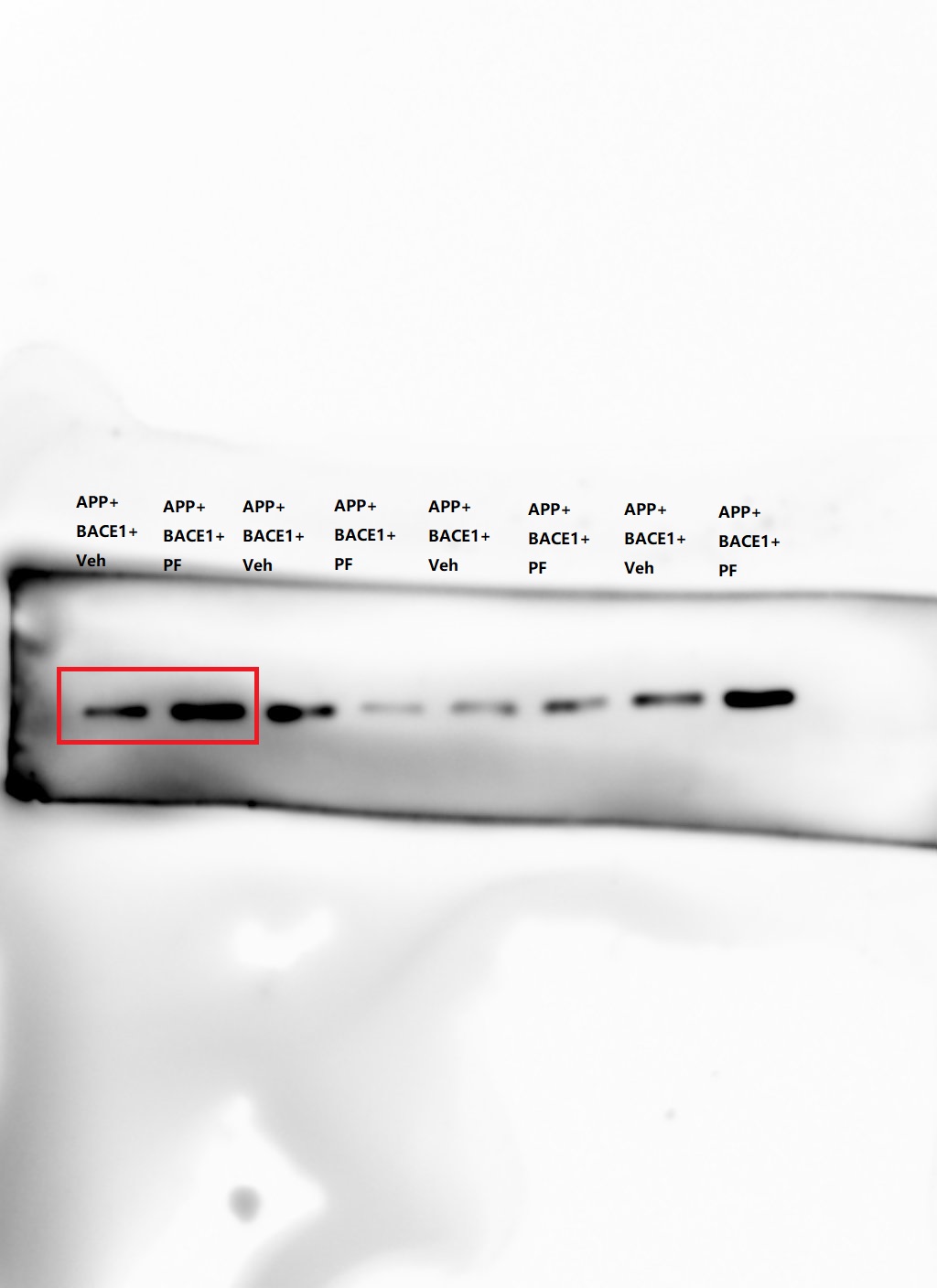

Supplement: Figure 2—source data 2. [file elife-100968-fig2-data2.zip › Figure 2J/GAPDH-labelled.jpg]

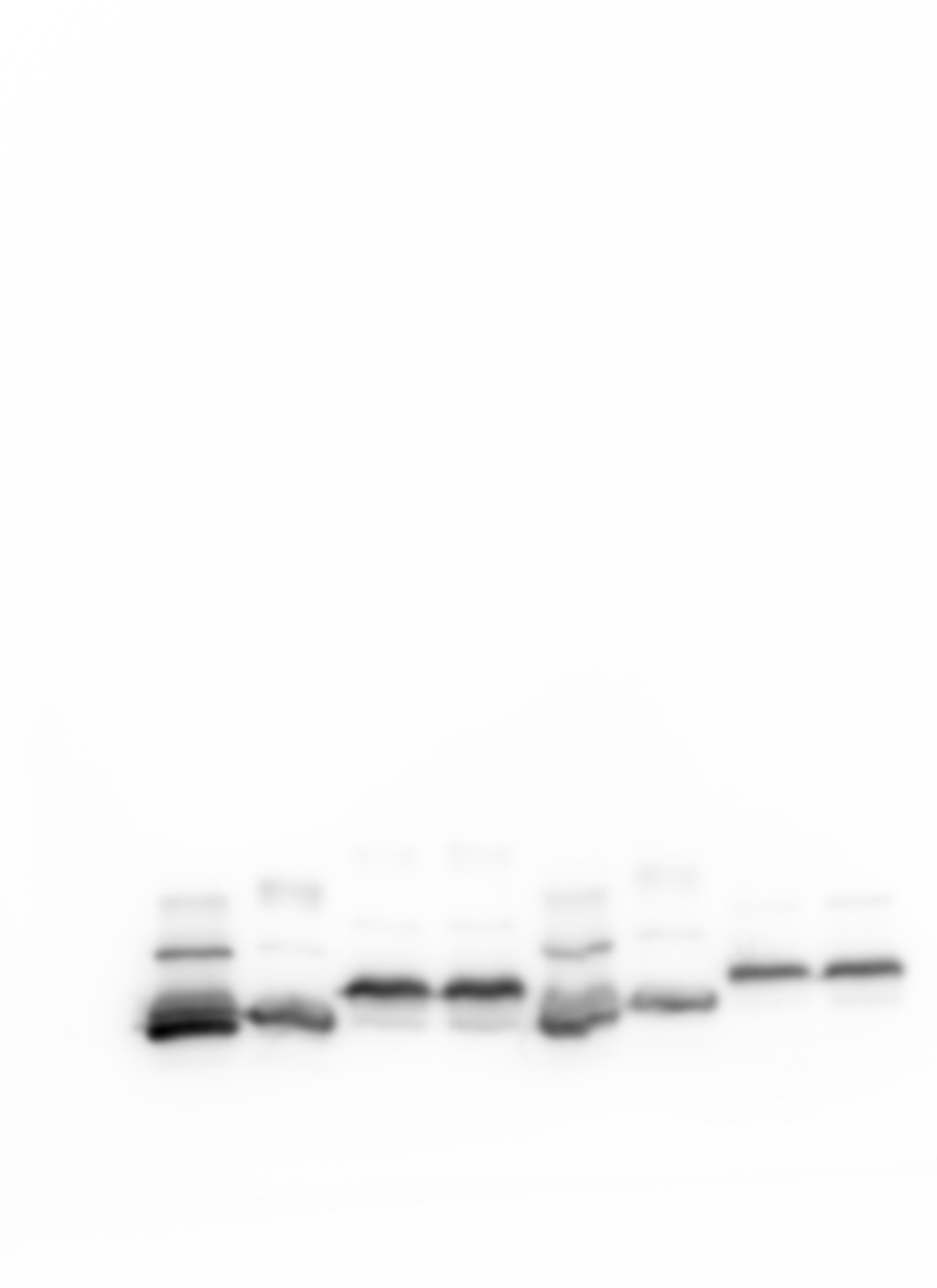

Supplement: Figure 5—source data 1. [file elife-100968-fig5-data1.zip › Figure 5F/flag-unedited gels.jpg]

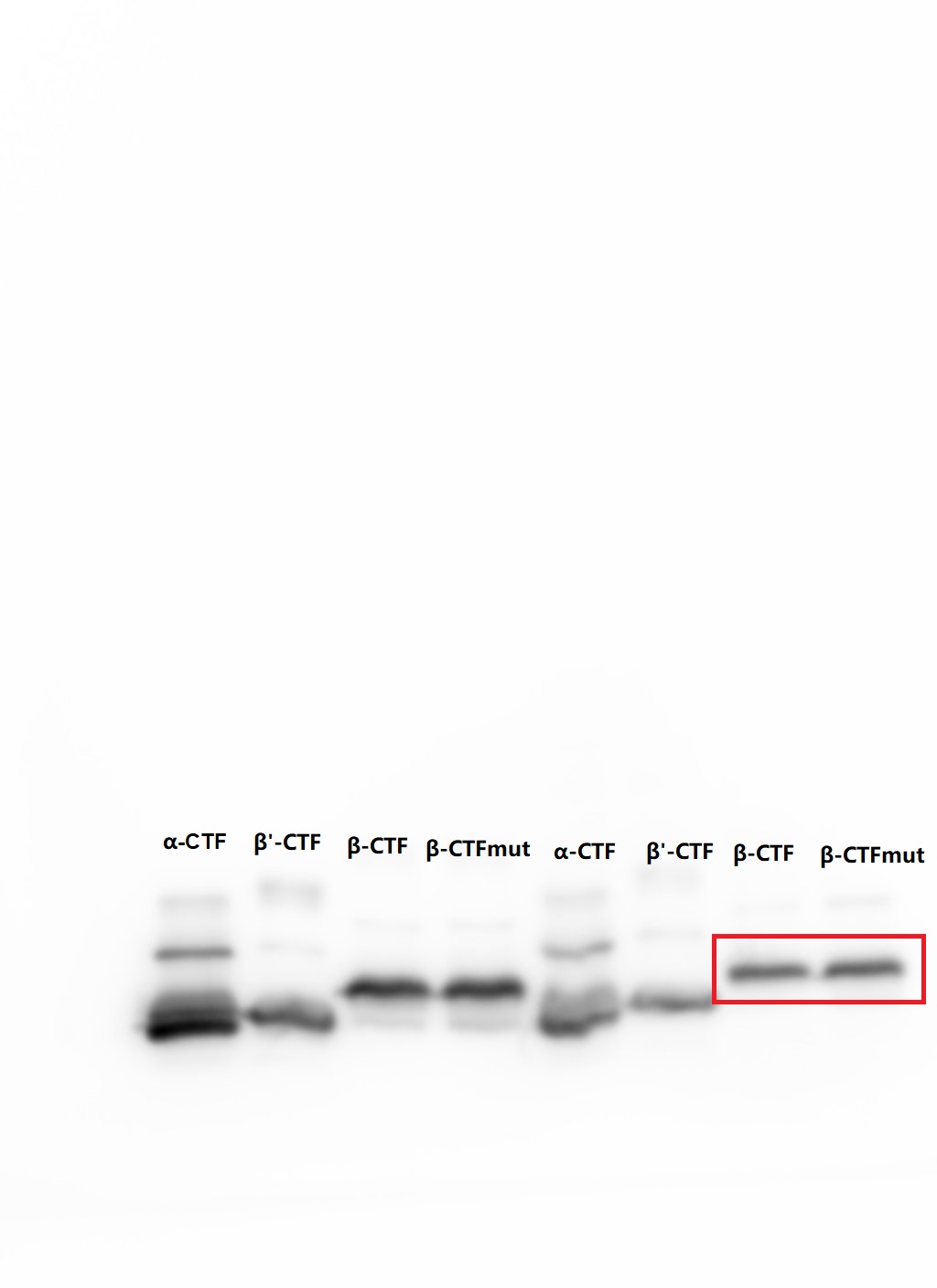

Supplement: Figure 5—source data 2. [file elife-100968-fig5-data2.zip › Figure 5F/flag-labelled.jpg]

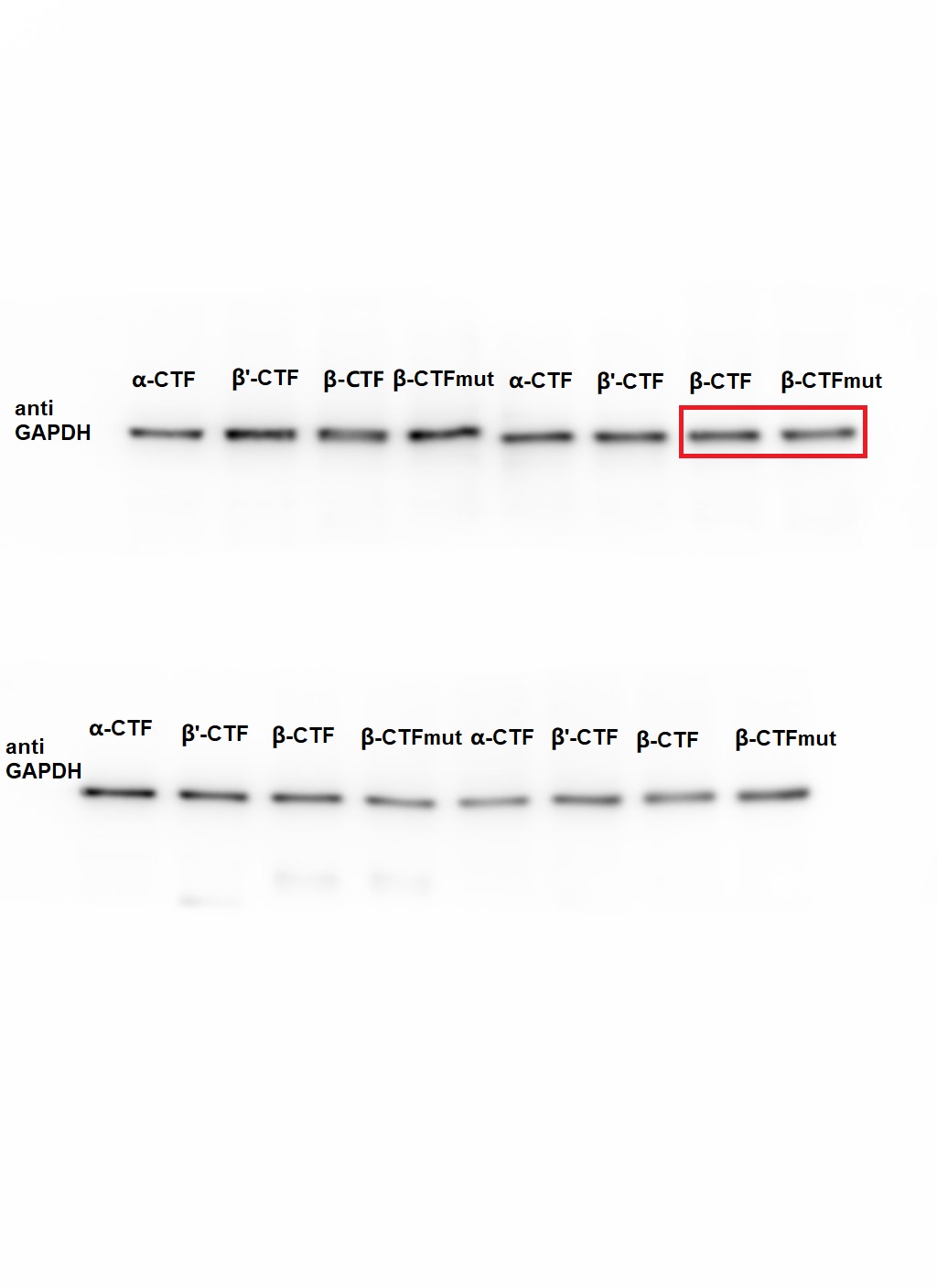

Supplement: Figure 5—source data 2. [file elife-100968-fig5-data2.zip › Figure 5F/GAPDH-labelled.jpg]

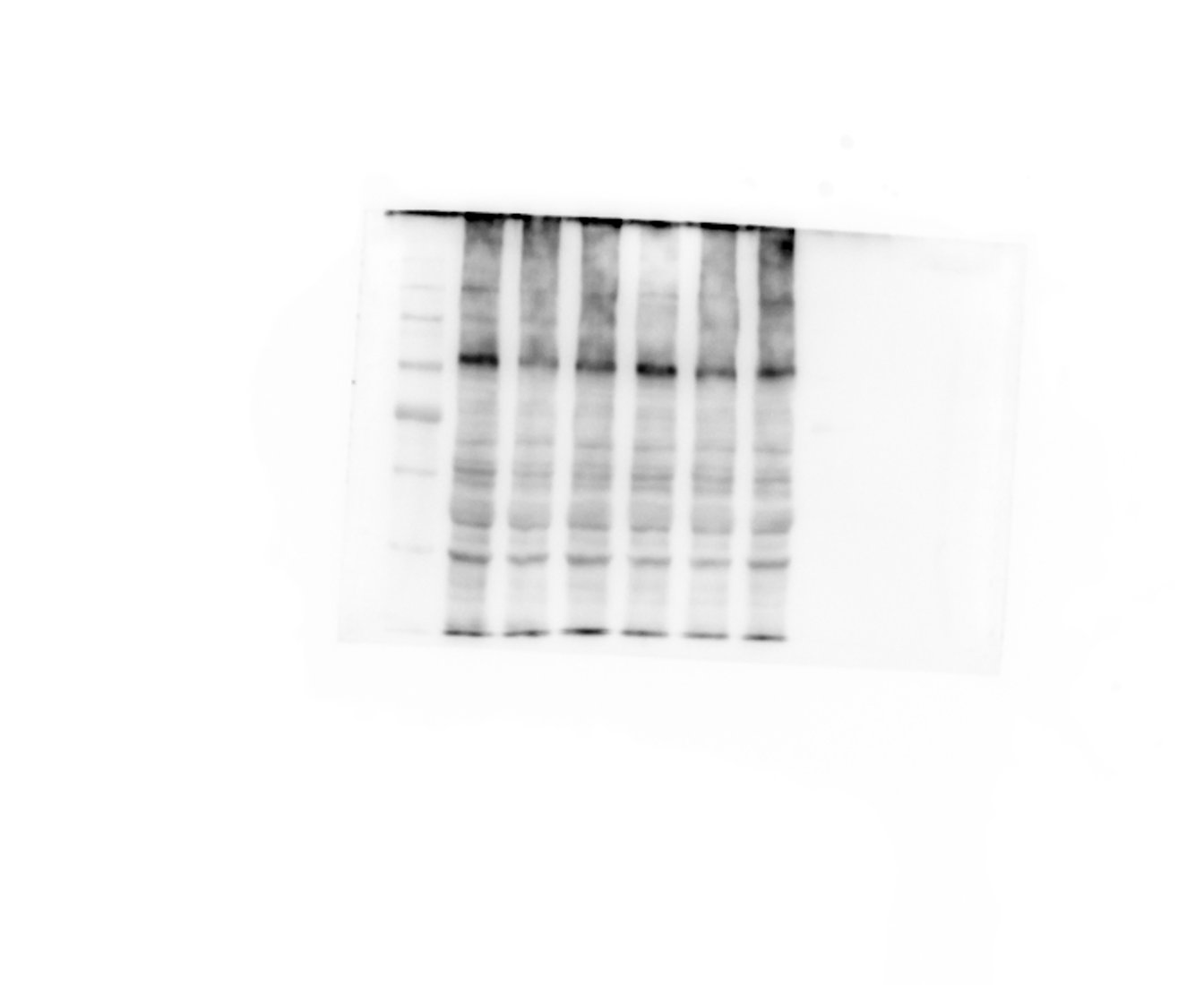

Supplement: Figure 6—source data 1. [file elife-100968-fig6-data1.zip › Figure 6C/GluR1-unedited gels.jpg]

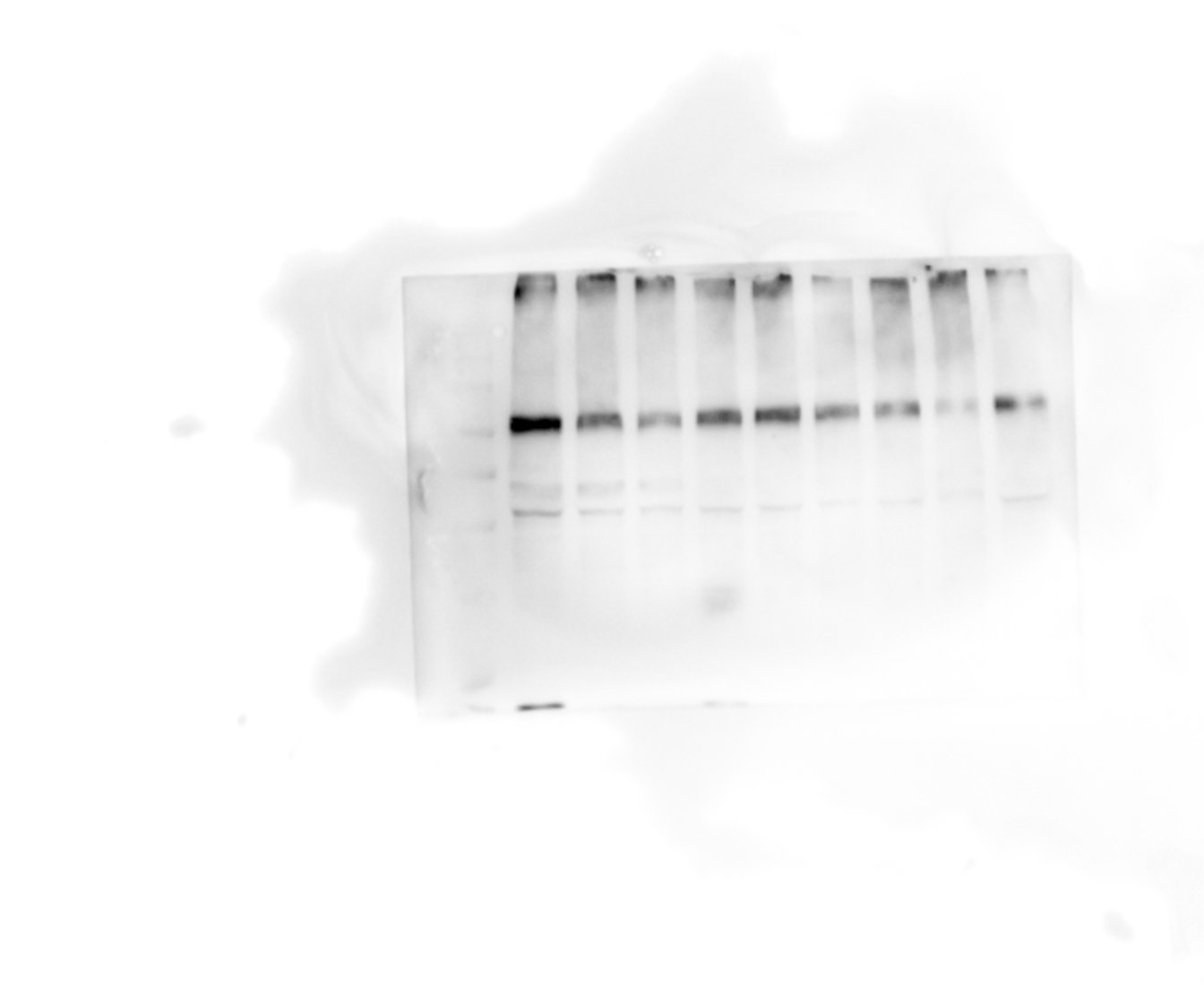

Supplement: Figure 6—source data 1. [file elife-100968-fig6-data1.zip › Figure 6C/GluR2-unedited gels.jpg]

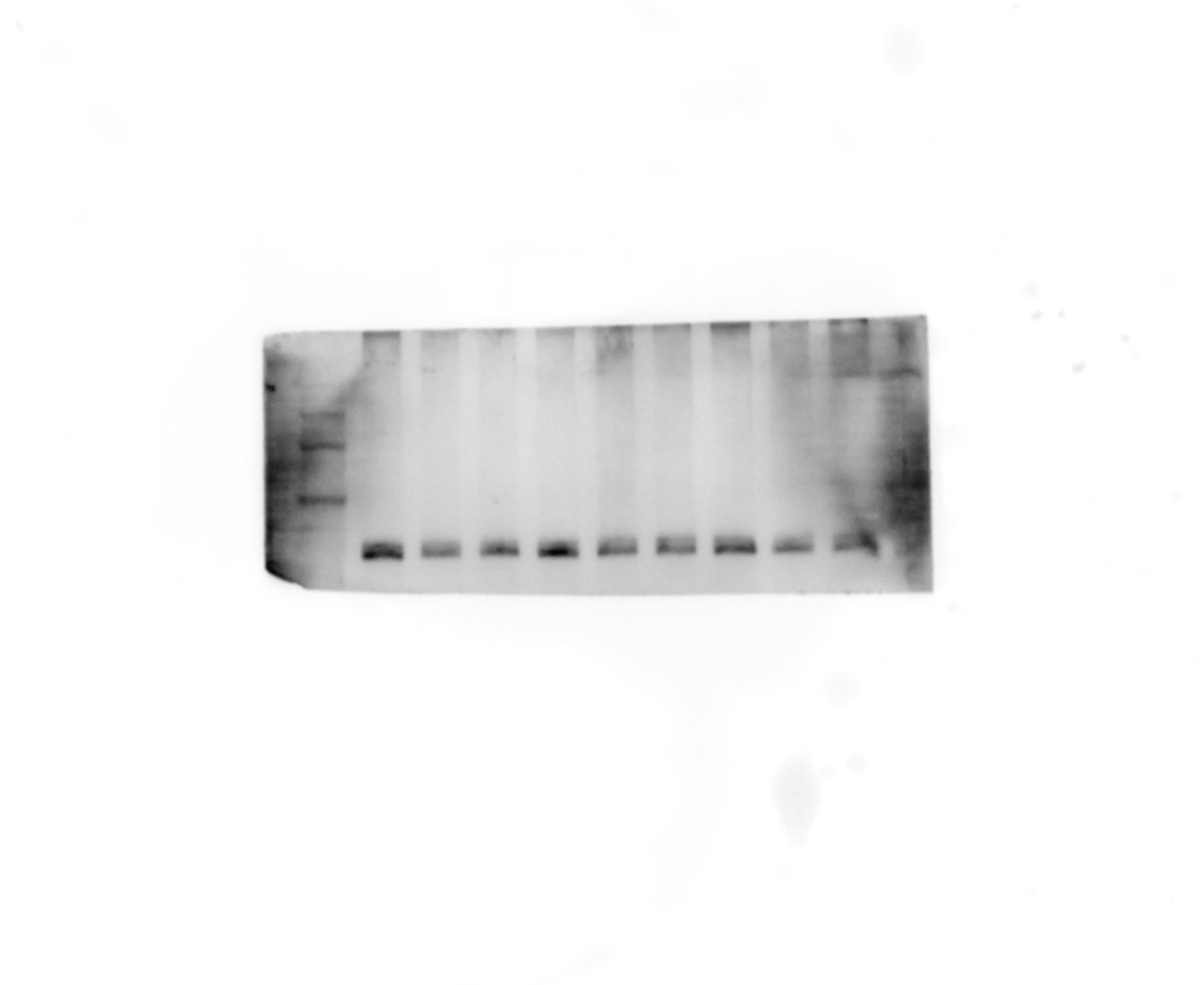

Supplement: Figure 6—source data 1. [file elife-100968-fig6-data1.zip › Figure 6C/Synapsin 1-unedited gels.jpg]

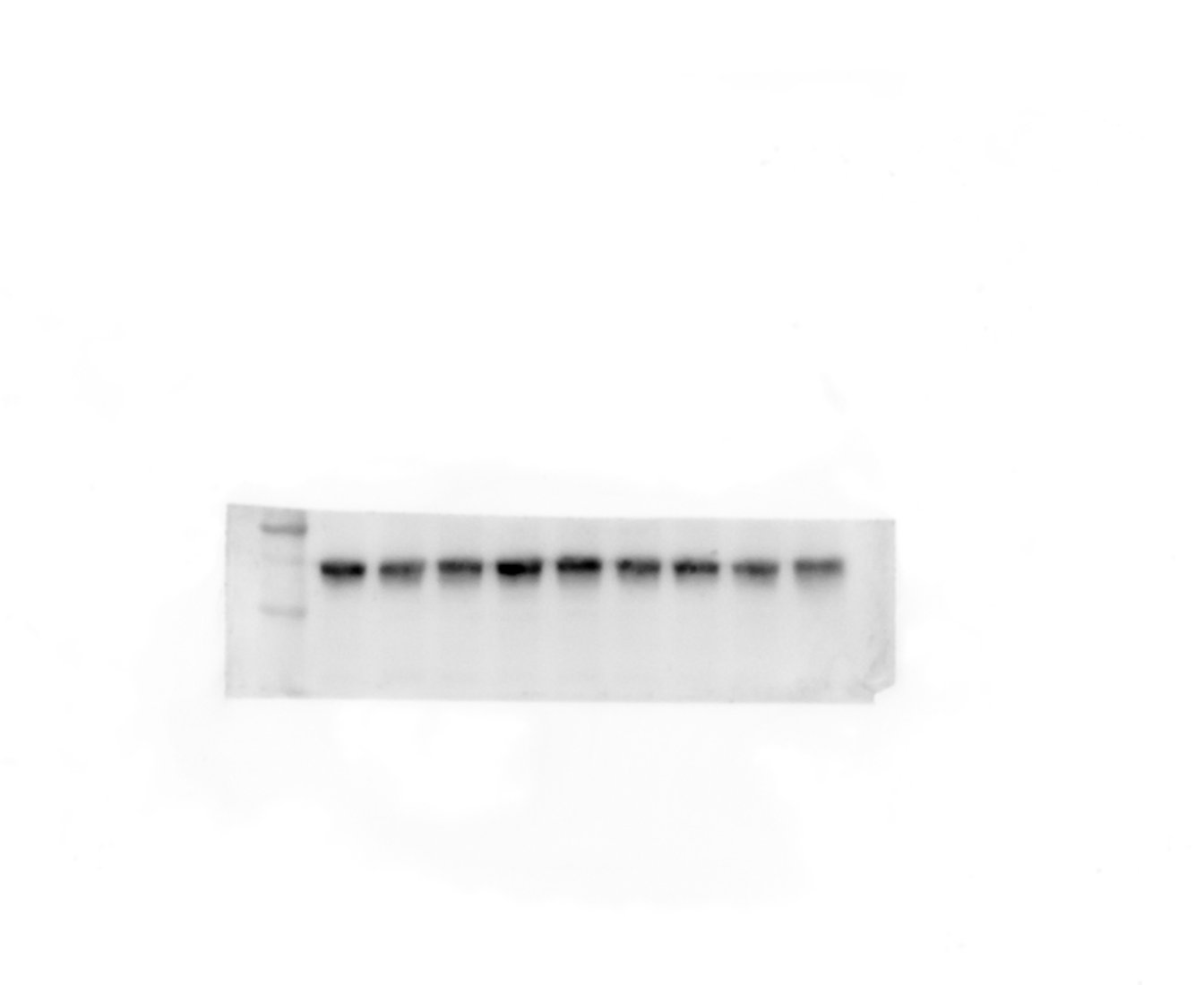

Supplement: Figure 6—source data 1. [file elife-100968-fig6-data1.zip › Figure 6C/Tuj1-unedited gels.jpg]

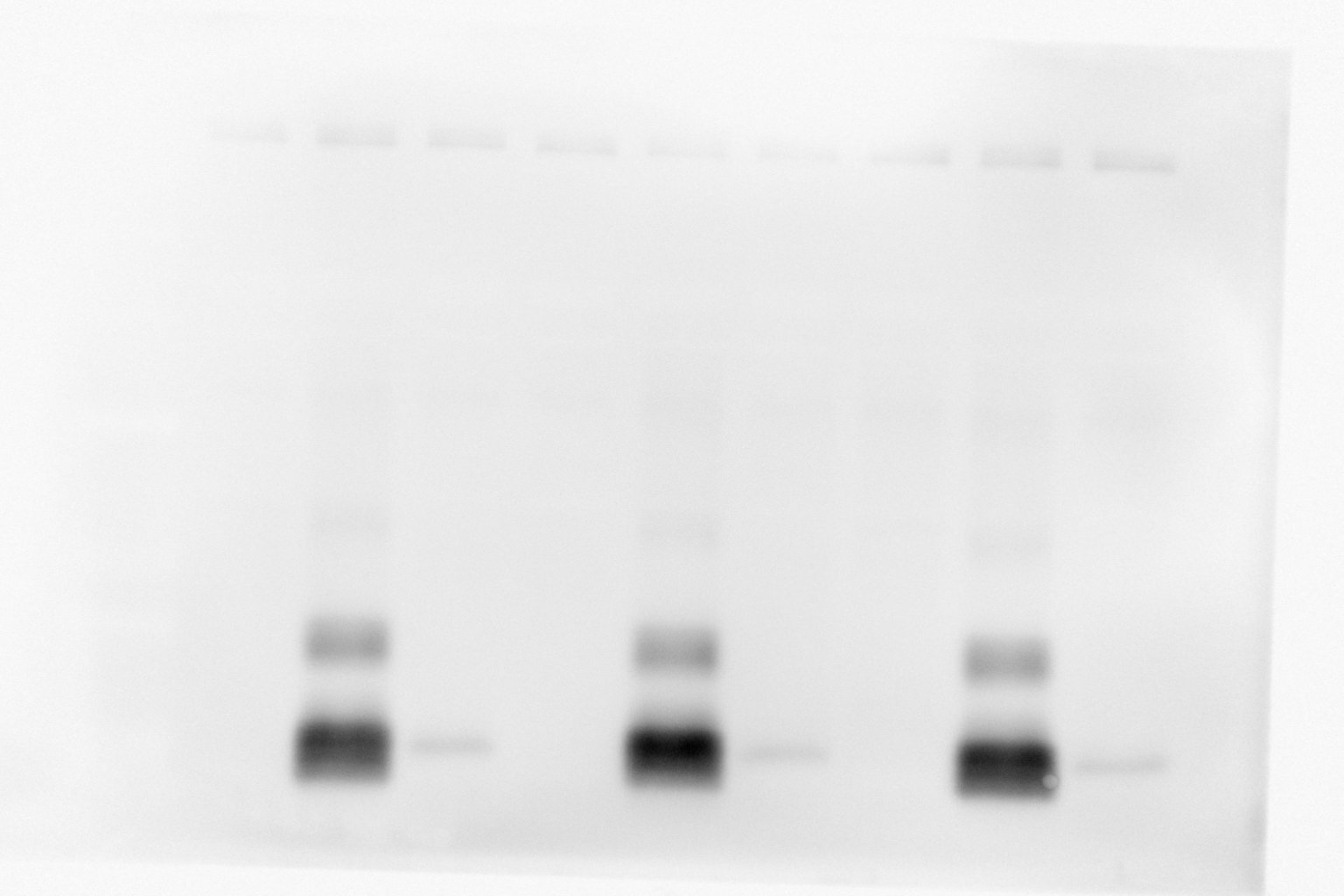

Supplement: Figure 6—source data 1. [file elife-100968-fig6-data1.zip › Figure 6C/a┬-CTF-unedited gels.jpg]

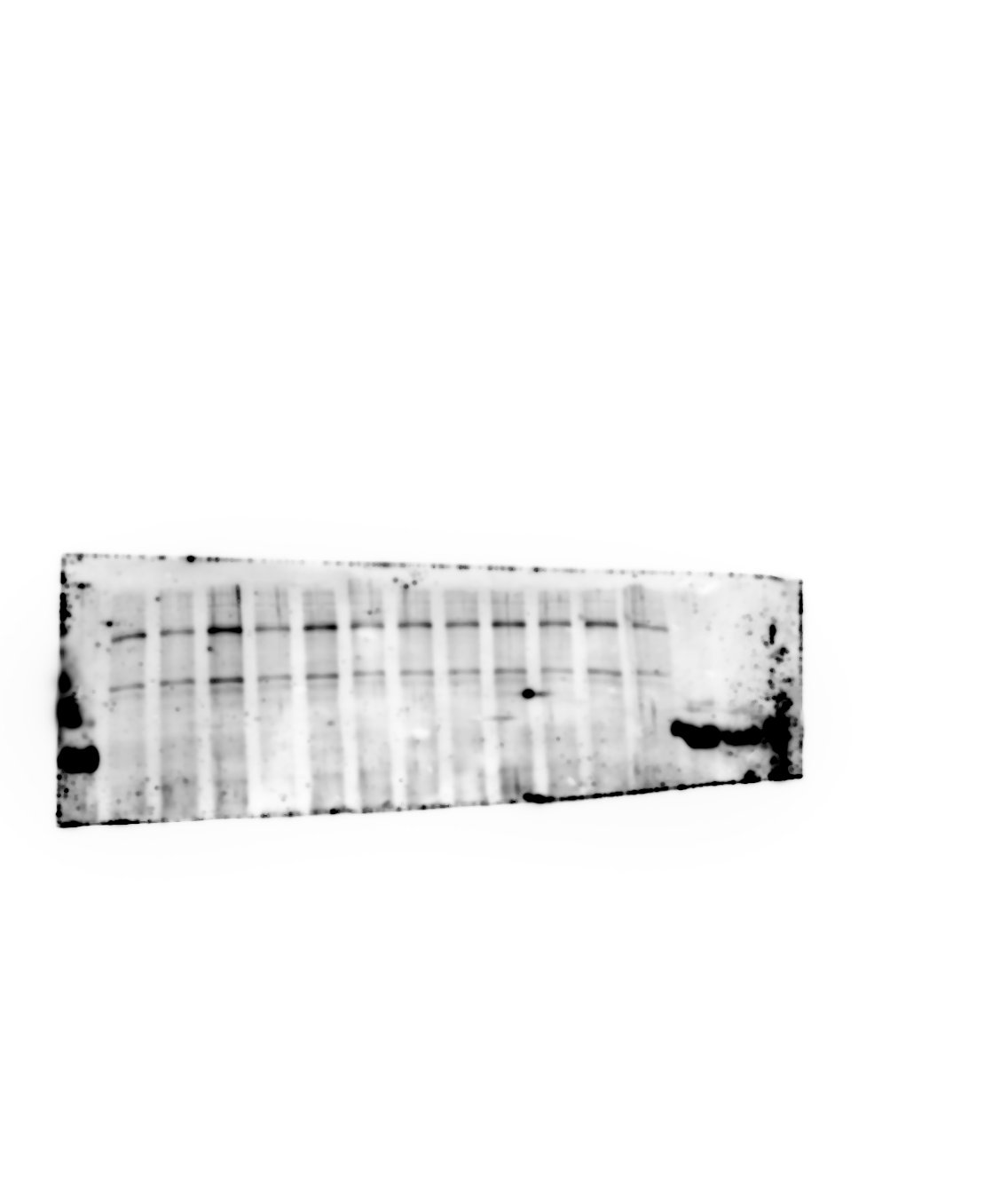

Supplement: Figure 6—source data 1. [file elife-100968-fig6-data1.zip › Figure 6E/GluN2A-unedited gels.jpg]

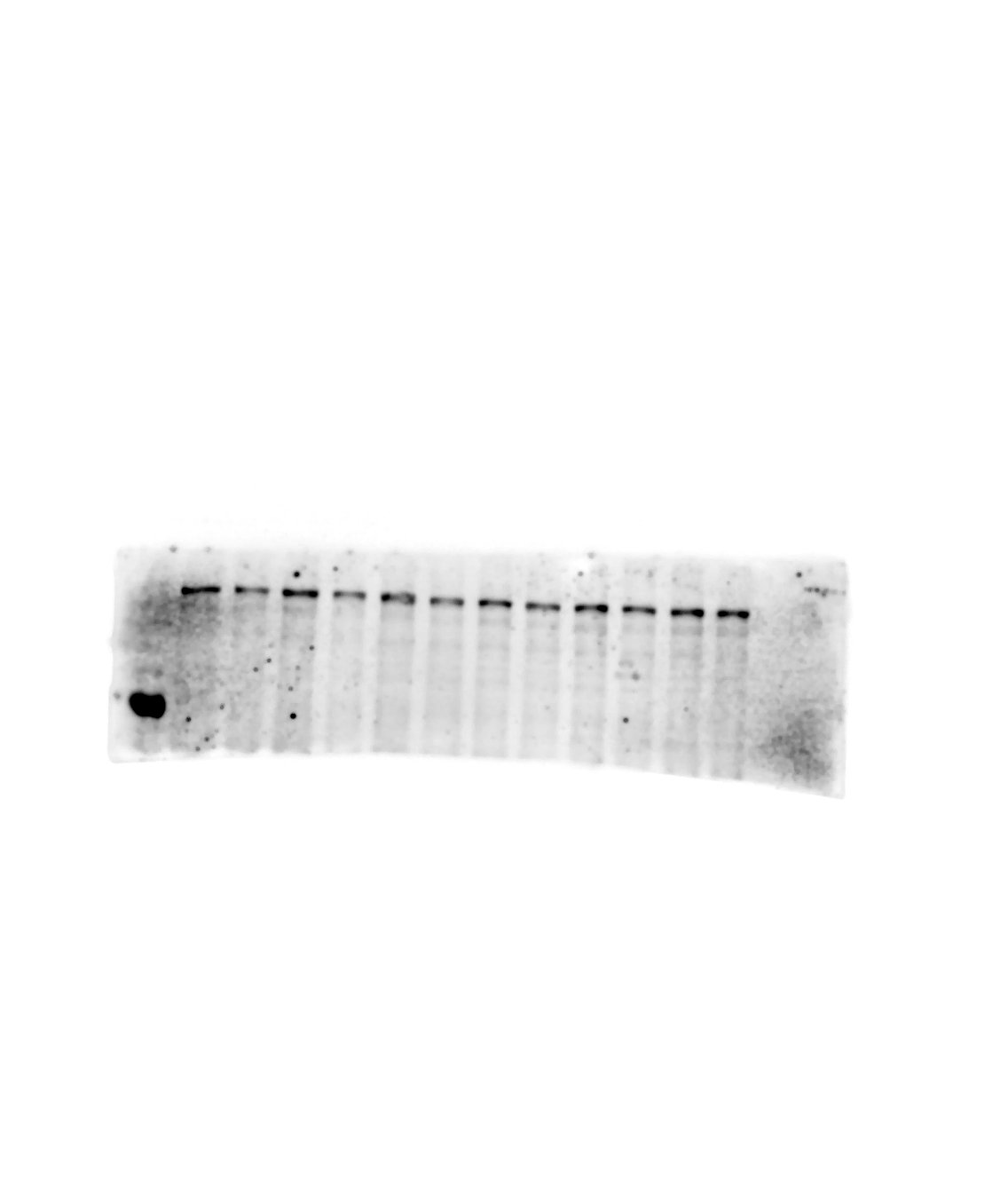

Supplement: Figure 6—source data 1. [file elife-100968-fig6-data1.zip › Figure 6E/GluN2B-unedited gels.jpg]

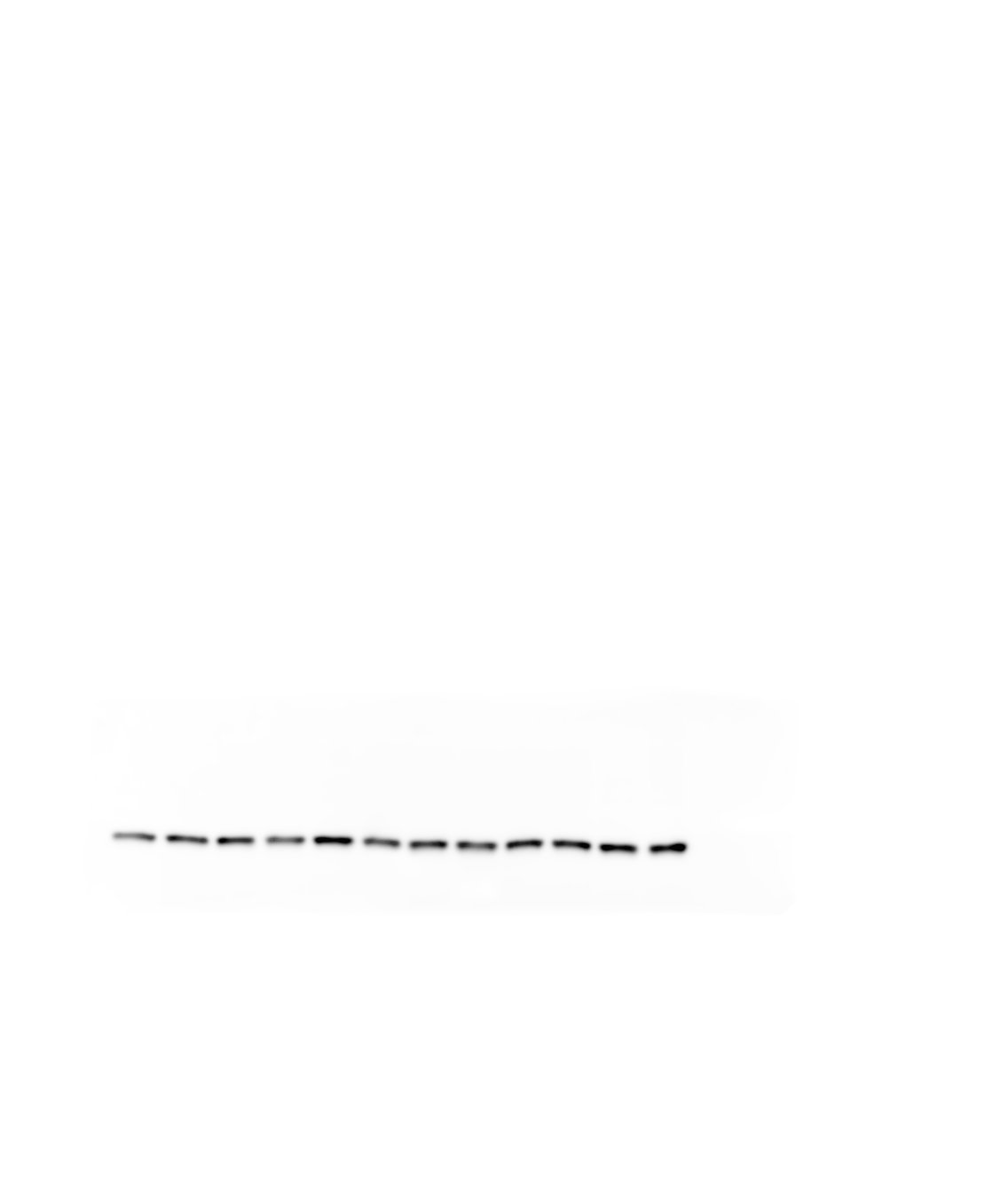

Supplement: Figure 6—source data 1. [file elife-100968-fig6-data1.zip › Figure 6E/syntaxin1A-unedited gels.jpg]

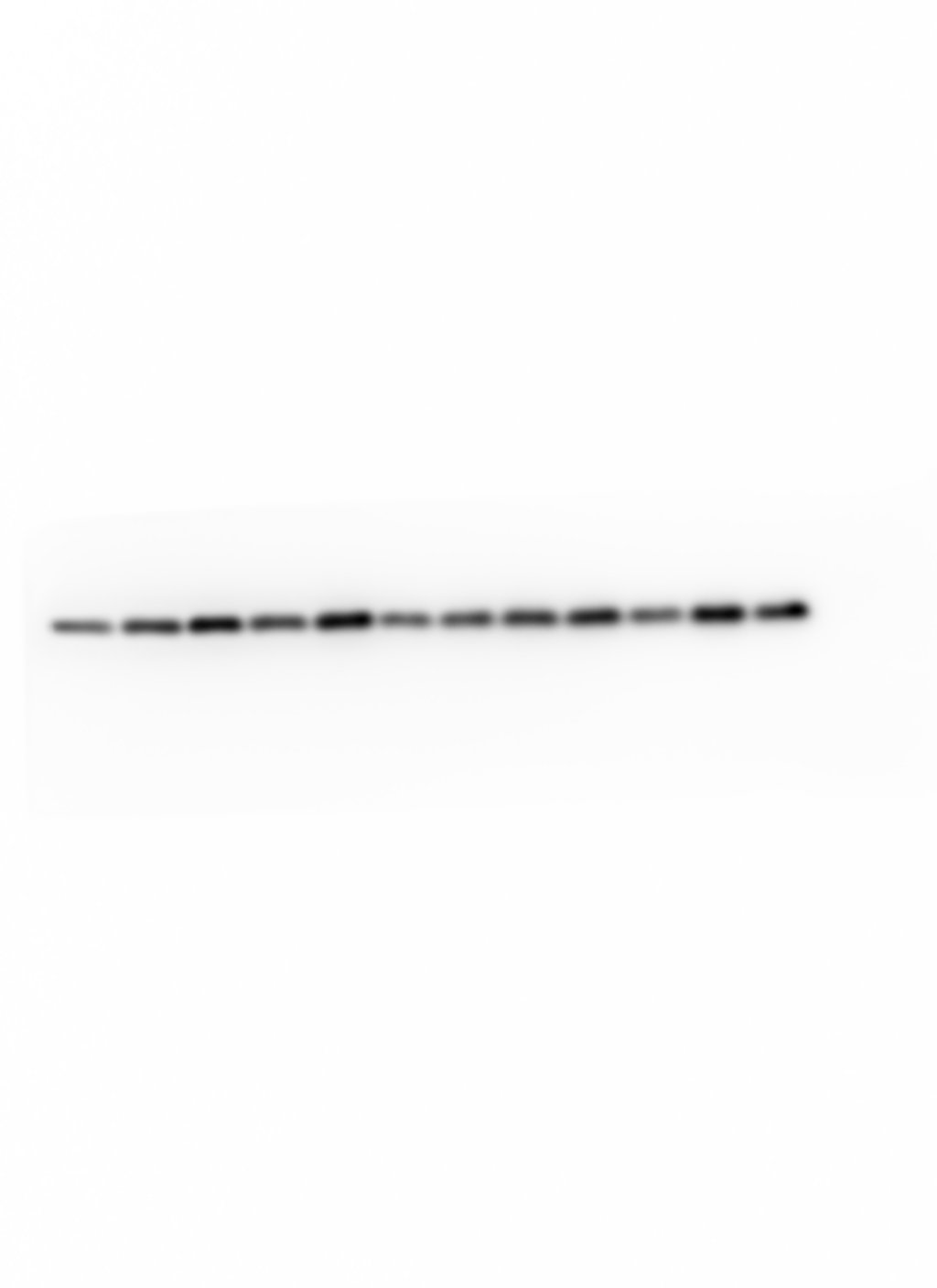

Supplement: Figure 6—source data 1. [file elife-100968-fig6-data1.zip › Figure 6E/vamp2-unedited gels.jpg]

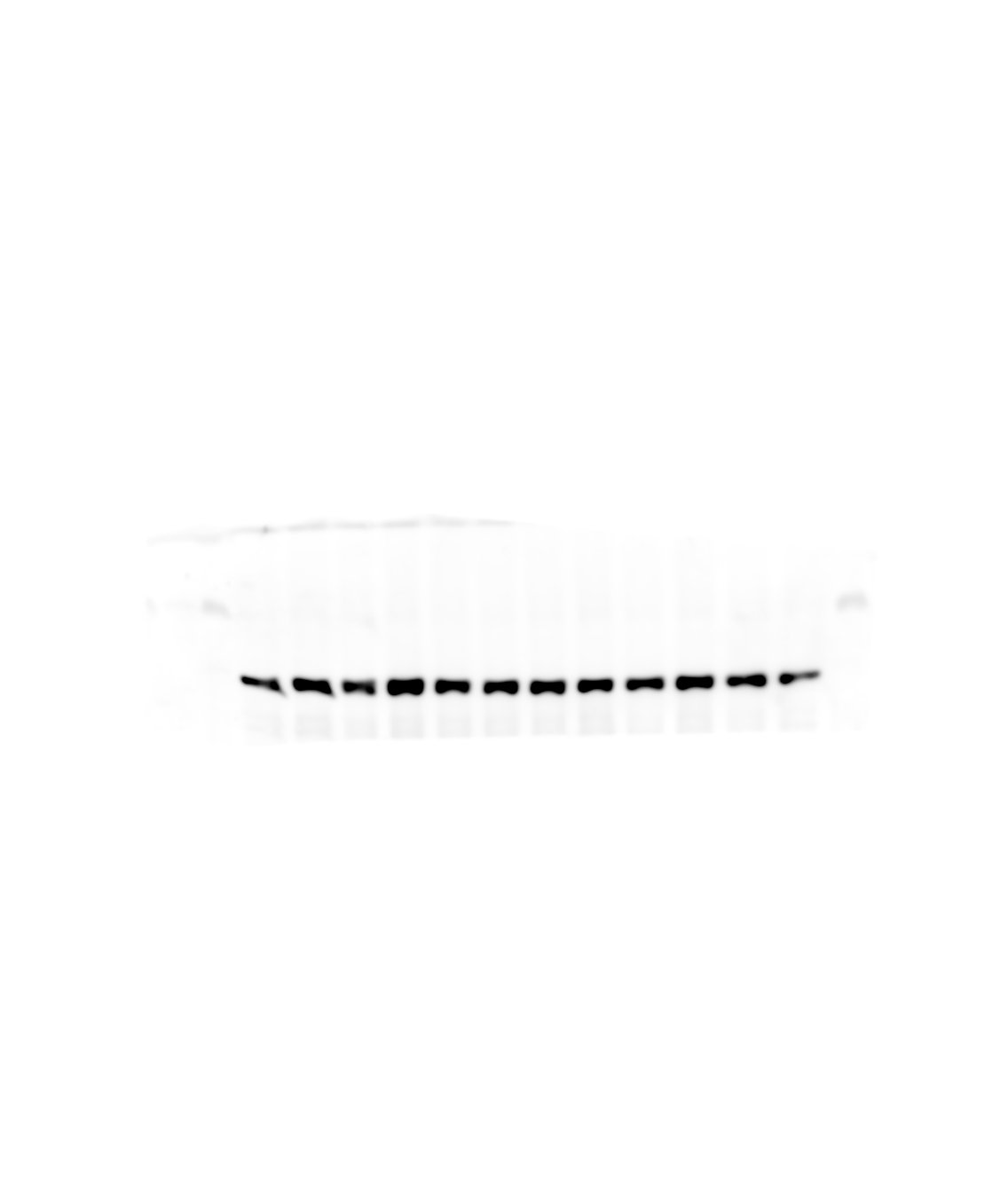

Supplement: Figure 6—source data 1. [file elife-100968-fig6-data1.zip › Figure 6E/a┬-actin-unedited gels.jpg]

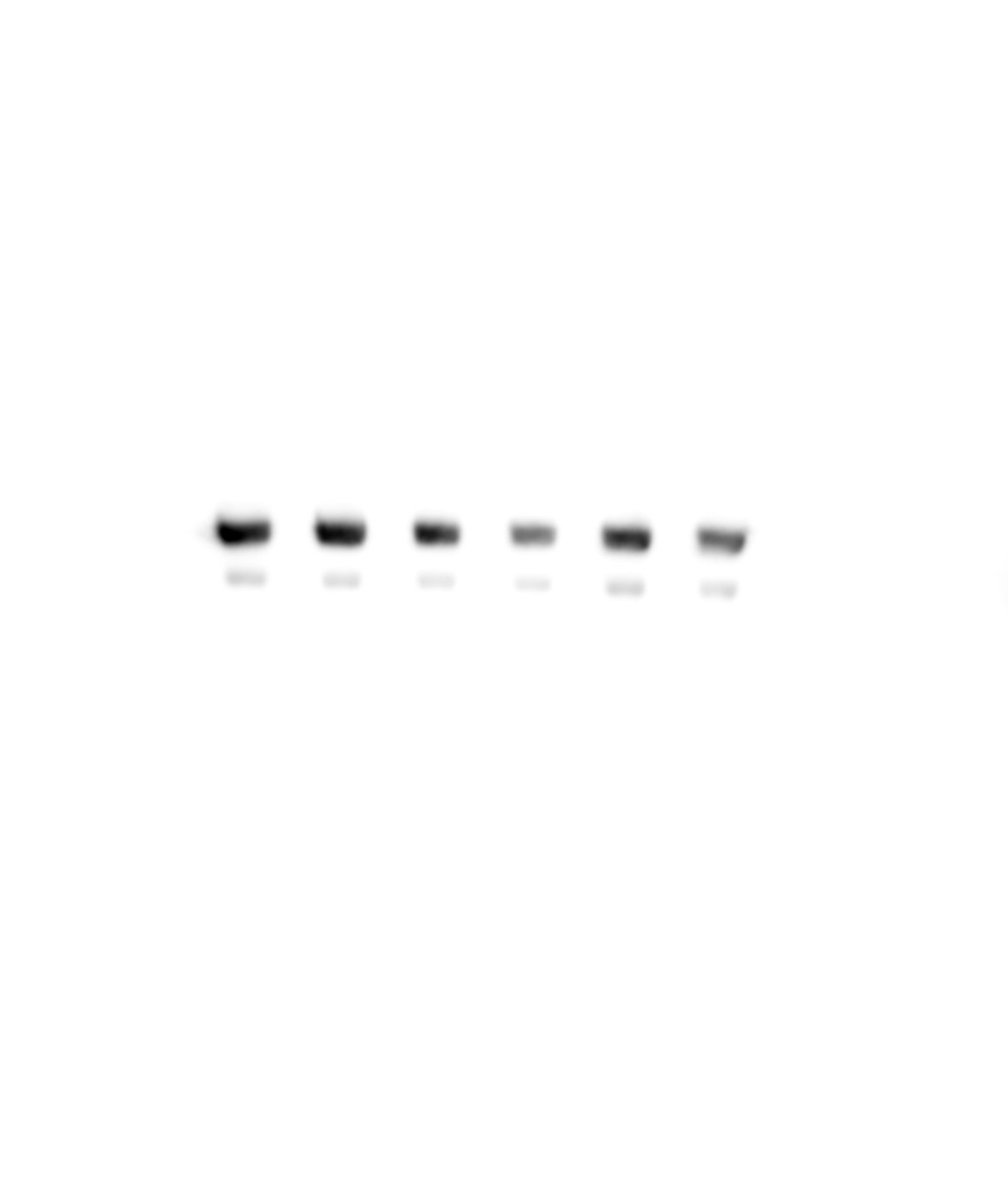

Supplement: Figure 6—source data 1. [file elife-100968-fig6-data1.zip › Figure 6E/a┬-CTF-unedited gels.jpg]

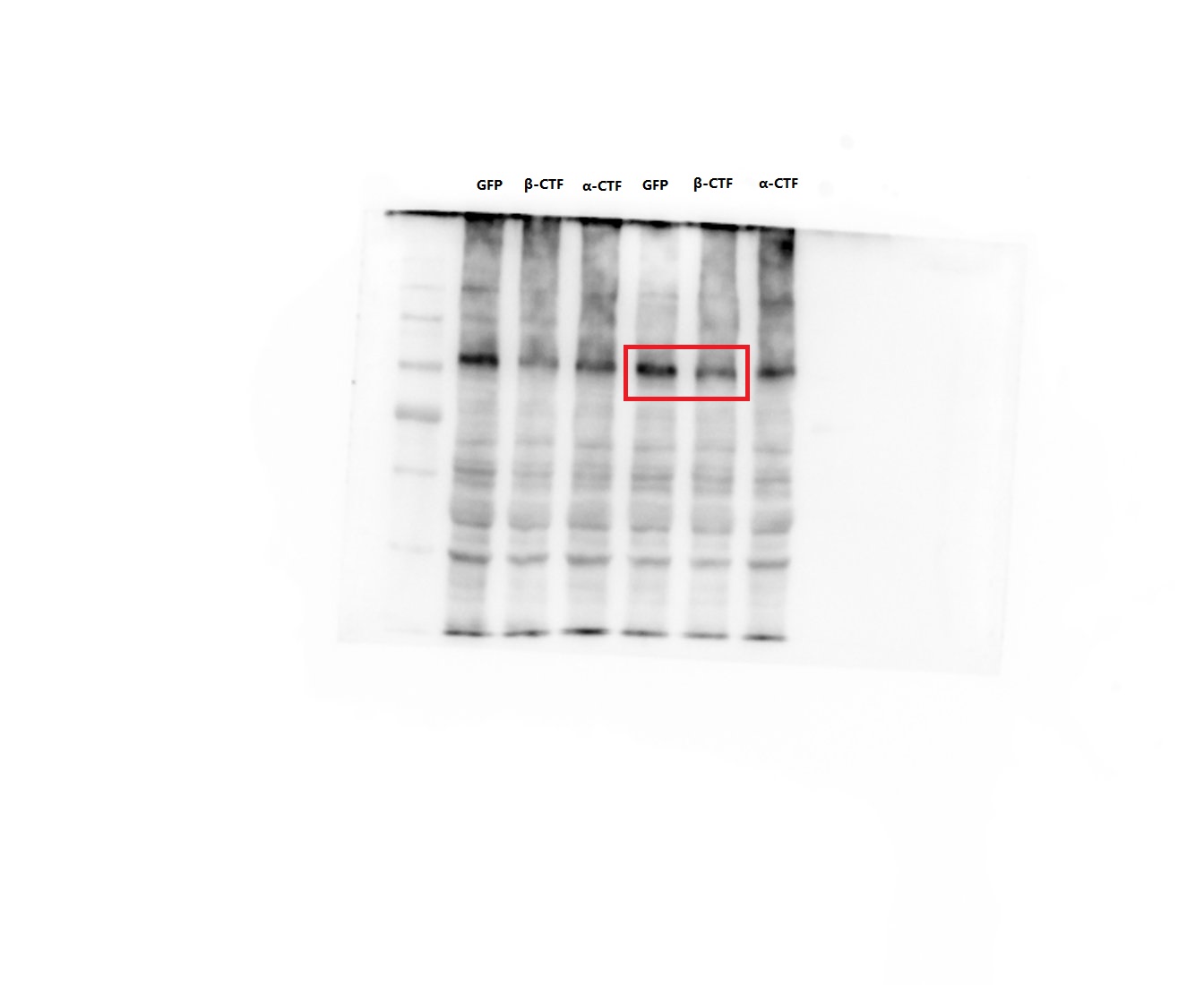

Supplement: Figure 6—source data 2. [file elife-100968-fig6-data2.zip › Figure 6C/GluR1-labelled.jpg]

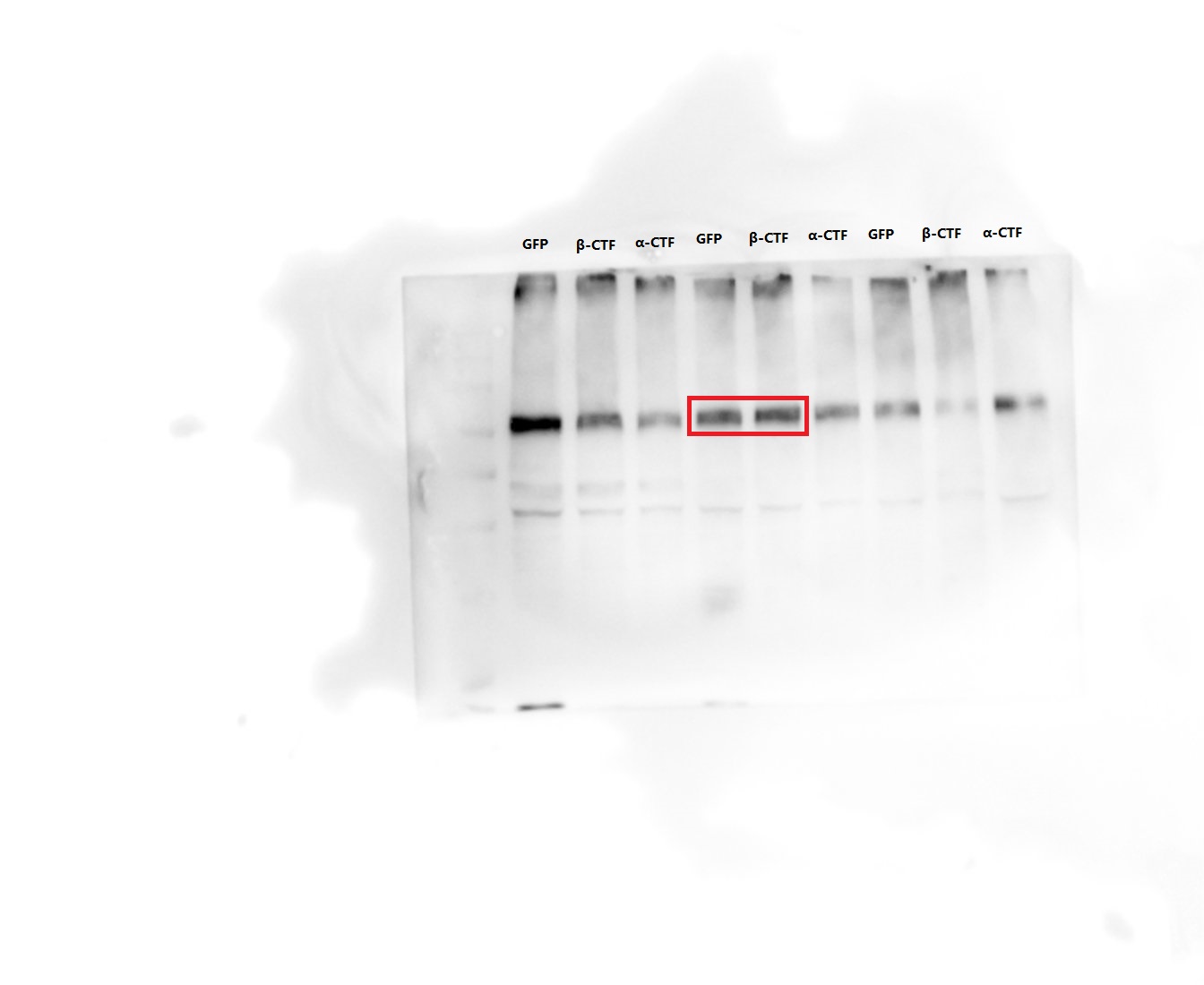

Supplement: Figure 6—source data 2. [file elife-100968-fig6-data2.zip › Figure 6C/GluR2-labelled.jpg]

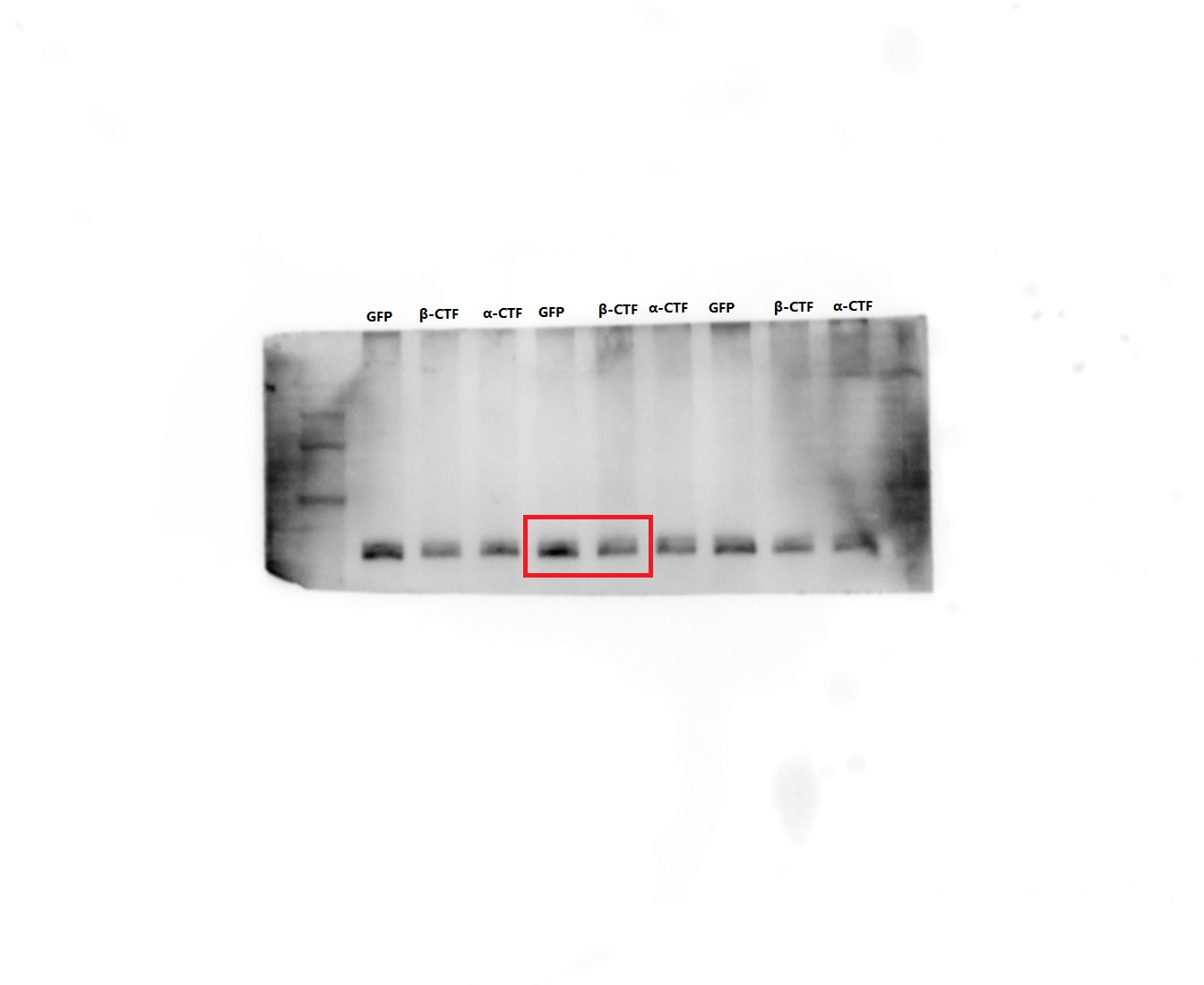

Supplement: Figure 6—source data 2. [file elife-100968-fig6-data2.zip › Figure 6C/Synapsin 1-labelled.jpg]

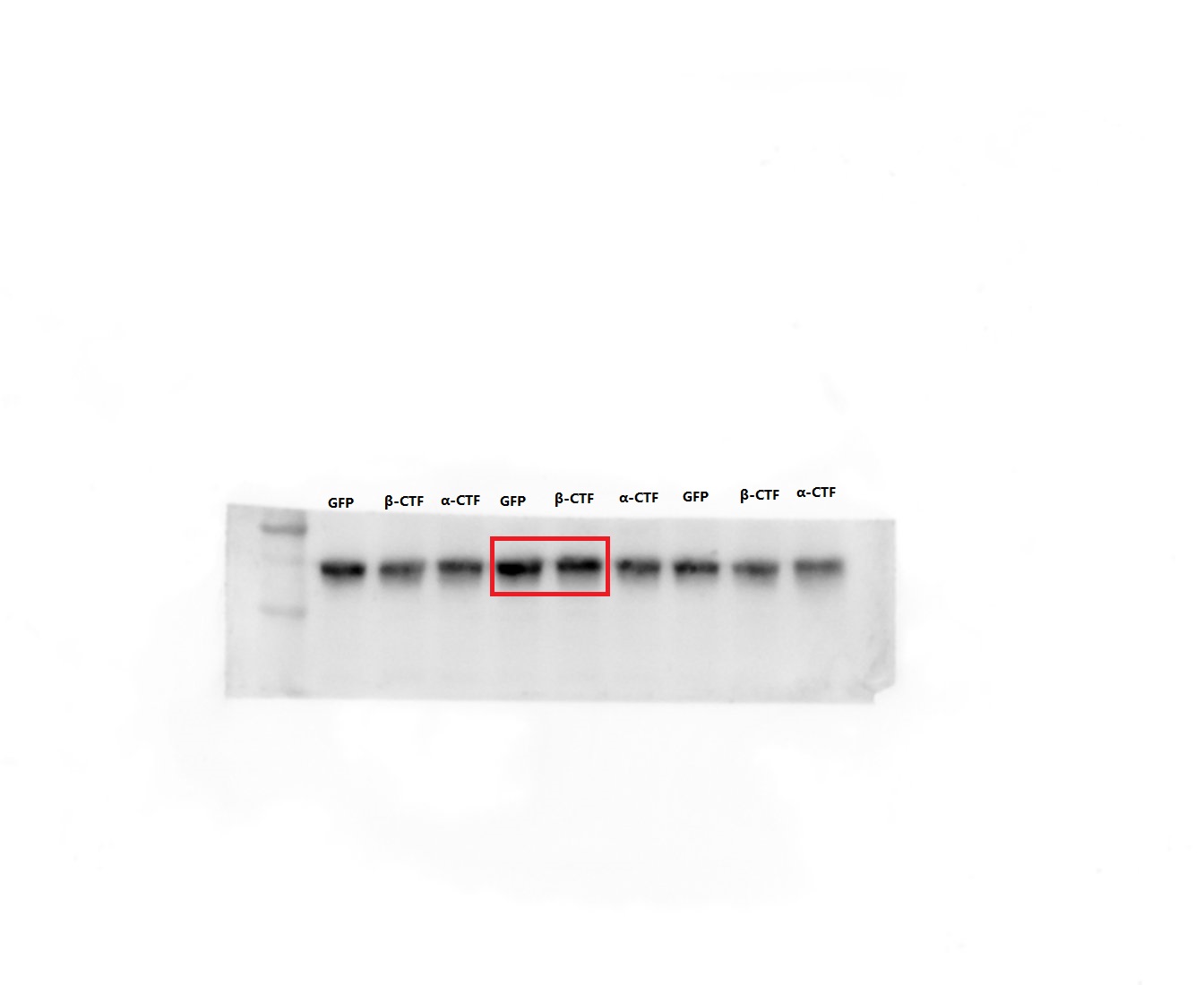

Supplement: Figure 6—source data 2. [file elife-100968-fig6-data2.zip › Figure 6C/Tuj1-labelled.jpg]

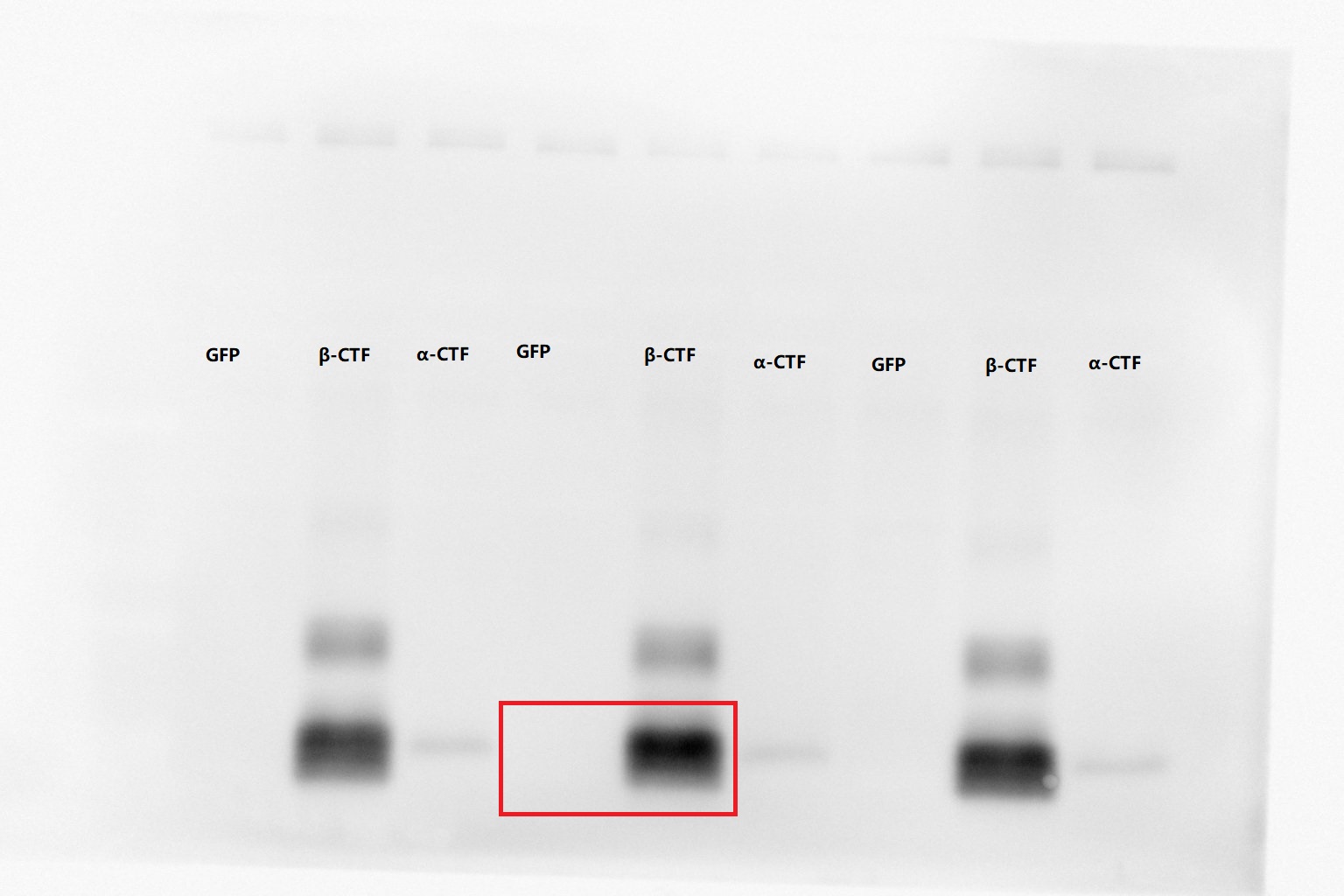

Supplement: Figure 6—source data 2. [file elife-100968-fig6-data2.zip › Figure 6C/a┬-CTF-labelled.jpg]

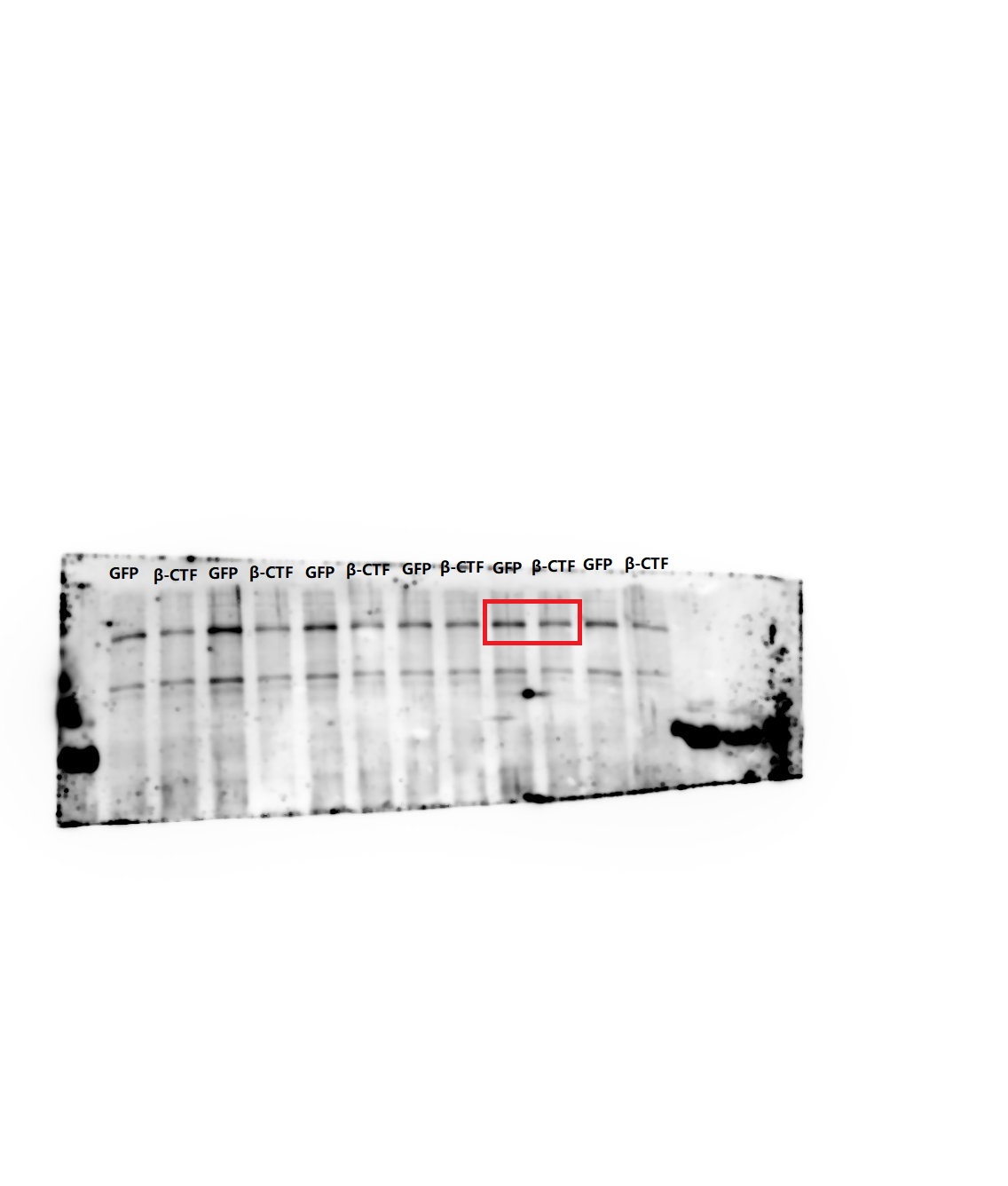

Supplement: Figure 6—source data 2. [file elife-100968-fig6-data2.zip › Figure 6E/GluN2A-labelled.jpg]

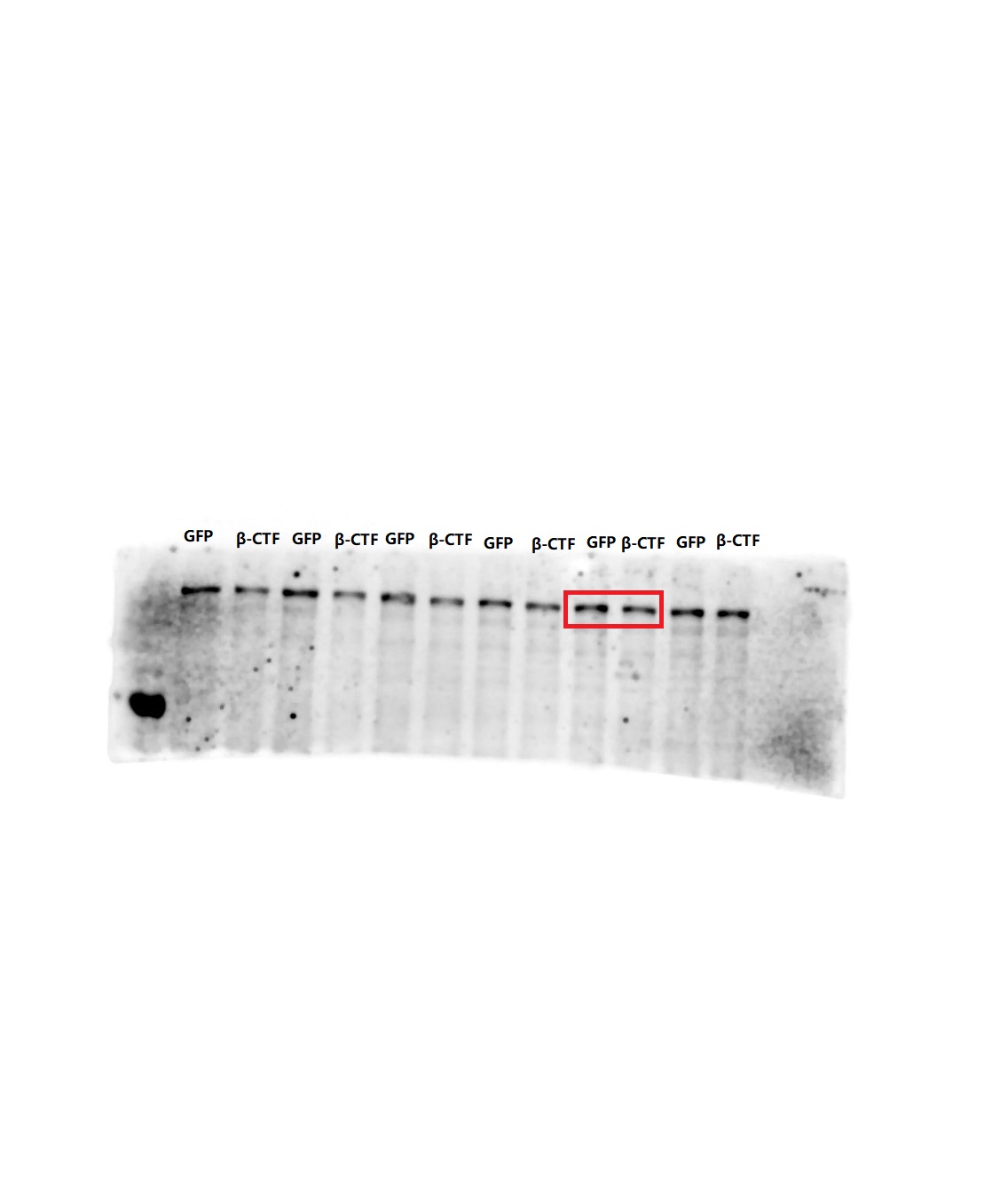

Supplement: Figure 6—source data 2. [file elife-100968-fig6-data2.zip › Figure 6E/GluN2B-labelled.jpg]

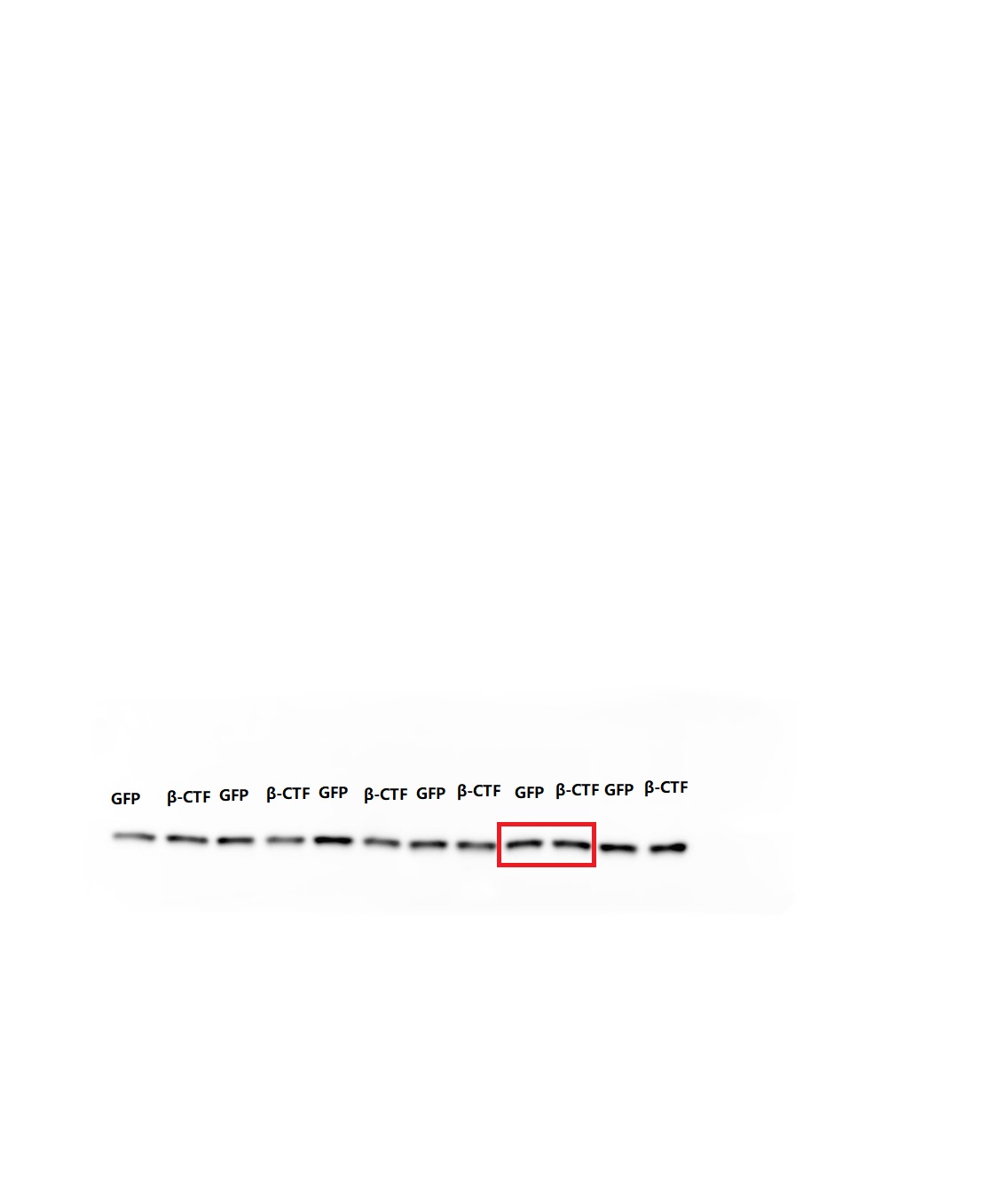

Supplement: Figure 6—source data 2. [file elife-100968-fig6-data2.zip › Figure 6E/syntaxin1A-labelled.jpg]

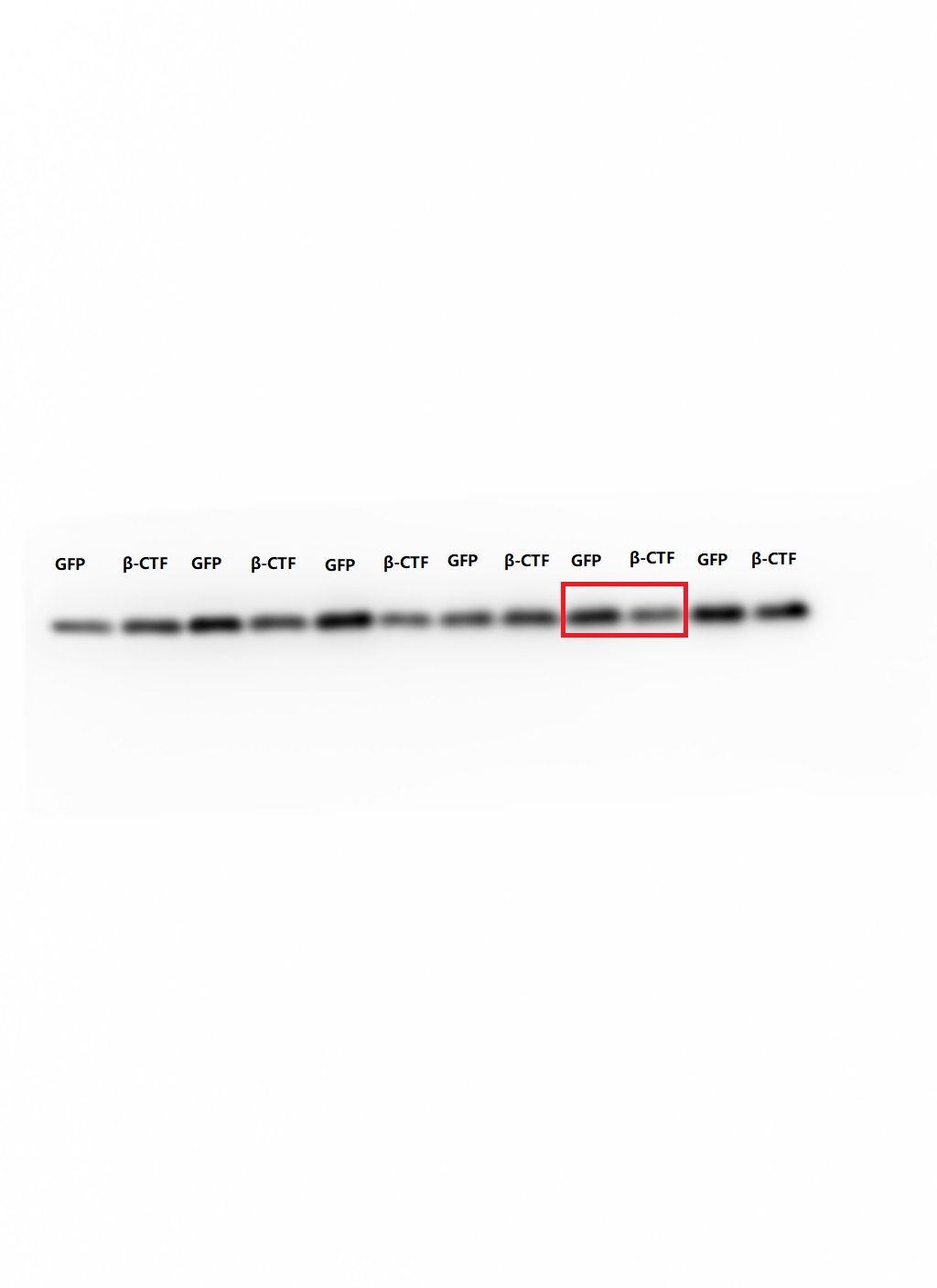

Supplement: Figure 6—source data 2. [file elife-100968-fig6-data2.zip › Figure 6E/vamp2-labelled.jpg]

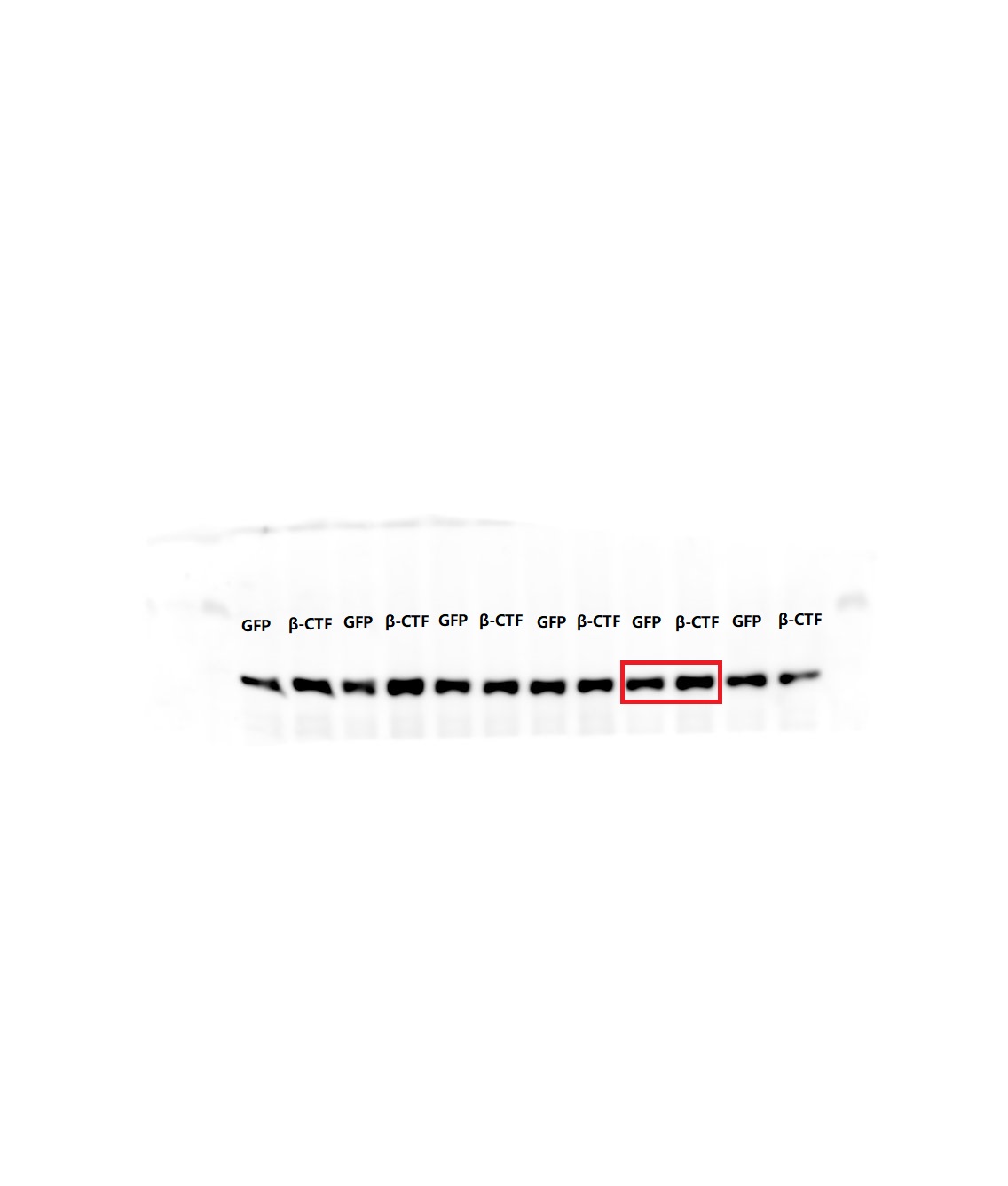

Supplement: Figure 6—source data 2. [file elife-100968-fig6-data2.zip › Figure 6E/a┬-actin-labellled.jpg]

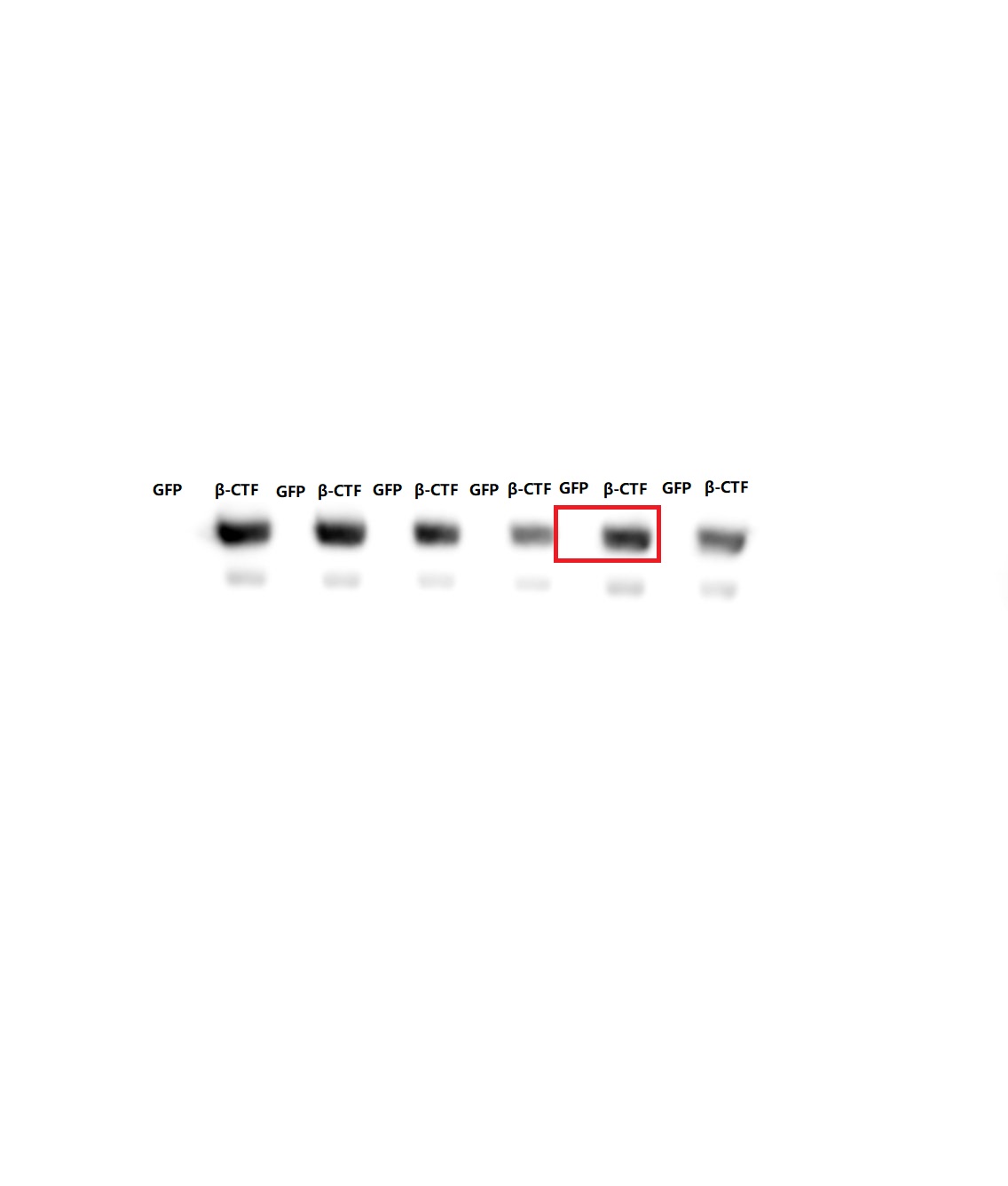

Supplement: Figure 6—source data 2. [file elife-100968-fig6-data2.zip › Figure 6E/a┬-CTF-labelled.jpg]

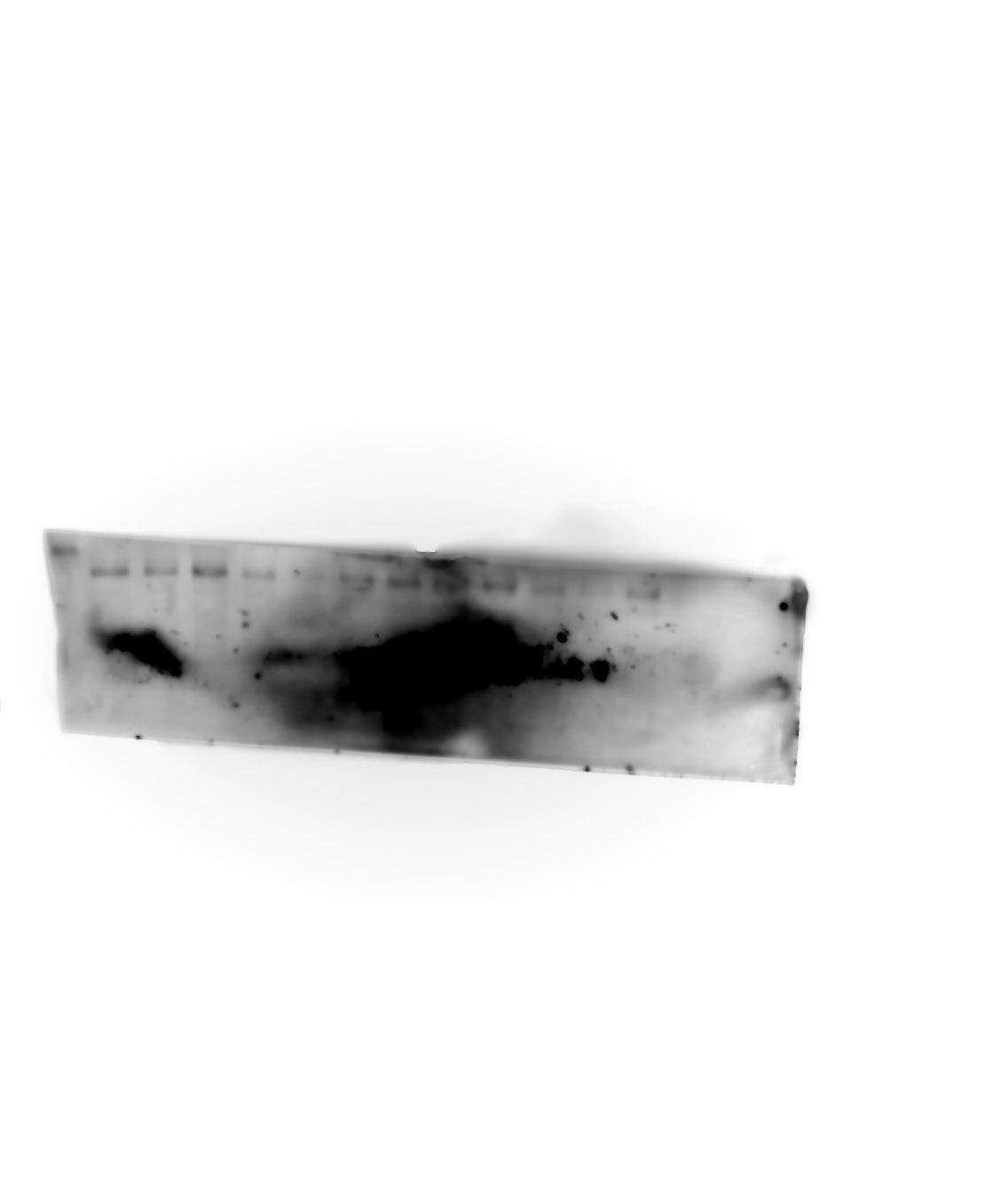

Supplement: Figure 6—figure supplement 1—source data 1. [file elife-100968-fig6-figsupp1-data1.zip › Figure 6-figure supplement 1A/GluR1-unedited gels.jpg]

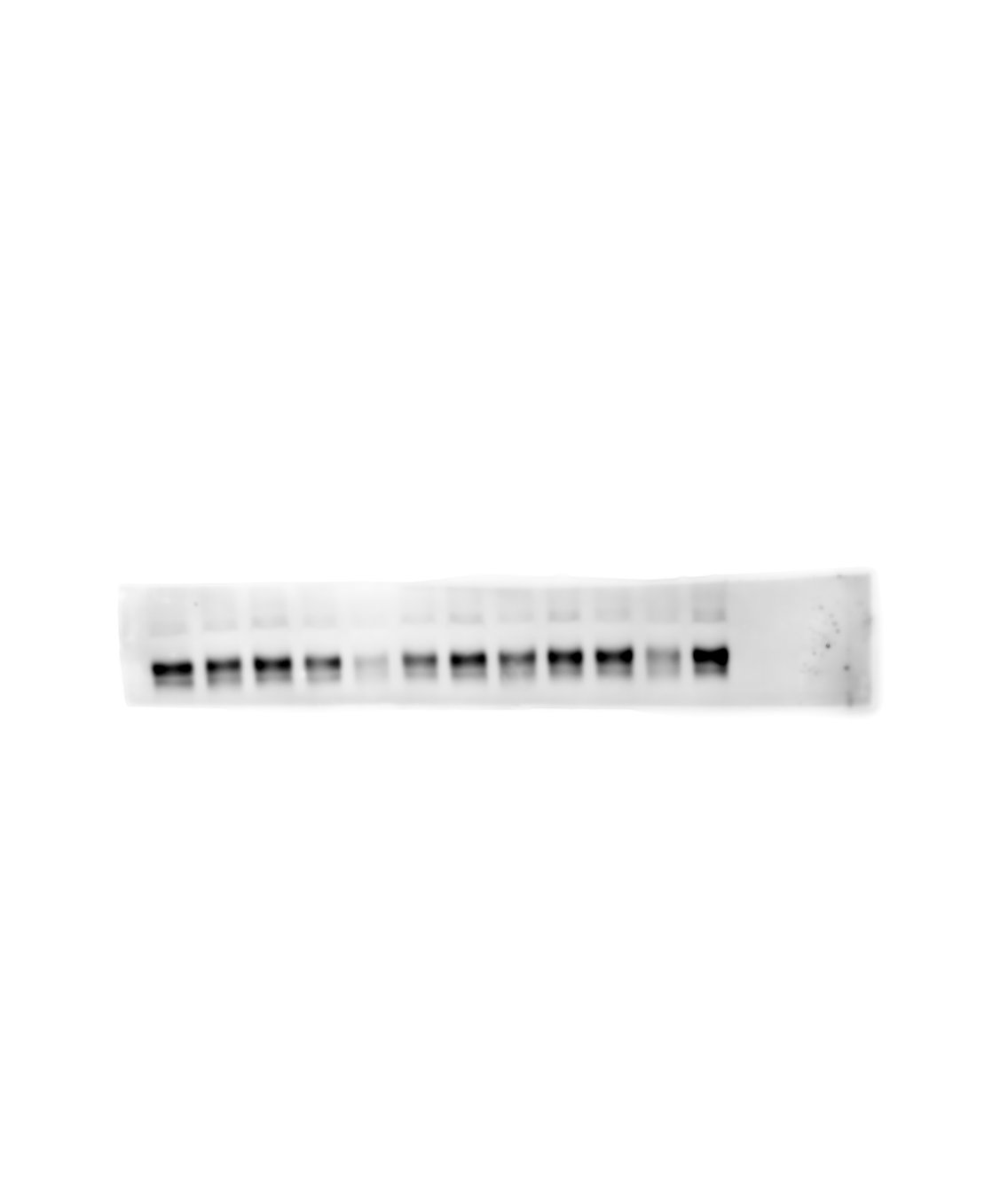

Supplement: Figure 6—figure supplement 1—source data 1. [file elife-100968-fig6-figsupp1-data1.zip › Figure 6-figure supplement 1A/Synapsin 1-unedited gels.jpg]

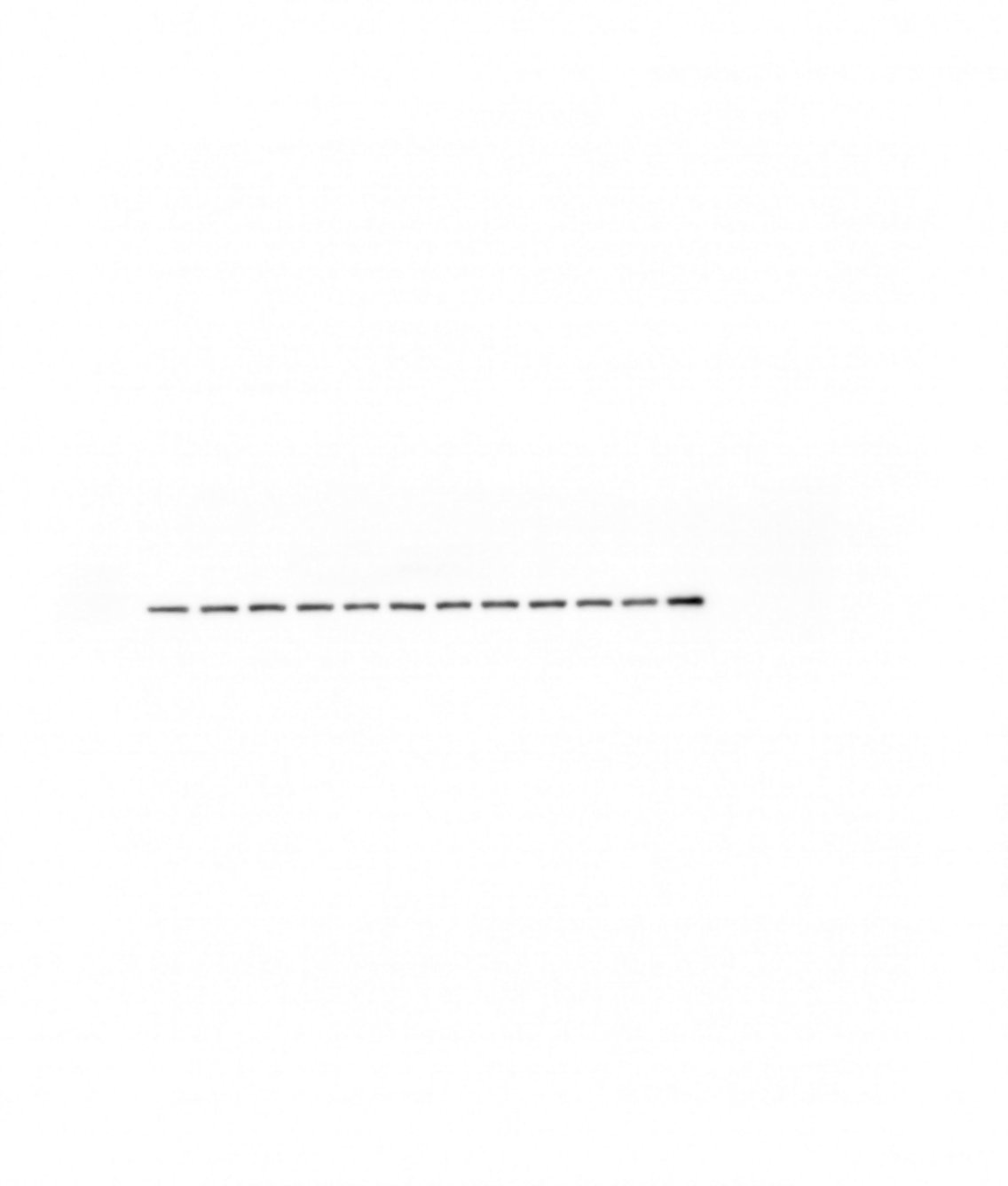

Supplement: Figure 6—figure supplement 1—source data 1. [file elife-100968-fig6-figsupp1-data1.zip › Figure 6-figure supplement 1A/a┬-actin-unedited gels.jpg]

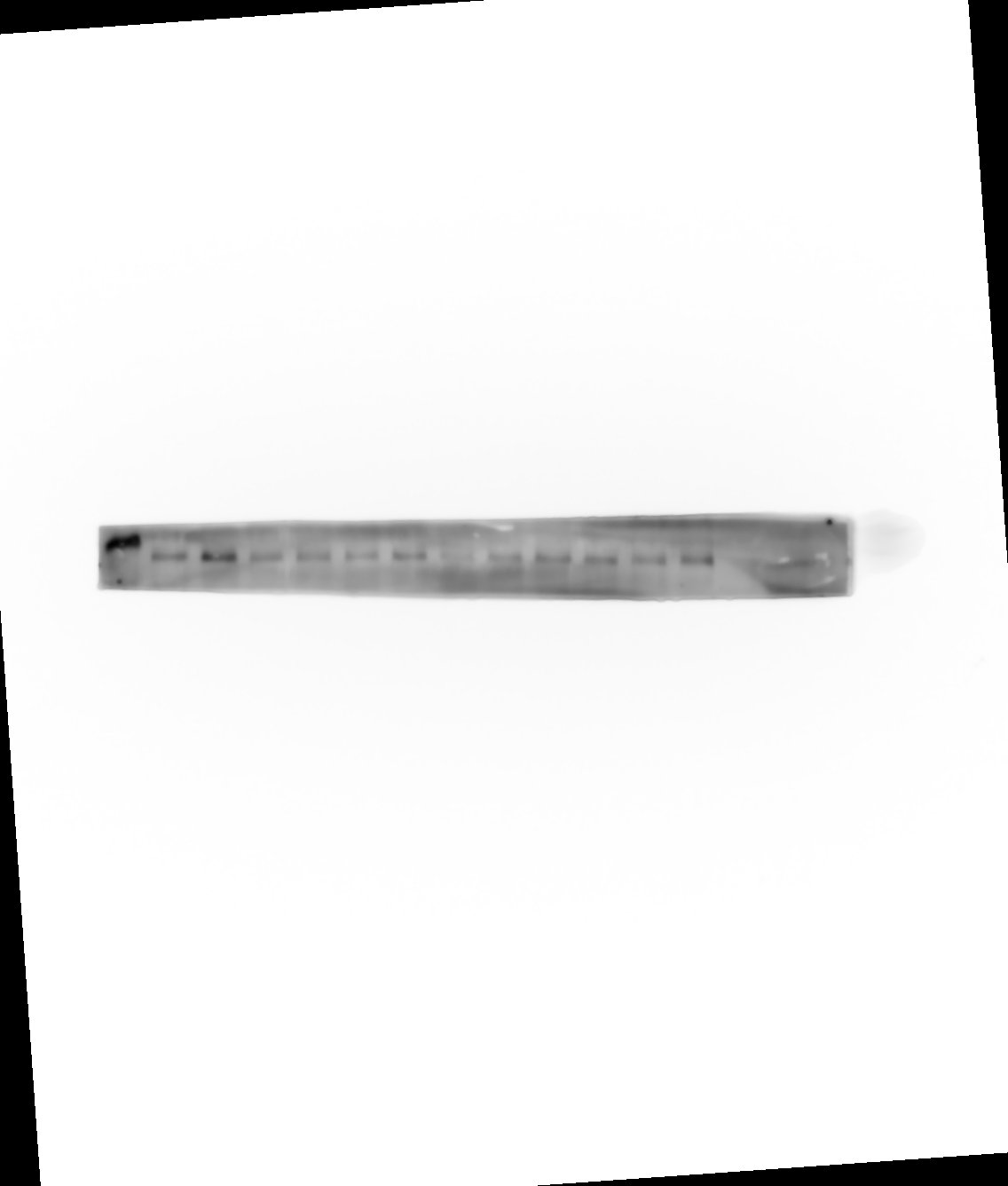

Supplement: Figure 6—figure supplement 1—source data 1. [file elife-100968-fig6-figsupp1-data1.zip › Figure 6-figure supplement 1G/GluR1-unedited gels.jpg]

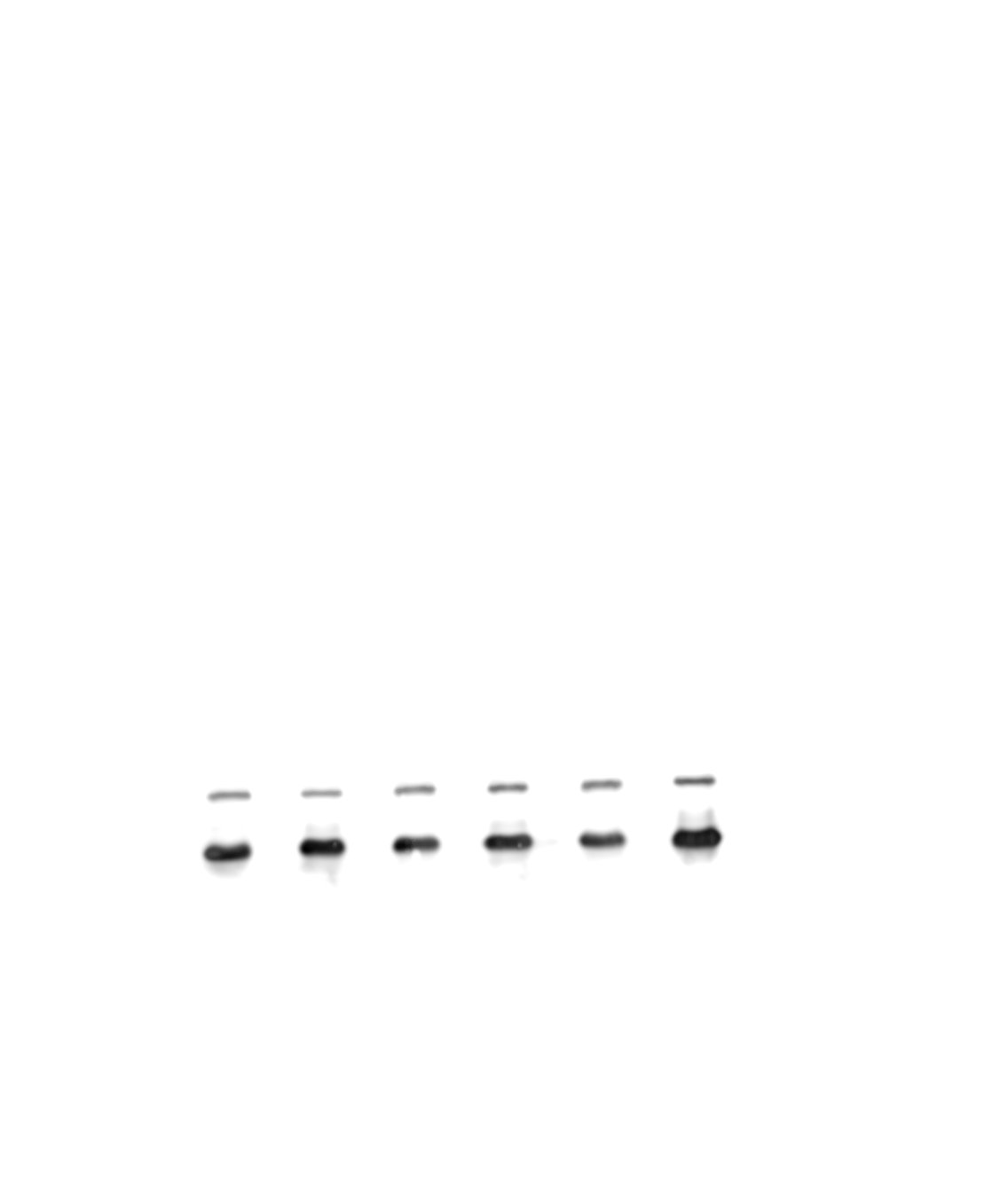

Supplement: Figure 6—figure supplement 1—source data 1. [file elife-100968-fig6-figsupp1-data1.zip › Figure 6-figure supplement 1G/Rab5-unedited gels.jpg]

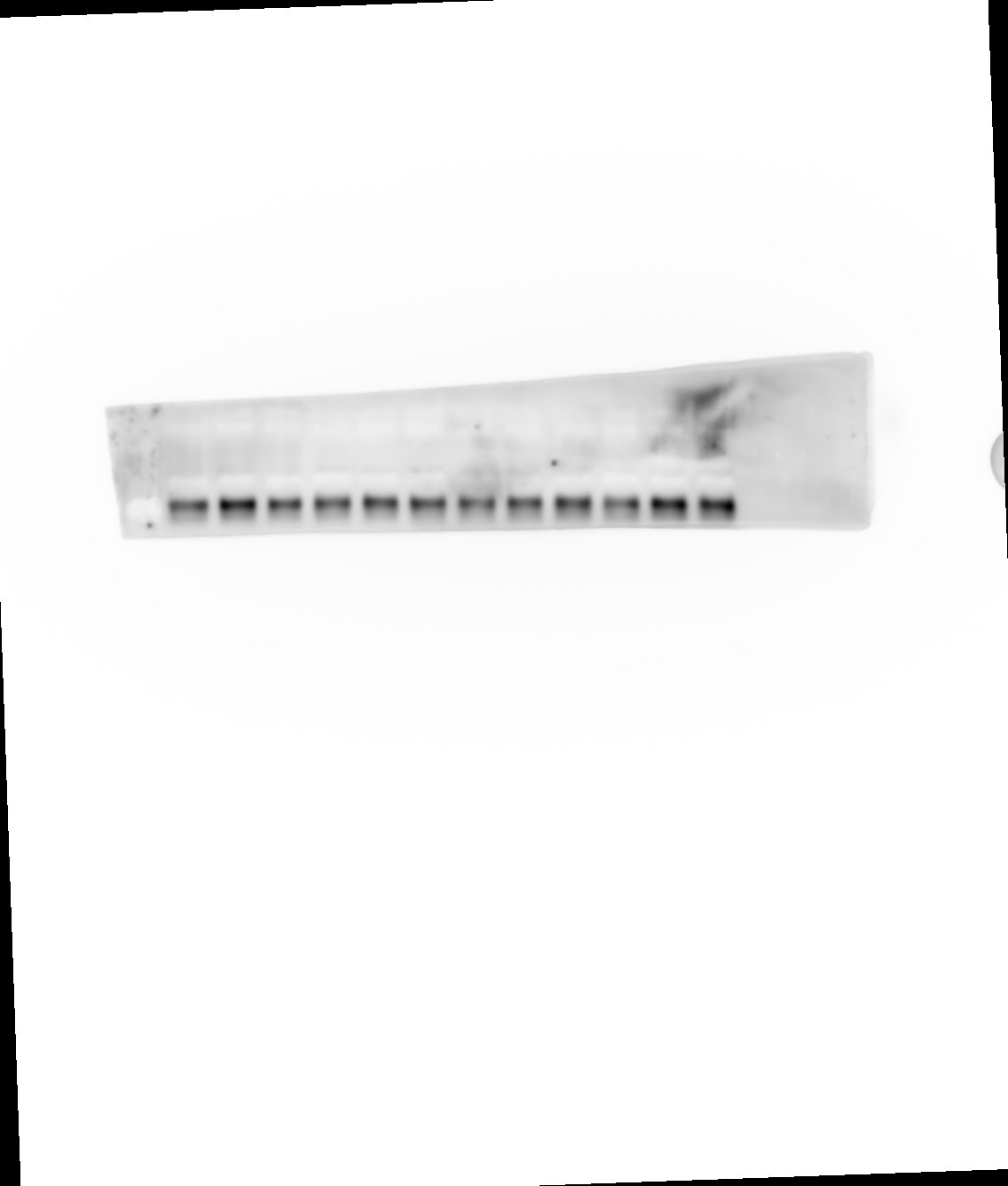

Supplement: Figure 6—figure supplement 1—source data 1. [file elife-100968-fig6-figsupp1-data1.zip › Figure 6-figure supplement 1G/Synapsin-1-unedited gels.jpg]

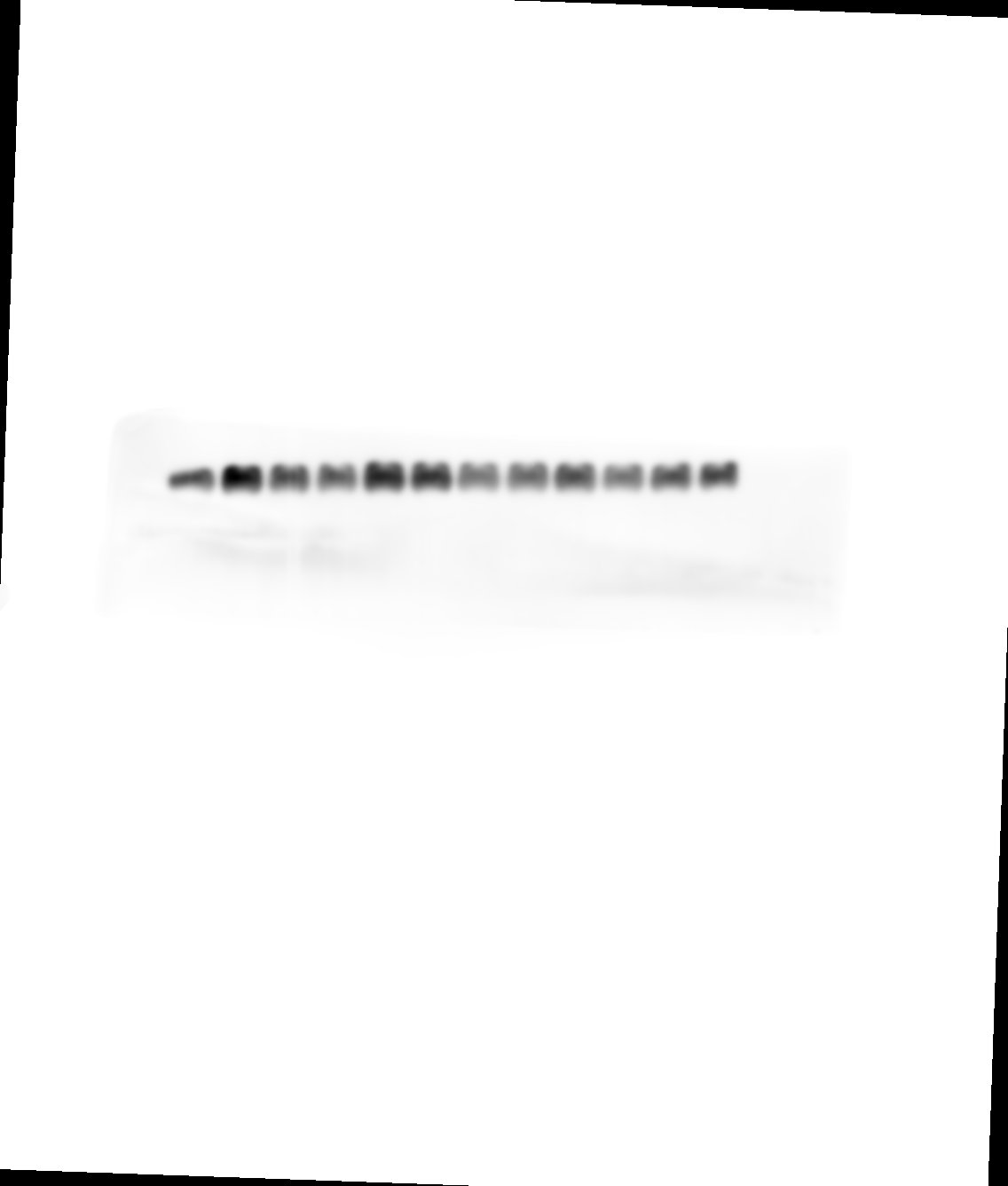

Supplement: Figure 6—figure supplement 1—source data 1. [file elife-100968-fig6-figsupp1-data1.zip › Figure 6-figure supplement 1G/VAMP2-unedited gels.jpg]

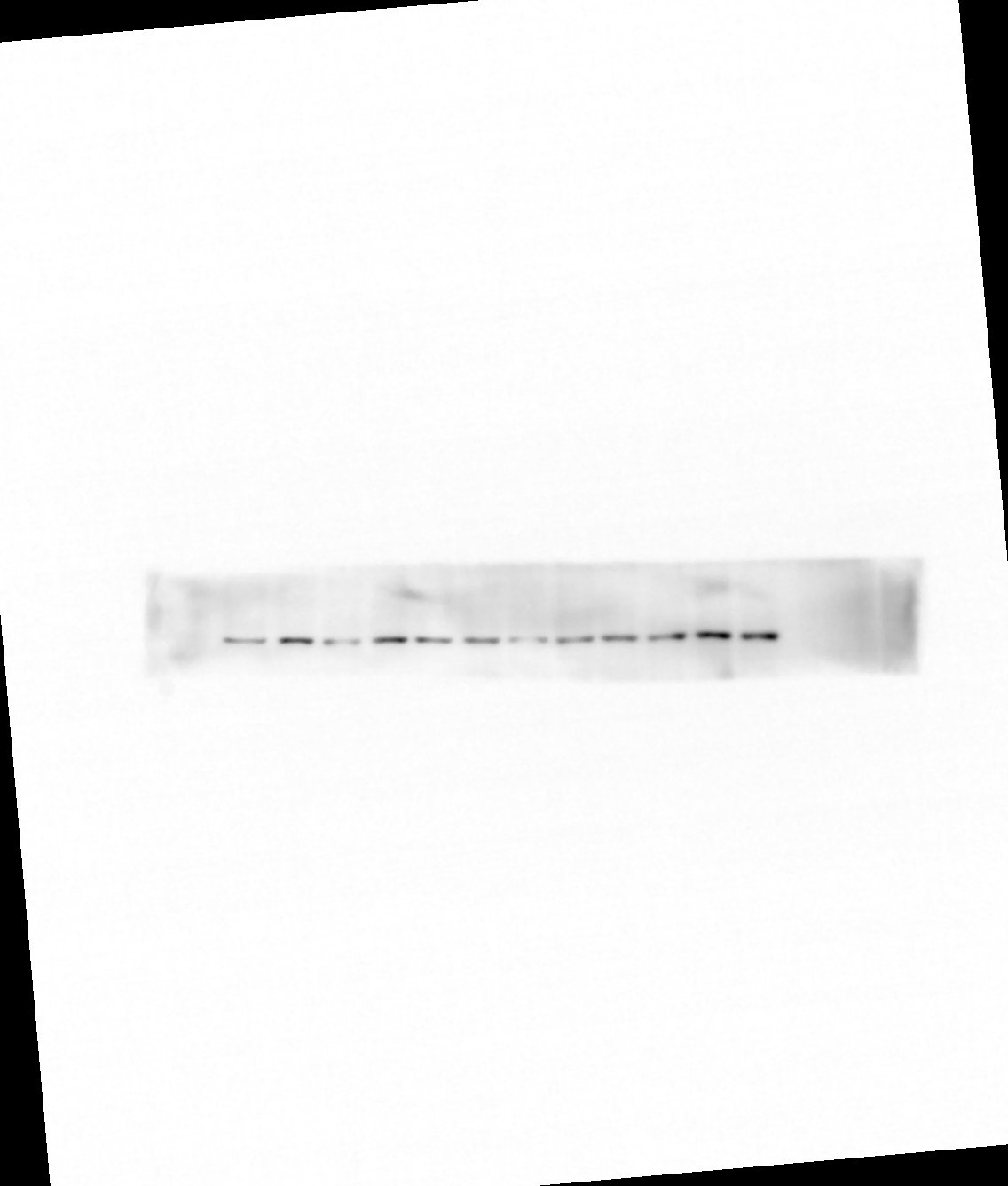

Supplement: Figure 6—figure supplement 1—source data 1. [file elife-100968-fig6-figsupp1-data1.zip › Figure 6-figure supplement 1G/a┬actin-unedited gels.jpg]

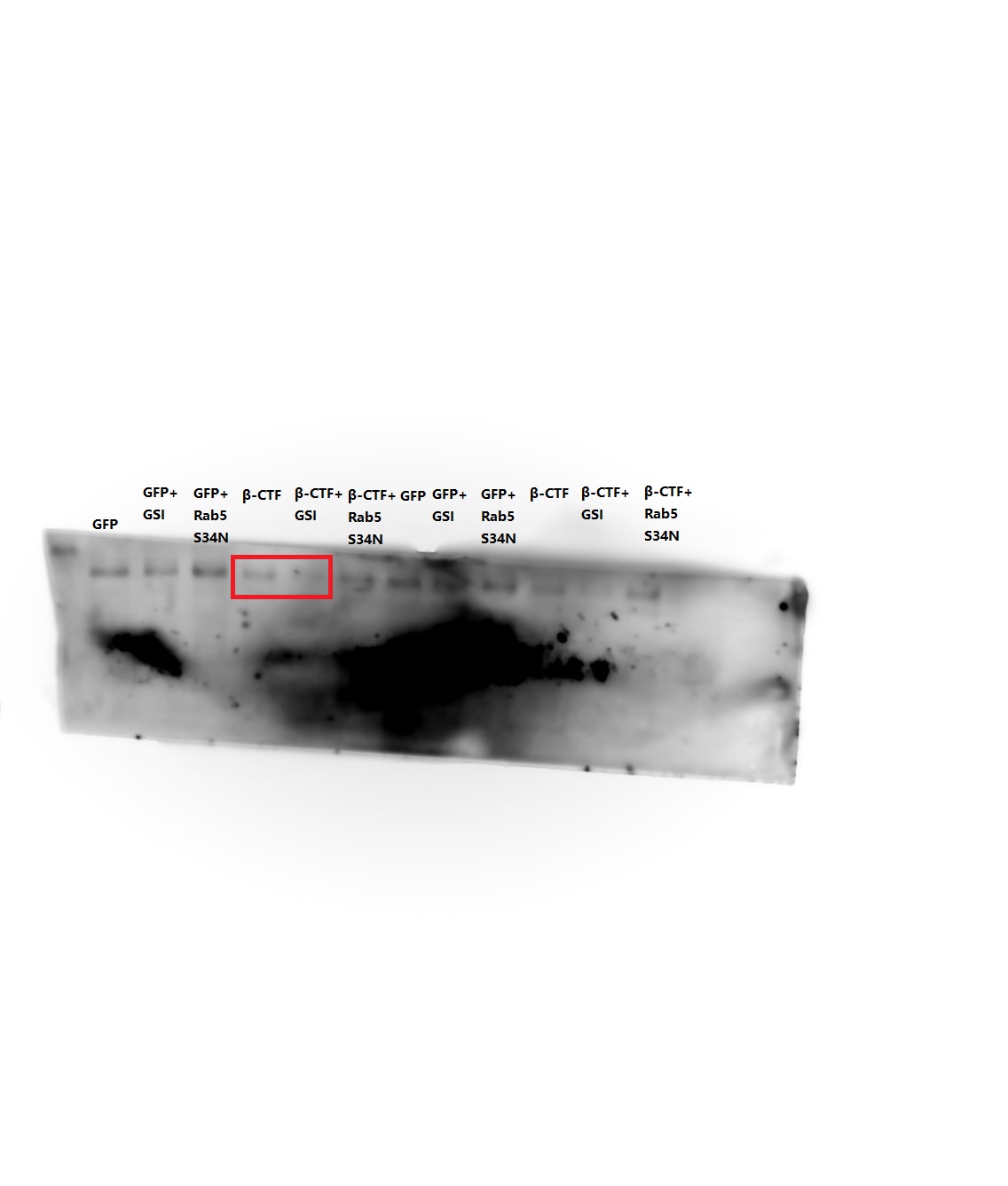

Supplement: Figure 6—figure supplement 1—source data 2. [file elife-100968-fig6-figsupp1-data2.zip › Figure 6-figure supplement 1A/GluR1-labelled.jpg]

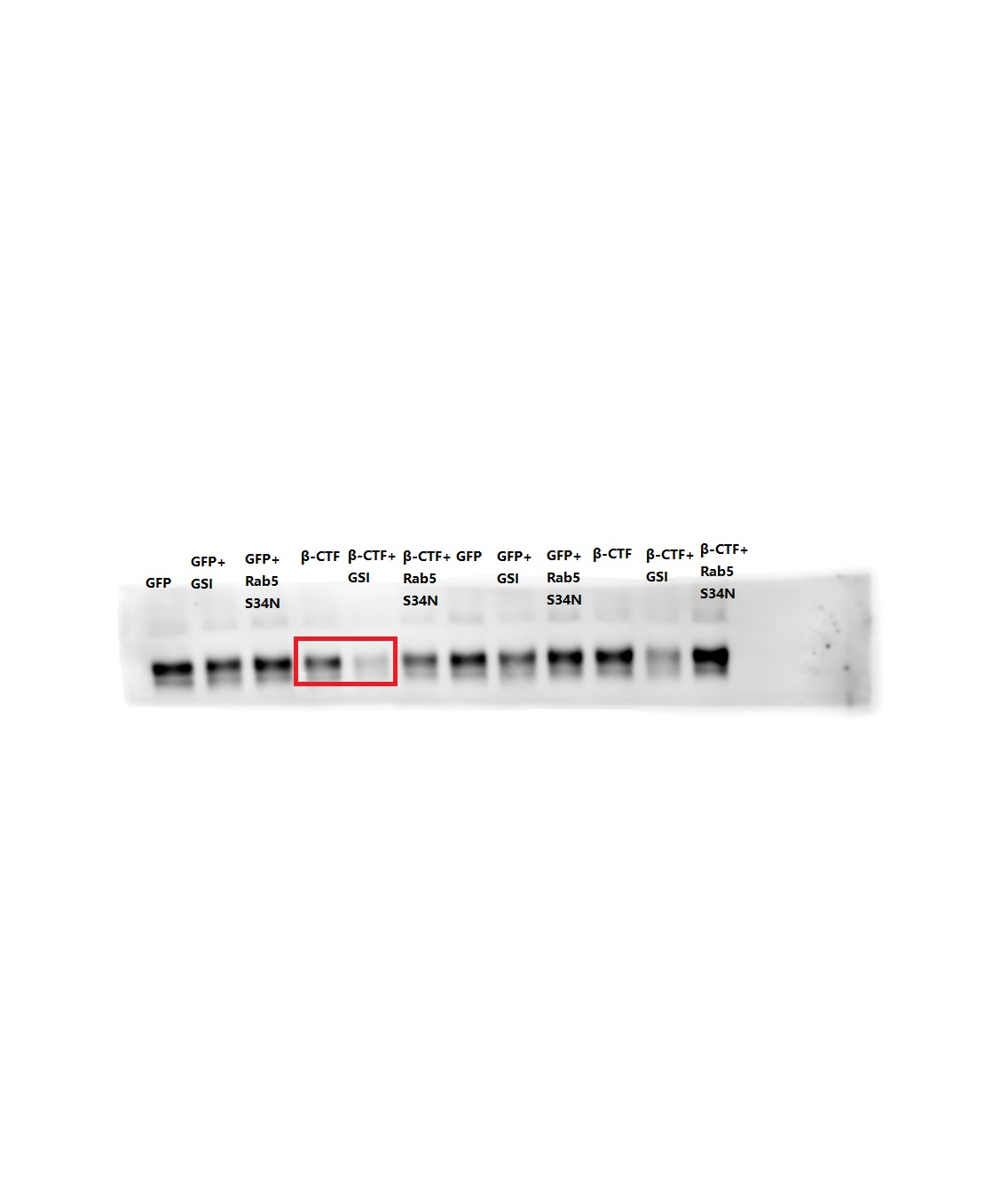

Supplement: Figure 6—figure supplement 1—source data 2. [file elife-100968-fig6-figsupp1-data2.zip › Figure 6-figure supplement 1A/Synapsin 1-labelled.jpg]

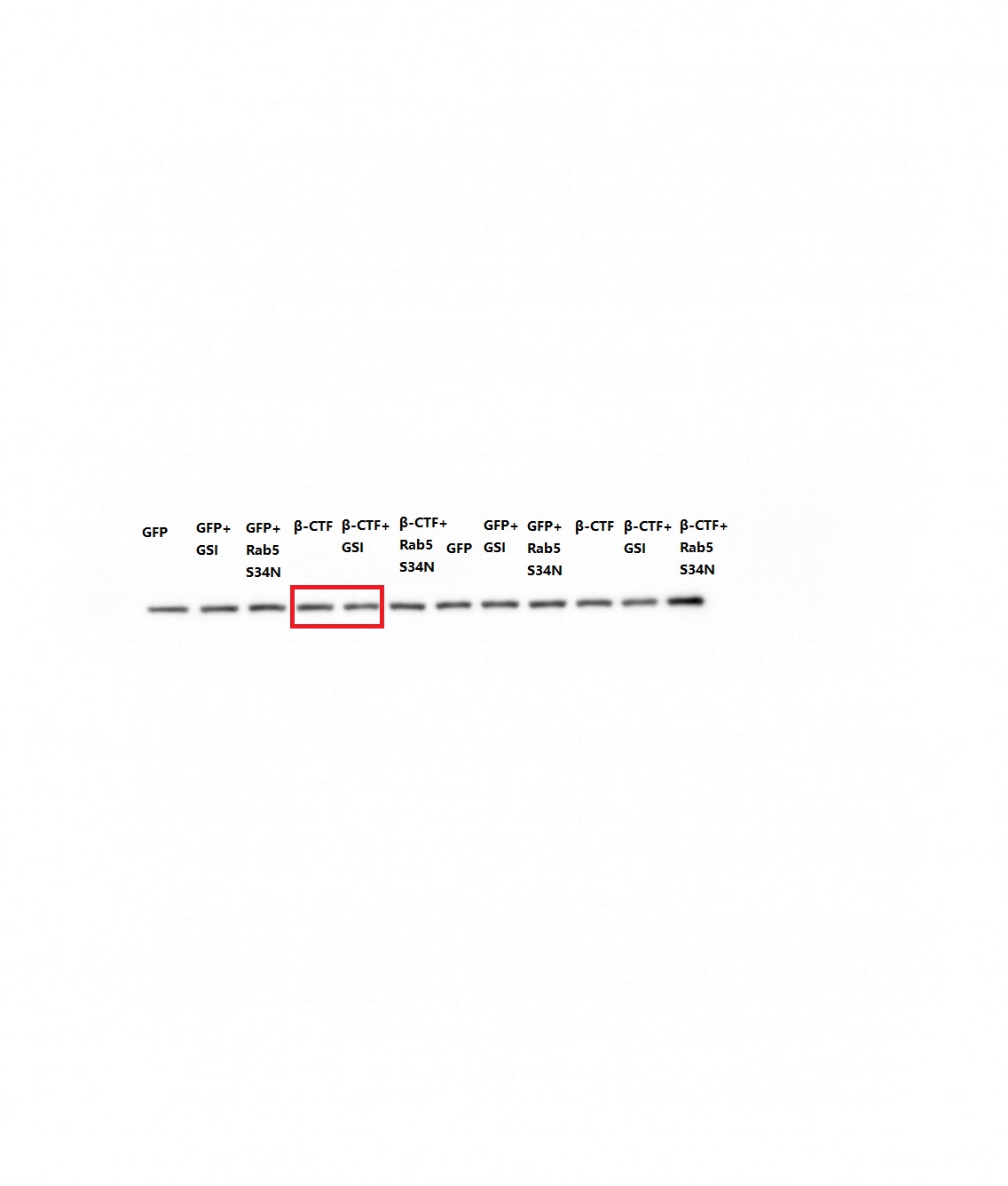

Supplement: Figure 6—figure supplement 1—source data 2. [file elife-100968-fig6-figsupp1-data2.zip › Figure 6-figure supplement 1A/a┬-actin-labelled.jpg]

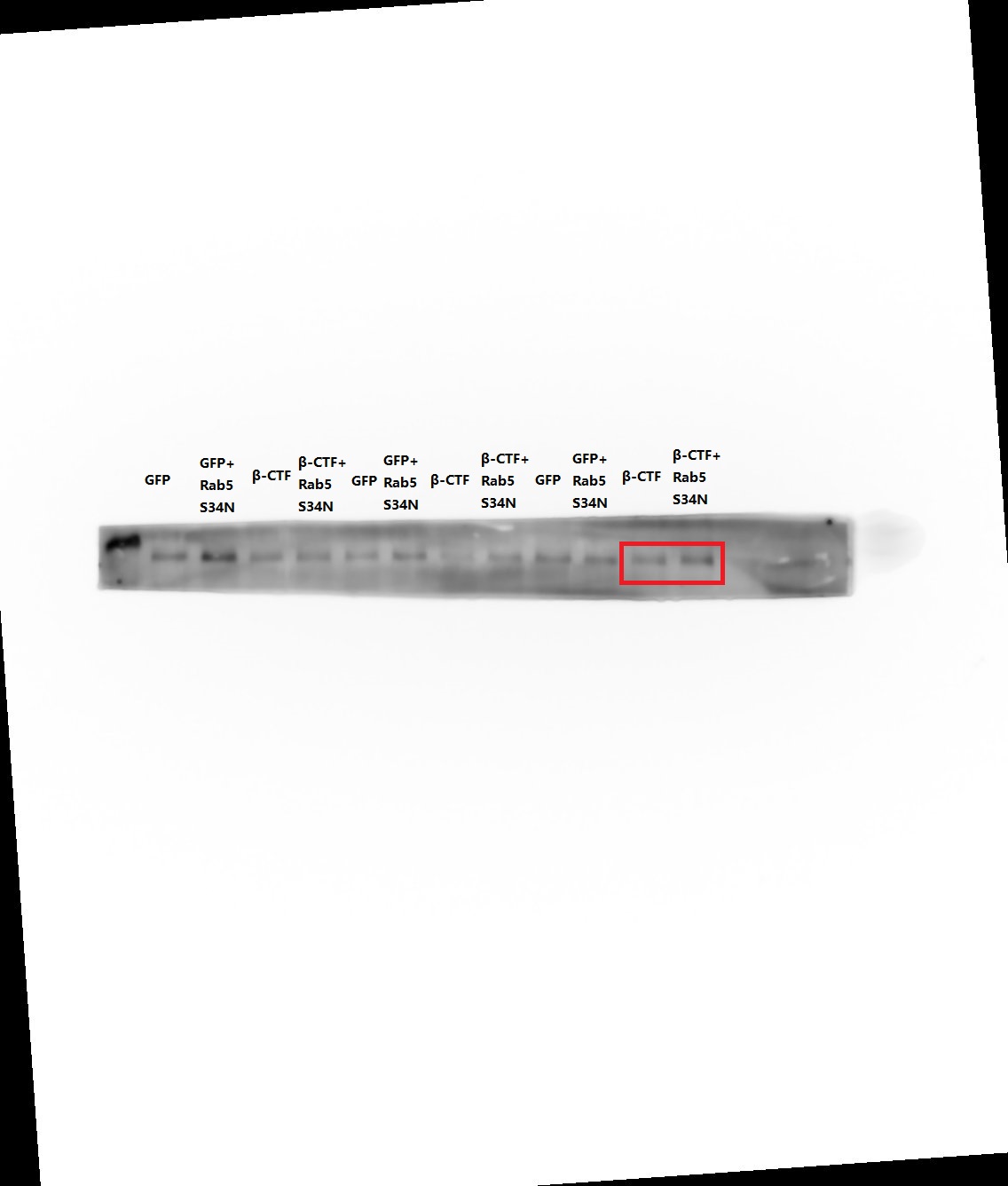

Supplement: Figure 6—figure supplement 1—source data 2. [file elife-100968-fig6-figsupp1-data2.zip › Figure 6-figure supplement 1G/GluR1-labelled.jpg]

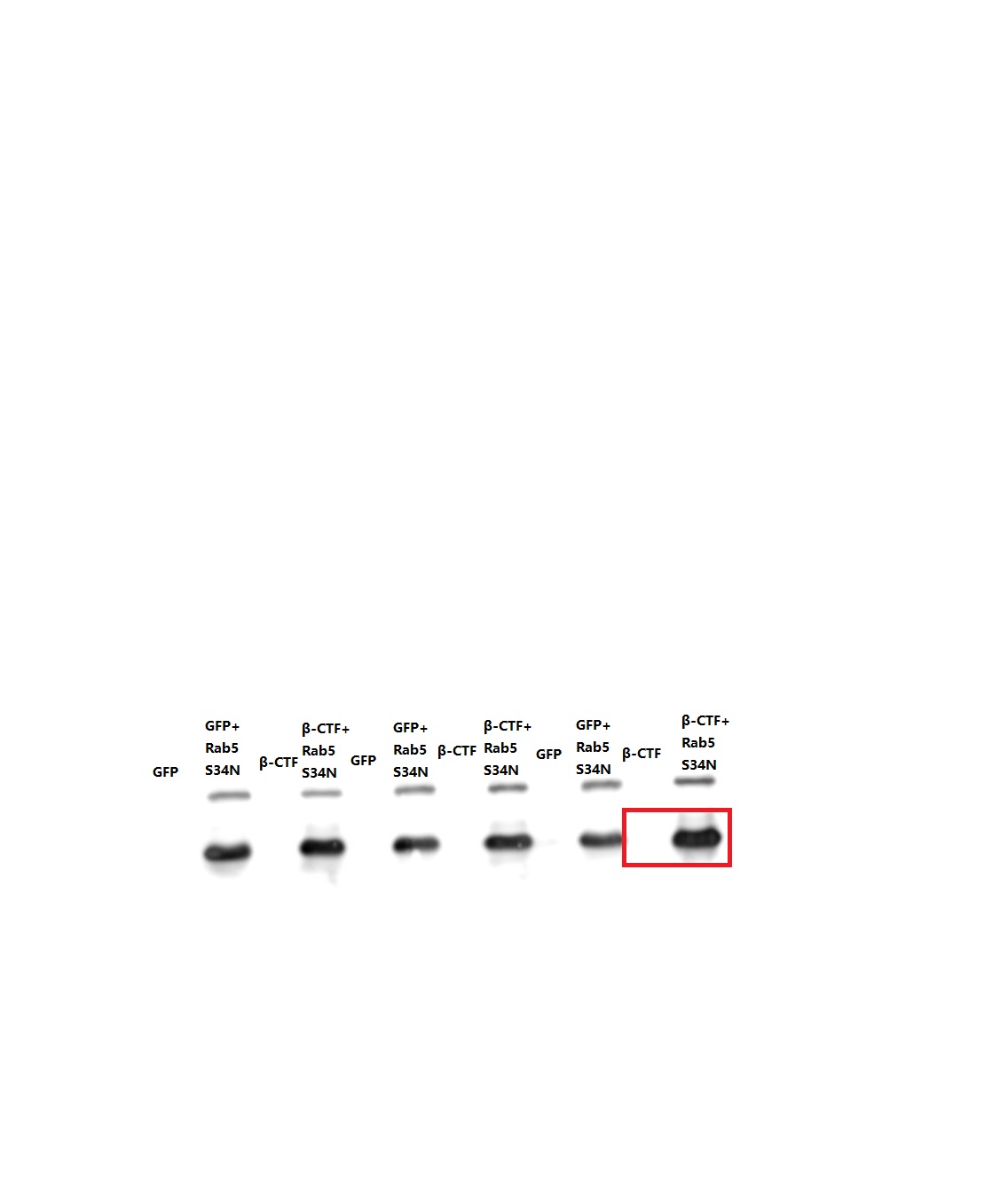

Supplement: Figure 6—figure supplement 1—source data 2. [file elife-100968-fig6-figsupp1-data2.zip › Figure 6-figure supplement 1G/Rab5-labelled.jpg]

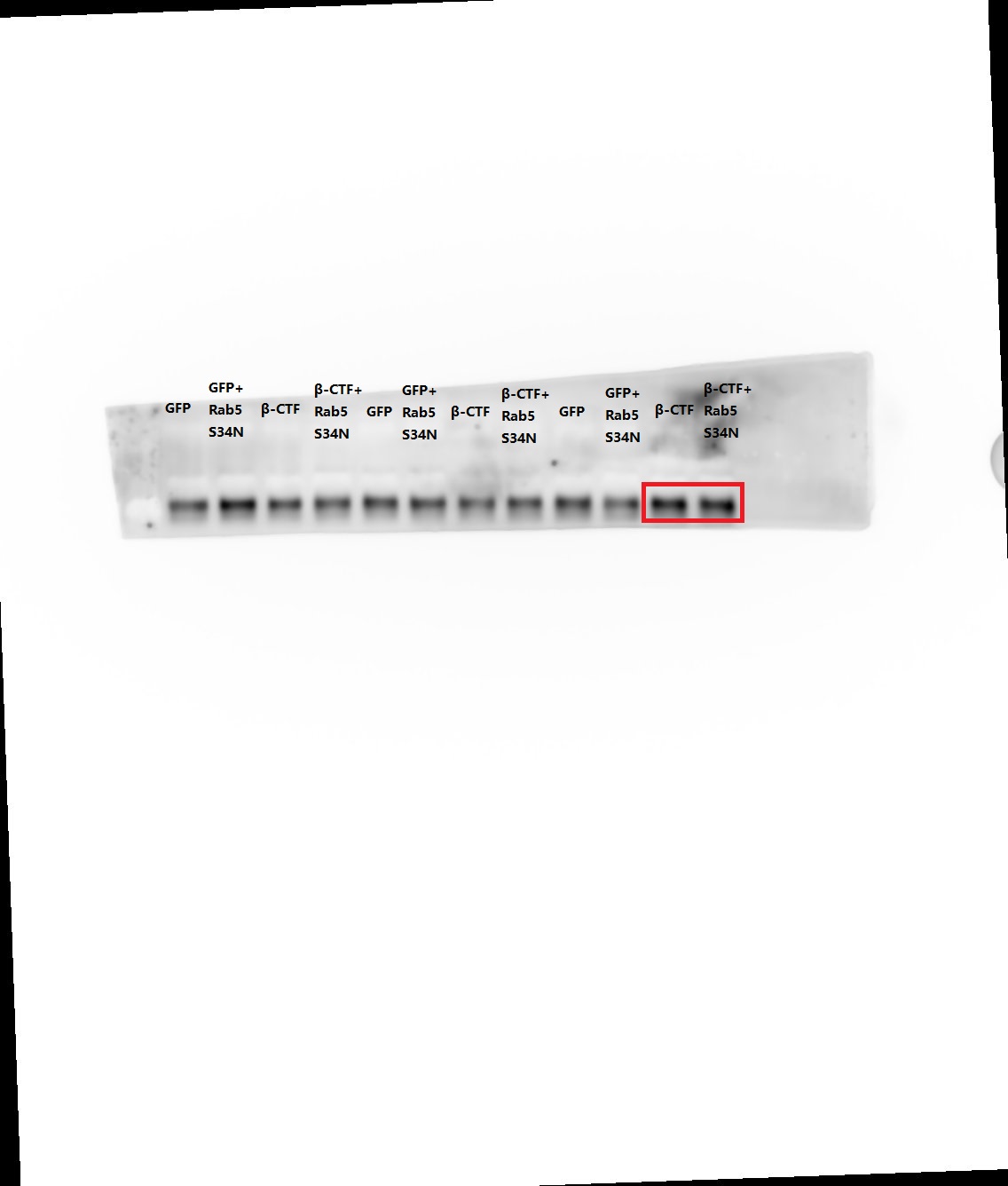

Supplement: Figure 6—figure supplement 1—source data 2. [file elife-100968-fig6-figsupp1-data2.zip › Figure 6-figure supplement 1G/Synapsin-1-labelled.jpg]

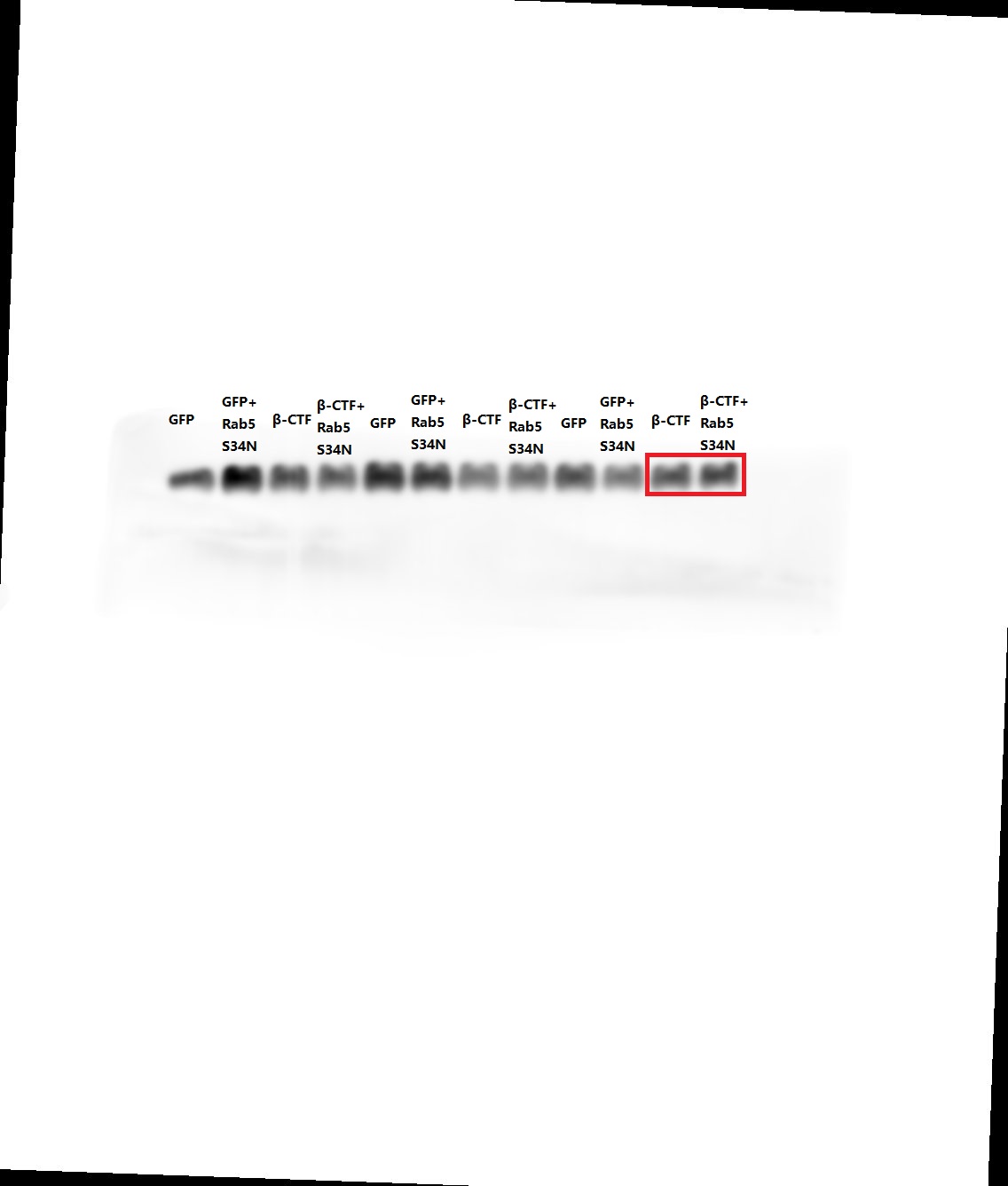

Supplement: Figure 6—figure supplement 1—source data 2. [file elife-100968-fig6-figsupp1-data2.zip › Figure 6-figure supplement 1G/VAMP2-labelled.jpg]

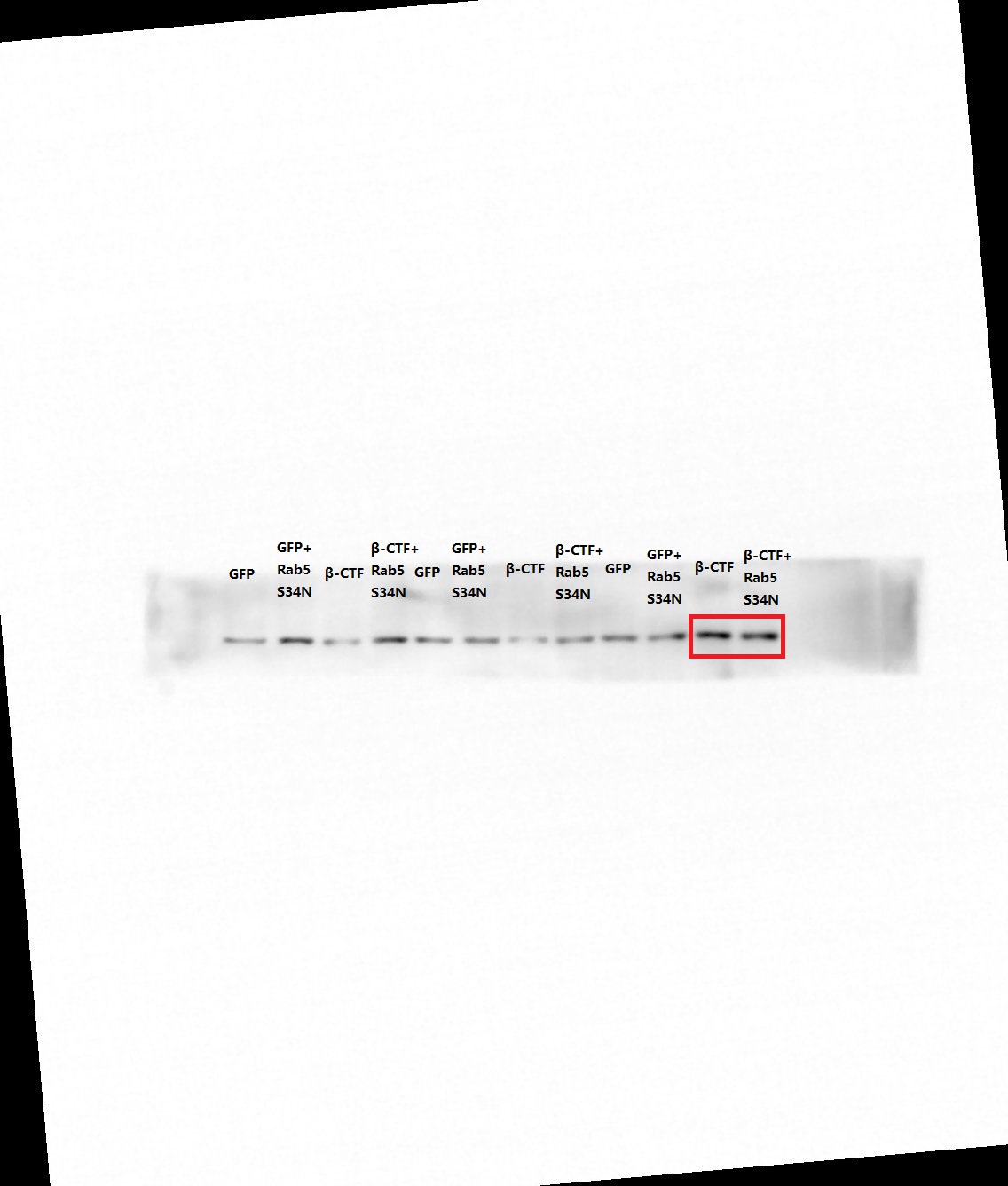

Supplement: Figure 6—figure supplement 1—source data 2. [file elife-100968-fig6-figsupp1-data2.zip › Figure 6-figure supplement 1G/a┬actin-labelled.jpg]
